# Upon Repeated Reflection: Consequences of Frequent Exposure to the Cognitive Reflection Test for Mechanical Turk Participants

**DOI:** 10.3389/fpsyg.2019.02646

**Published:** 2019-12-06

**Authors:** Jan K. Woike

**Affiliations:** Center for Adaptive Rationality (ARC), Max Planck Institute for Human Development, Berlin, Germany

**Keywords:** cognitive reflection test (CRT), professional participants, Mechanical Turk (MTurk), online research, practice effects

## Abstract

Participants from public participant panels, such as Amazon Mechanical Turk, are shared across many labs and participate in many studies during their panel tenure. Here, I demonstrate direct and indirect downstream consequences of frequent exposure in three studies (*N*_1−3_ = 3, 660), focusing on the cognitive reflection test (CRT), one of the most frequently used cognitive measures in online research. Study 1 explored several variants of the signature bat-and-ball item in samples recruited from Mechanical Turk. Panel tenure was shown to impact responses to both the original and merely similar items. Solution rates were not found to be higher than in a commercial online panel with less exposure to the CRT (Qualtrics panels, *n* = 1, 238). In Study 2, an alternative test with transformed numeric values showed higher correlations with validation measures than the original test. Finally, Study 3 investigated sources of item familiarity and measured performance on novel lure items.

## 1. Introduction

### 1.1. Professional Participants on Amazon Mechanical Turk

On Amazon's Mechanical Turk platform (MTurk) participants (called “workers”) complete small tasks (“Human intelligence tasks” or “HITs”) offered by employers (“requesters”) against monetary payment. A few years after its introduction in 2005 (Paolacci et al., 2010), academics discovered its potential as a platform for conducting research. A claimed participant pool of up to 500,000 international workers compared favorably with typical university pools regarding size and heterogeneity. Moreover, given the low average payment rates especially prevalent in the platform's early days (Ipeirotis, 2010; Paolacci et al., 2010) and the impressive speed of data collection, researchers soon embraced the platform to a degree never encountered before. MTurk has been hailed as “a revolutionary tool for conducting experiments” (Crump et al., 2013, p. 16) with the potential to transform the conduct of behavioral research. Indeed, many disciplines have begun to routinely use MTurk samples, including many subfields of psychology (Crump et al., 2013; Landers and Behrend, 2015; Chandler and Shapiro, 2016; Cheung et al., 2017).

As with any novel disruption to established sampling procedures (like computer testing and online research in earlier years), critics soon attacked research relying on MTurk participants on dimensions such as data quality, participant authenticity, and sample representativeness. Most investigators have concluded that both in terms of attention and quality, data collected on MTurk was not inferior to data collected from student and other convenience samples (Crump et al., 2013; Landers and Behrend, 2015; Hauser and Schwarz, 2016; McCredie and Morey, 2018; Coppock, 2019). Samples from established professional online panels have been found to be more representative of the general population than MTurk samples, but not to be necessarily of higher quality (Kees et al., 2017).

New—and more persistent—questions emerged, when researchers realized that participants in crowdsourced online panels had a much longer tenure in these panels than student participants with limited programs of study at research institutions. Critics argued that these “professional participants” might differ from traditional participants in criticial aspects (Dennis, 2001; Hillygus et al., 2014; Matthijsse et al., 2015). At first, the reported size of the MTurk population seemed to address this problem sufficiently, but two developments contributed to its reemergence: Stewart et al. (2015) found that the population of participants available to any given lab was far below the reported number and closer to around 7,000 participants, similar to the size of university pools.

Further, research activity on MTurk exploded with many overlapping research questions being investigated simultaneously, making it very likely that participants—who self-select into studies (Stewart et al., 2017)—were exposed to the same scenarios, tasks and test items multiple times (Chandler et al., 2014). Participants on MTurk have participated in many more academic studies on average than members of earlier panels (Stewart et al., 2017) with some evidence for decreased effect sizes for returning participants in experiments (Chandler et al., 2015).

### 1.2. Repeated Exposure to the Cognitive Reflection Test

Practice effects, increases in test performance through repeated test taking, are a common phenomenon for many cognitive tests (see e.g., Calamia et al., 2012). Many tasks on MTurk are encountered frequently by active participants, for example behavioral economics games such as the dictator or ultimatum game. Of particular concern regarding practice effects are questions with correct answers that could be learned either by repeated engagement, conversation between participants, or searching outside the platform. One of the most heavily used tasks in psychological and economic studies with memorizable correct answers is the cognitive reflection test (CRT, Frederick, 2005, the three items are shown in Table 1). Cognitive reflection is “the ability or disposition to resist reporting the response that first comes to mind” (Frederick, 2005, p. 35). The original CRT (Frederick, 2005) consists of three questions that have intuitive and commonly given answers that turn out to be false upon further reflection (Toplak et al., 2014). The signature question is the bat-and-ball question (I1 in Table 1).

**Table 1:** Original CRT items and items presented in Studies 1–3: study, variant name, item text, correct solution, and intuitive solution.

**Study**	**Variant**	**Question**	**Corr**.	**Int**.
CRT	I1	A bat and a ball cost $1.10 in total. The bat costs $1.00 more than the ball	$0.05	$0.10
		How much does the ball cost?		
	I2	If it takes 5 machines 5 min to make 5 widgets, how long would it take 100 machines	5 m	100 m
		to make 100 widgets?		
	I3	In a lake, there is a patch of lily pads. Every day, the patch doubles in size	47d	24d
		If it takes 48 days for the patch to cover the entire lake, how long would it take for the		
		patch to cover half of the lake?		
Study 1	Original	A bat and a ball cost $1.10 in total. The bat costs $1.00 more than the ball	5	10
		How much does the ball cost? [in cents]		
	Complementary	A bat and a ball cost $1.10 in total. The bat costs $1.00 more than the ball	105	100
		How much does the bat cost? [in cents]		
	Trivial	A bat and a ball cost $1.10 in total. The bat costs more than the ball. It costs $1.00	10	10
		How much does the ball cost? [in cents]		
	Transformed	A golden bat and a golden ball cost $5,000 in total. The golden bat costs $4,000 more	500	1,000
		than the golden ball. How much does the golden ball cost? [in $]		
Study 2 (CRTt)	T1	A golden bat and a golden ball cost $5,000 in total. The golden bat costs $4,000 more	500	1,000
		than the golden ball. How much does the golden ball cost? [in $]		
	T2	If it takes 10 machines 10 min to make 10 widgets, how long would it take	10	1,000
		1,000 machines to make 1,000 widgets [in minutes]?		
	T3	In a lake, there is a patch of lily pads. Every day, the patch doubles in size	38	10
		If it takes 40 days for the patch to cover the entire lake, how long would		
		it take for the patch to cover a quarter of the lake [in days]?		
Study 3	I1	A bat and a ball cost $1.10 in total. The bat costs $1.00 more than the ball	5	10
		How much does the ball cost? [in cents]		
	I2	If it takes 5 machines 5 min to make 5 widgets, how long would it take	5	100
		100 machines to make 100 widgets? [in minutes]		
	N1	Peter has four friends. Together they are able to carry 40 boxes.	160/168	200
		If Peter had 20 friends instead, how many boxes would they be able to carry?		
	N2	If you divided a long baguette by four cuts into even pieces, each piece would	10	9
		be 18 cm long. How long would a piece be if you did it with eight cuts? [in cm]		

While many participants prize the ball at ten cents, a quick check will show that this would place the bat at $1.10 adding up to a total price of $1.20. The correct solution is given by

(1) $$\begin{array}{r} x+\left(x+\$1.00\right)=\$1.10\Leftrightarrow 2x=\$0.10\Leftrightarrow x=\$0.05. \end{array}$$

The first item is followed by two similar items that can trick respondents into false, yet intuitive responses.

Toplak et al. (2011) described the CRT as a “measure of the tendency toward the class of reasoning error that derives from miserly processing” (p. 1284). CRT scores have been shown to correlate with numeracy (Cokely et al., 2012), verbal intelligence (Bialek and Pennycook, 2017), and SAT^1^ scores (Frederick, 2005), but also skepticism (Pennycook et al., 2015), religious disbelief (Gervais and Norenzayan, 2012; Stagnaro et al., 2019), economically advantageous decision-making (Corgnet et al., 2015). and lower risk aversion (Noori, 2016). Baron et al. (2015) considered the CRT to be “one of the most useful measures in the study of individual differences in thinking, judgments, and decisions” (p. 266).

Both the items and their solutions have been popularized since the test's introduction in books, classrooms and newspaper articles (e.g., Postrel, 2006; Lubin, 2012). The popularity of the test (and the associated research) is perceived as a double-edged sword by cognitive researchers. It has generated a rich base of data for comparison, but might also lead to increased familiarity with the items. In an earlier study, Goodman et al. (2013) did not find significant differences between an MTurk and a community sample, and MTurk participants scored lower, on average, than student samples. Given the likelihood of repeated exposure to the test items, Toplak et al. (2014, p. 149) saw “problems on the horizon for the CRT going into the future.” It can presently be assumed that the test has been encountered by the typical MTurk participant (Stewart et al., 2017). In a study in 2015, more than 75% of MTurk participants reported to have seen it before (Hauser and Schwarz, 2015), with similar results in Haigh (2016) with a sample of online volunteers and participants on Prolific Academic. Bialek and Pennycook (2017) found in an analysis of six studies with a total of about 2,500 participants that average scores of participants with pre-exposure were substantially higher than scores from naive participants (*M* = 1.65 vs. *M* = 1.02 for all participants, *M* = 1.70 vs. *M* = 1.20 for MTurk participants), and higher than the scores of Princeton or Harvard students (Frederick, 2005). Haigh (2016) reported that the majority of their participants reached the maximum score, with higher scores for participants with prior exposure (*M* = 2.36 vs. *M* = 1.48). For the bat-and-ball problem alone, the relative frequency of correct solutions increased from 40.7% to 73.8%. Thomson and Oppenheimer (2016) observed an even higher degree of prior exposure (94%) in a sample of MTurk participants with master qualification. Participants in Argentina (Campitelli and Labollita, 2010, *M* = 0.66) and Australia (Campitelli and Gerrans, 2014, *M* = 0.94) had lower average scores than all groups of MTurk participants, even those in Goodman et al. (2013).

How do higher scores impact current research? Different mechanisms and behaviors could, in theory, be responsible for score increases: (a) active search for solutions^2^, (b) accidentally finding item solution in classrooms, books, or online, (c) reflecting on the items after the task, (d) suspecting a hidden layer of complexity when encountering a seemingly simple item for the second time. The impact on test validity would depend on the mechanism and the degree of insight into the problem. Chandler et al. (2014) observed, for example, that the number of previous HITs on MTurk correlated with the performance on the original CRT items, but not with the performance on variants of these items, consistent with a rather narrow learning of solutions. For these reasons Goodman and Paolacci (2017, p. 9) called the CRT a “confounded measure” on MTurk and Haigh (2016) expressed concerns about the future of the test, arguing that the groups that were most likely to be exposed to the solutions in popular media or university classes were exactly the groups most likely to be studied with the CRT. Thomson and Oppenheimer (2016) called MTurk a “corrupted subject pool for cognitive reflection” (p. 102), and addressed this problem with others (Toplak et al., 2014) by extending the test's item set.

Following these expressions of concern, several authors recently tested the impact of prior experience on CRT validity empirically. These studies replicated the score increase with repeated exposure, but did not find a decrease in test validity. In their set of studies, Bialek and Pennycook (2017) found similar correlations between CRT and target variables for experienced and inexperienced participants and concluded that “[t]he CRT is robust to multiple testing, and there is no need to abandon it as an individual difference measure.” This advice was echoed by Meyer et al. (2018) and Stagnaro et al. (2018).

Meyer et al. (2018) analyzed data for 14,000 participants across experiments featuring the CRT and found substantial score increases only for those that actively remembered the items. At the same time, it should be noted, that this percentage increased from about 54% to 92% with repeated participation (Meyer et al., 2018, calculated from **Table 4**). The authors found a positive correlation between performance and remembering, and, more importantly, that performance after one or more exposures was still a good proxy of initial performance: Correlations with SAT scores and general intelligence tests did not suffer. The authors concluded for the CRT that “in the most heavily exposed population, scores exhibit ample variance, are surprisingly stable, and retain their predictive validity, even when they change” (p. 249).

Stagnaro et al. (2018) re-analyzed eleven studies with over 3,000 participants who participated in multiple studies and confirmed a high correlation between first and last CRT scores. In addition, they demonstrated that CRT scores at the two most extreme points in time (with a median of 221 days apart) each enabled the prediction of target variables measured at both times. While 25.9% of participants achieved higher and only 9.8% lower scores at the latter point (Stagnaro et al., 2018, calculated from Table 1, upper panel), they found “strong evidence that performance on CRT is stable over time” (p. 265), based on a test-retest correlation of *r* = 0.81.

Finally, Raoelison and De Neys (2019) directly tested the effect of repeated exposure to the bat-and-ball question within one experiment by confronting participants in sequence with 110 problems including 50 variants of the task. In their analysis of a sample of 62 participants, 38 gave incorrect and 14 correct responses from start to finish. Only the remaining 10 seemed improved during the experiment. From one perspective, this illustrates the robustness of responses, as most participants behaved consistently throughout the experiment. On the other hand, of those participants who could learn, 10 of 48 (21%) profited from repeated exposure, which again could be counted as evidence for a practice effect.

### 1.3. A Test Case for Repeated Exposure in Crowdsourced Research

In unsupervised test settings and on the internet, item exposure has the potential to compromise items (Tippins et al., 2006; Burke, 2009; Guo et al., 2009). Direct sources for indirect learning by MTurk participants are online discussion boards and sites set up to allow for worker interaction and platform-related information transfer. According to Stewart et al. (2017), 60% of the workers used forums and 10% reported they had direct contacts with other MTurk participants. Most boards promote norms of not disclosing experimental details to protect the use of MTurk for academic research, but it is easy to find solutions to the bat-and-ball problem online. On the site TurkerNation, Milland (2015) lists the correct answers to the CRT as an example of common knowledge due to repeated exposure. Learning the solution from this post would not require cognitive reflection^3^.

Here, I treat the CRT as a test case for studying the consequences of the repeated use of experimental stimuli or item pools in large online participant pools. Most previous studies on repeated exposure to the CRT focused on the effect of familiarity on the validity and reliability of scores measured with items from the original material. Learning effects, as argued above, can be considered relatively benign, if they result from direct learning and cognitive reflection on the items. Learning is likely to be less benign if it results from (mindless) memorization of indirectly learned numbers. Given the prevalence of the task, opportunities for indirect learning possibly increase over time. One way to distinguish between mindless memorization and genuine learning as explanations for practice effects, is the use of parallel test forms (Rapport et al., 1997; Davey and Nering, 2002; Bartels et al., 2010; Calamia et al., 2012), which has been found to decrease practice effects across many studies (Kulik et al., 1984b; Benedict and Zgaljardic, 1998; Beglinger et al., 2005). This approach will be explored throughout the three studies in this manuscript.

As a specific feature of the testing environment on MTurk, both direct and indirect learning related to the CRT can easily have consequences for participants' performance in other tasks. The ecological environment of MTurk is unique in that participants have prior experience with potentially thousands of academic studies that might have contained stimuli that are variants of or even merely resemble stimuli or experimental conditions featured in any given MTurk study.

Study 1 focused on responses to the signature item of the CRT and three item variants, demonstrating the existence of memorization and task confusion on MTurk in contrast to a comparable survey population. Study 2 used a transformed variant of the CRT to address these concerns and presented encouraging results. Study 3 directly addressed sources of memorization and tested the viability of entirely novel items on the platform.

## 2. Study 1: Comparison of Item Variants

In Study 1, MTurk participants faced one of four variants of the bat-and-ball question—the original and more or less subtle variations. The bat-and-ball question has arguably received the most attention and publicity of the three tasks (Bago and De Neys, 2019). The variants were designed to explore participants' tendency to transfer correct (and false) solutions to variants of the original task and to explore downstream consequences of exposure for repeated and related tasks—in terms of both process and outcome changes.

### 2.1. Research Questions

The design of Study 1 was guided by several research questions that it was intended to answer. The overarching question is Research Question 1:

Research Question 1. *How do MTurk participants respond to the bat-and-ball problem?*

This question can be addressed on several levels: On the response level, I was interested in the distribution of responses, in particular the relationship between intuitive and correct responses. On the process level, the theoretical conceptualization of the CRT allows predictions about differences in response times between respondents falling into different answer categories.

To gain more detailed insight into the process, Study 1 employed several item variants. Two of these variants closely resembled the original item, but differed in crucial aspects. Observing reactions to these items could address the first part of Research Question 2. Specifically, answers expected for the original item could only be expected to occur for these variants, if participants relied on memorized answers and did not closely read the presented item. A third variant was created that differed visibly from the original and varied the numbers. Analyzing responses to this variant allowed to address the second part of Research Question 2.

Research Question 2. *How do participants react to subtle variations of the original item and can they generalize the solution to a transformed problem?*

Participants were also asked about the amount of work they had completed on the platform and whether they had encountered the bat-and-ball problem before. This allowed to analyze the relationship between general and specific experience and response patterns (Research Question 3).

Research Question 3. *How are responses influenced by panel tenure and exposure to the CRT?*

Finally, a second sample from a different platform was analyzed to address the question whether the obtained results are limited to MTurk or rather a broad phenomenon that generalized beyond the platform (Research Question 4).

Research Question 4. *Do the findings generalize to a different platform?*

### 2.2. Methods

#### 2.2.1. Sample

The study was conducted on MTurk as part of a series of HITs in 2016. The HITs were announced to last 12–25 min for most participants and offered a fixed payment between $1.00 and $1.10 with an average bonus payment of $0.60–$0.75. Participation was restricted to US participants. Most HITs (*n*_1_ = 1, 966) required a minimum percentage of 95% accepted HITs and a minimum of 50 completed HITs^4^. In addition, 395 participants were recruited without this qualification. A group of 1,186 participants passed one of two consecutive attention checks, while 780 participants were not required to pass attention checks^5^. Participants were on average 34.9 years old (*SD* = 11.7 years), and 54.5% categorized themselves as female (44.9% as male). The median number of previously completed HITs was 1,000 (*M* = 11, 135, *SD* = 61, 108). The median of weekly time spent on the platform was 10 h. In all studies, participants gave informed consent at the start of the survey, and all studies were approved by the IRB at the Center for Adaptive Rationality in Berlin.

#### 2.2.2. Survey Questions

The CRT item was presented within a Qualtrics survey. MTurk Participants were randomly assigned to one of four variants of the bat-and-ball problem (see Table 1): (1) the original (*n*_1.1_ = 457), (2) the trivial (*n*_1.2_ = 479), (3) the complementary (*n*_1.3_ = 473), and (4) the transformed variant (*n*_1.4_ = 557). In addition to the collection of responses and response times, all participants were asked to indicate whether they had encountered the item (or a similar item), before. All items are listed in the Supplementary Material section 1.1.

The original variant is the question mostly used in the three-item form of the CFT (Frederick, 2005). Both the trivial and the complementary variant allow to differentiate remembered answers to the original problem from answers to the posed problem. Asking for the price of the bat instead of the price of the ball does not change the structure and complexity of the problem. The correct solution (105 cents) is the complement of the original solution (5 cents). The potential intuitive solution (100 cents) is the complement of the former intuitive solution (10 cents). For the trivial variant, the intuitive response is the correct response as the solution is the simple difference *$*1.10−*$*1 = *$*0.10. A related variant was introduced by De Neys et al. (2013) in the form: “A magazine and a banana together cost $2.90. The magazine costs $2. How much does the banana cost?” (p. 270), which was solved by 98% of participants. Similarly, Raoelison and De Neys (2019) and Bago and De Neys (2019) included “no-conflict” problems with this structure, and Raoelison and De Neys (2019) reported solution rates of 99%.

The “transformed variant” is a variant with the original problem structure but changed monetary values. A transformed variant in this sense is already featured in Frederick (2005). The banana-and-bagel problem is described as an analogous problem with a higher requirement for computation: “A banana and a bagel cost 37 cents. The banana costs 13 cents more than the bagel. How much does the bagel cost?” (p. 28). In this case, 24 cents (37−13) is a more easily disqualified answer, and Frederick (2005) found respondents to perform much better on this transformed problem than on the original. The similar soup-and-salad problem in Finucane and Gullion (2010) was again solved by a higher number of participants (65%, see Table S3) than the original problem (29%), and did not generate frequent intuitive answers in Baron et al. (2015, p. 273, footnote 7).

Applying the structure-mapping model for word problems (Reed, 1987) to the variants used in this study, only the transformed variant can be considered isomorphic to the original problem. Both the trivial and the complementary variant share surface elements and a similar story with the original item but require different calculation procedures. Participants with previous exposure to the original item would be expected to be challenged by the merely similar items. The story similarity for transformed variant, on the other hand, should help to apply the correct procedure (Ross, 1989), unless the solution is merely memorized as a number.

### 2.3. Results

#### 2.3.1. Correct and False Answers

**Figure 1:**
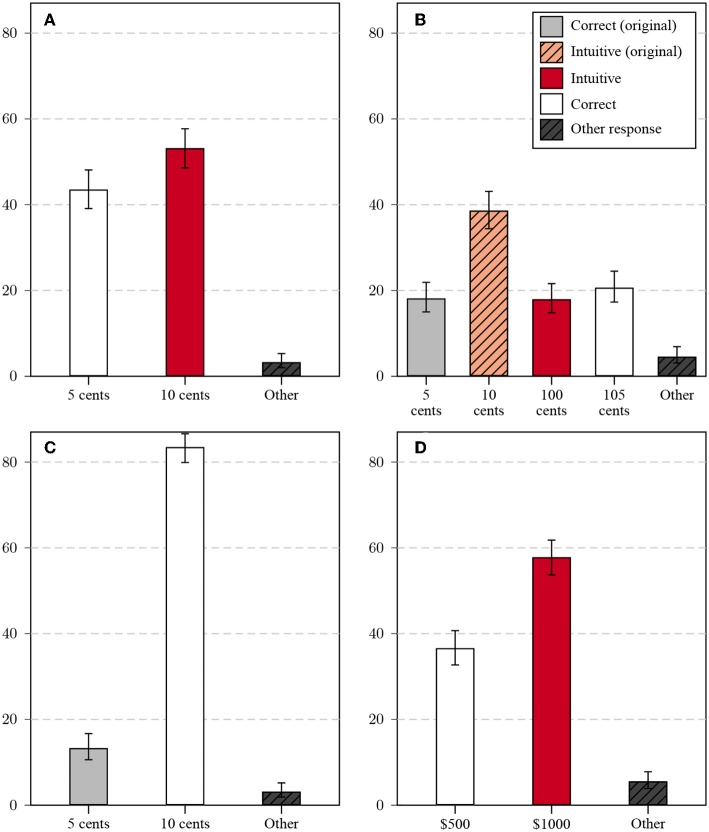
Relative frequencies of response categories for the four question variants in Study 1 [**(A)** original, **(B)** complementary, **(C)** trivial, and **(D)** transformed variant] in percent: All non-listed responses are categorized as Other, error bars mark the 95% CI of the proportion.

Answers to the standard CRT items are often categorized into three categories; namely (a) correct responses, (b) false, but intuitive responses, and (c) false, and non-intuitive responses. Baron et al. (2015) considered items to be lure-items, if one false and intuitive answer is both a frequent response and also the most frequent false response. In addition to these three categories, I formed separate categories for correct and false intuitive responses to the original problem, if those differed from correct and false intuitive answers to the problem variant, resulting in five response categories for the complementary variant and three categories for the trivial problem (in which the intuitive and correct category otherwise overlap). Results for the four variants are summarized in Figures 1A–D.

Responses to the standard version fell into the expected categories (see Figure 1A), with 43.5% correct answers (5 cents) and 53.2% false and intuitive answers (10 cents; only 3.3% of answers were different from 5 and 10 cents). This percentage is comparable to previous studies on MTurk, but the correct proportion was higher than the rate observed in earlier lab studies.

Answers to the complementary problem (see Figure 1B) fell into more than three major categories: The correct response ($1.05) was given by only 20.7% of participants, the false and intuitive response ($1.00) by 18% of participants. It should be noted in particular that the majority of participants gave an answer corresponding to the price of the ball, the focus of the original question. Of all answers, 18.2% gave the correct response to the original question (5 cents), and 38.6% the intuitive, false response to the original question (10 cents). Among the answers focusing on the wrong target object, the intuitive answers were more frequent than among the answers focusing on the correct target object.

In spite of the fact that the intuitive response to the trivial version (see Figure 1C) is indistinguishable from the correct response (10 cents), only 83.5% of the responses were correct. A substantial number of respondents (13.3%) responded with the solution to the original question (5 cents)^6^.

The transformed question (see Figure 1D) had the same answer categories as the original: The correct response ($500) was given by 36.6% of participants, the false intuitive response ($1,000) by 57.8% of participants. This high percentage indicates that the transformed item can still be considered a lure item, in contrast to previously used transformed items (Finucane and Gullion, 2010; Baron et al., 2015).

Comparing the response patterns across items allows for a preliminary interpretation. Answers to the original problem replicated the elevated rates of correct responses on MTurk relative to naive laboratory student participants. Answers to the complementary problem illustrate the effect of prior experience: The majority of respondents gave an answer expected for the original variant, although the complementary variant is merely similar, not equivalent to the original (Reed, 1987). This exemplifies the potential of surface similarities that are detached from structural similarities to impede performance (Ross, 1989; Lee et al., 2015) and to interfere with transfer (Morley et al., 2004).

It is unlikely that the responses were produced by simple errors, as $1.05 is an unlikely answer in the original task. Both the correct and the false answer are reproduced, albeit not at the same rate: The false answer to the original problem was much more likely to be reproduced than the correct answer. This validates the CRT scale on a meta-level: Participants who demonstrated higher cognitive reflection on the original test were able to realize the difference between expected task and presented task more easily. Further, many participants did not seem to read the task in detail. Responses to the trivial variant confirmed that some participants reproduced answers to the original question when facing a much simpler question, either due to a lapse of attentiveness or to the reproduction of a memorized response without insight.

This is consistent with comparisons with transformed variants in Chandler et al. (2014), who found similar solution rates (around 54%) for both^7^, and Meyer et al. (2018) (both around 40%).

Regarding threats to validity, it might be reassuring to see in answers to the complementary variant that false answers to the original problem seemed to be remembered at similar if not higher rates than correct answers. The accurate reproduction of previous answers certainly increases the reliability of a test. At the same time, this would also imply that the involved cognitive processes (reasoning vs. remembering) are dissimilar between first and subsequent exposure. The relationship between response types and both process and external variables should therefore sharpen the sketched interpretation.

#### 2.3.2. Response Times

**Figure 2:**
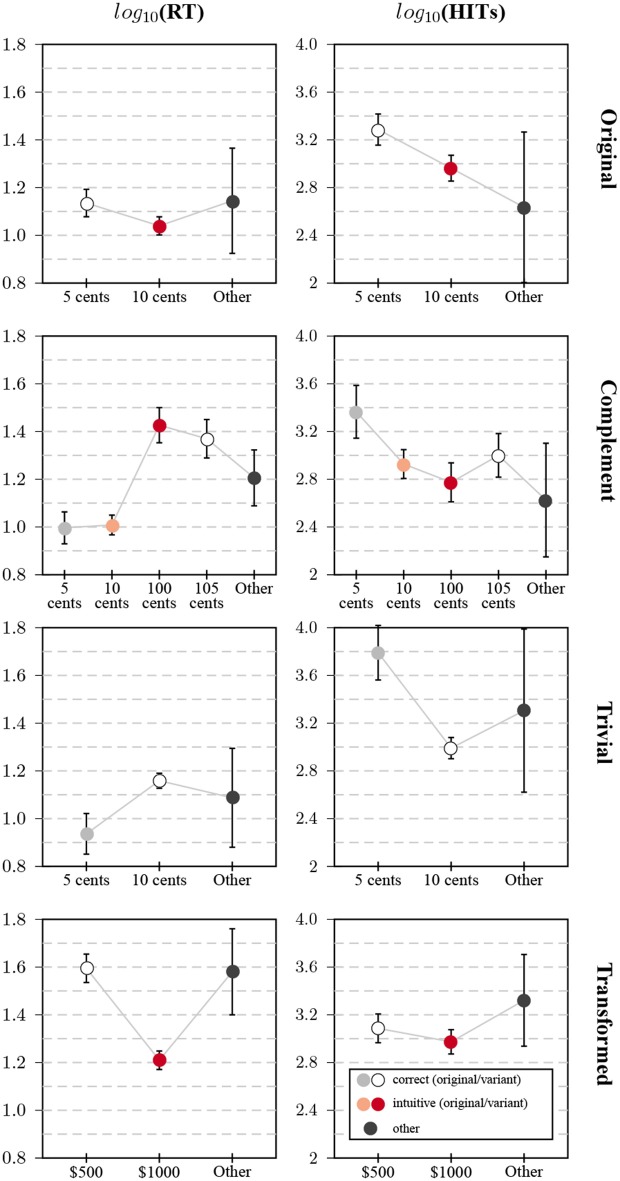
Relationship between response categories and response time/number of HITs in Study 1: Plots show average values of the log-transformed response time (left column) and the log-transformed number of previous HITs (right column). Each row contains the plots for one of the four task variants (from top to bottom: original, complementary, trivial, transformed); whiskers correspond to the 95% CI of the mean.

Item response times can help to identify items that may have been compromised by public exposure (Burke, 2009; Choe et al., 2018). The original distributions of response times were severely right-skewed, therefore all values were log-transformed before analysis (see the Supplementary Material section 2.1.1). Here, I compare differences in log-transformed response times between participants whose responses fell into the categories discussed above (see Figure 2, left column). For the original task, intuitive responses were given faster than correct responses, but the difference was relatively small (*d*_*c*−*i*_ = 0.1, 95% *CI* = [0.03, 0.16]). This is consistent with the idea that two groups of participants remembered and reproduced correct and incorrect responses, respectively, which diluted potential differences for naive participants.

Direct evidence for reproduction from memory was found for the complementary variant. Answers relating to the value of the ball were indeed given much faster than answers relating to the value of the bat, both for correct (*d*_*o*−*c*_ = 0.37, 95% *CI* = [0.27, 0.48]) and intuitive answers (*d*_*o*−*c*_ = 0.42, 95% *CI* = [0.33, 0.50]). There was little difference in response times between correct and false answers for the bat, the correct answer was even produced slightly faster (*d*_*c*−*f*_ = −0.06, 95% *CI* = [−0.17, 0.05]). It might well be the case that some participants produced the answer by subtracting the memorized answer from the total, with a speed advantage for respondents with higher CRT scores that predict higher numeracy (Cokely et al., 2012).

The improper original answer to the trivial problem was likewise given very fast, and faster than the correct answer (*d*_*o*−*c*_ = −0.22, 95% *CI* = [−0.31, −0.13]) consistent with a reproduction from memory. Response times for answers to the transformed problem showed the clearest evidence for the intuitive answer to be produced faster than the correct answer (*d*_*i*−*c*_ = −0.39, 95% *CI* = [−0.46, −0.31]). As this task was novel, a simple reproduction of answers from memory was not possible and response times were therefore also much slower than for the original item. Large differences were also observed in Chandler et al. (2014).

Based on this finding, one might speculate that most participants who solved the original problem were not simply producing a learned response (some might have, though) but generalized the original solution to a transformed task (presented for the first time on MTurk, to the best of my knowledge). On the other hand, a higher proportion of MTurk participants than typical lab participants has directly or indirectly learned how to solve the bat-and-ball problem, even when it is presented in a transformed version.

#### 2.3.3. MTurk Tenure

Participants' estimates of the number of prior HITs was interpreted as a measure of tenure and experience. These estimates showed a similarly right-skewed distribution as response times, and were likewise log-transformed before analysis^8^. As reported before (e.g., Haigh, 2016), participants who gave the correct answer to the original problem (results are shown in Figure 2, right column) were more experienced on average than participants who gave the intuitive, false answer (*d*_*c*−*i*_ = 0.32, 95% *CI* = [0.15, 0.49]).

Participants who erroneously gave the corresponding answers for the ball for the complementary variant were more experienced than those answering in regard to the bat. This difference was more pronounced for the two “reflective” answers ($0.05 vs. $1.05, *d*_*bl*−*bt*_ = 0.37, 95% *CI* = [0.08, 0.65]), than for the intuitive answers ($0.10 vs. $1.00, *d*_*bl*−*bt*_ = 0.15, 95% *CI* = [−0.05, 0.36]). The pattern for the transformed variant was similar to the pattern for the original, but with a smaller difference between the two answer categories (*d*_*c*−*i*_ = 0.11, 95% *CI* = [−0.04, 0.27])^9^. The most extreme difference was observed for the trivial problem: participants who responded with the original answer were far *more* experienced than those who responded with the correct answer (*d*_*o*−*c*_ = 0.8, 95% *CI* = [0.56, 1.04]).

Participants were also asked whether they had seen the same or a similar problem before, and the analysis aligned with the analysis for tenure (see Supplementary Material section 2.1.2). All results were consistent with answer memorization by a certain percentage of participants. The results found for Chandler et al. (2014) could be replicated: Participants who solved the original problem had a significantly higher number of HITs (log-transformed) than those who did not [*F*_(1,455)_ = 16.20, *p* < 0.001, partial η^2^ = 0.03]. This did not hold for the transformed problem [*F*_(1,555)_ = 1.04, *p* = 0.31, partial η^2^ = 0.002].

#### 2.3.4. General and Specific Experience

**Figure 3:**
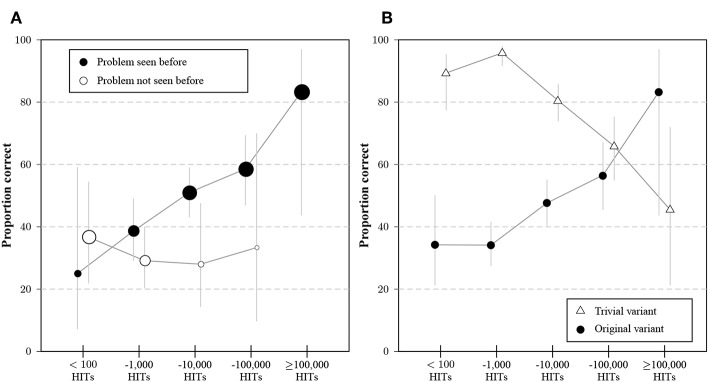
**(A)** Average proportion of correct answers to the original variant of the problem for participants that indicated experience (black dots) or no experience (with dots) with the bat-and-ball problem, separated by the category of previous HITs on MTurk. Dot areas correspond to the proportions of participants with and without experience for a given interval of HITs. Whiskers indicate 95% CIs for the proportions. **(B)** Percentage of correct responses to the original variant (circles) and the trivial variant (triangles) for participant groups whose stated number of previous HITs falls into different categories. Whiskers indicate 95% CIs for the proportions.

The simultaneous effects of general and specific experience are shown in Figure 3A. Participants who stated that they had not seen the problem before answered the question at a relatively constant rate, no matter how many HITs had been completed. For participants who indicated familiarity, there was a clear effect of tenure on MTurk, the proportion of correct responses increased from rates comparable to earlier studies with naive participants (Frederick, 2005) to an average value of 80% for the most experienced participants. Consistent with expectations, the proportion of inexperienced participants decreased with the number of HITs.

The inverse effect of experience on solving the trivial task is illustrated in Figure 3B by contrasting solution rates for the original and trivial problem. Experience on MTurk is positively related to solving the original task, but negatively to solving the trivial task. The transformed task showed a decreased dependence on platform tenure than the original task, but a similar structure regarding answer types. Some participants might have learned the principle that allowed them to solve the transformed task by being exposed to the original task.

#### 2.3.5. Comparison With Data From a Different Online Panel

**Figure 4:**
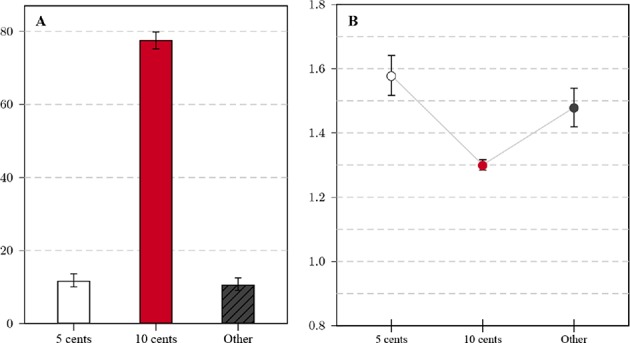
**(A)** Relative frequencies of response categories for the standard variant (Qualtrics data, *n* = 1, 238): All non-listed responses are categorized as Other, error bars mark the 95% CI of the proportion. **(B)** Average log-transformed response time for the standard variant (Qualtrics data); whiskers correspond to the 95% CI of the mean.

To establish whether the score improvement over time is unique to MTurk or a general phenomenon, I re-analyzed the CRT data (*n* = 1, 238) collected using Qualtrics Panels in 2018 in the context of Lewandowsky et al. (in preparation, see the Supplementary Material sections 1.2, 2.1.3 for details). Response patterns markedly differed from responses on MTurk. The percentage of correct solutions to the bat-and-ball question was clearly much lower (11.7%, see Figure 4A) and the percentage of false, intuitive answers correspondingly higher. As panel participants answered all three CRT items, test scores could be computed for this sample: The average score across the three items was 0.45, with 72.1% of the sample not solving a single item correctly and only 4.8% solving all.

Response times for the online panel (see Figure 4B) were generally longer on average than for the MTurk sample. The difference in log-transformed times between correct and intuitive answers (*d*_*c*−*i*_ = 0.28, 95% *CI* = [0.23, 0.33]) is similarly pronounced as for the transformed variant and more pronounced than for the standard variant on MTurk. The Supplementary Material section 2.1.4 features further analyses of differences between successful and unsuccessful participants in relation to gender and household income.

### 2.4. Discussion

Going back to the research questions that motivated Study 1, some responses can be offered based on the observed results. Regarding Research Question 1, most participants' responses were categorized as intuitive, but the proportion of correct responses was higher than in older studies (and consistent with more recent studies). Further, response time differences between correct and intuitive responses were relatively small, which is somewhat inconsistent with the theoretical foundation for these categories.

The analysis of item variants (Research Question 2) resulted in two major findings: First, subtle variations were often overlooked, resulting in answers expected for the original item. In the case of the complementary variant, the majority of responses evidently assumed the text of the original item. This clearly demonstrates that memorization plays a role in these responses. This assumption is bolstered by the analyis of response times, demonstrating that responses linked to the original item are given much faster than responses expected for the variant: many participants seemed to have relied on answers stored in memory. Second, a more obvious variation of the original item (the transformed variant) resulted in clear differences in response patterns: The correct response was given at a reduced rate and clear response time differences emerged between correct and intuitive responses as predicted by the theory.

The analysis of panel tenure and previous exposure to the bat-and-ball problem provided some answers to Research Question 3: Panel tenure had a substantial (positive) impact on the proportion of correct responses. This effect was exclusive to participants who reported seeing the item before (which in itself exhibited a strong correlation with panel tenure). The pattern for item confusion showed the opposite trend: experienced participants were much more likely to overlook subtle variations of the original item. Finally, for the transformed variant, tenure had a reduced impact on correct responses.

Regarding Research Question 4, data collected recently from Qualtrics participants gives a strong indication that exposure to the CRT and the subsequent increase in scores observed on MTurk is not a general phenomenon created by media coverage or teaching efforts.

## 3. Study 2: Comparing the Original and a Transformed CRT

The observed effect of panel tenure on solution rates is a practice effect, based on the results of Study 1. Errors observed for the trivial and complementary variants showed that improvements were partially due to mindless memorization. Yet, mere memorization could not explain the relatively high rate of correct responses to the transformed variant. This suggested that a parallel version of the CRT based on transformed items could be a reasonable alternative selectively targeting mindless memorization without punishing those familiar with the original. Study 2 compared the original CRT with a transformed variant, regarding response process and validity in an MTurk sample.

### 3.1. Research Questions

Study 2 aimed to address two research questions. Confronting participants with two variants of the CRT allowed me to address Research Question 5:

Research Question 5. *How does a transformed variant of the CRT relate to the original?*

Beyond the direct comparison of responses to the two sets of items, Research Question 6 was aimed to compare the relationships of the two scales with other variables, such as panel tenure and measures of constructs that were expected to correlate with the CRT (and therefore also with its variant):

Research Question 6. *What is the relationship between the two scales, panel tenure and measures of related concepts?*

Specifically, measures of financial literacy and subjective numeracy were collected for all participants in Study 2.

### 3.2. Methods

#### 3.2.1. Sample

The study was conducted within a longer sequence of tasks on MTurk in August 2018. The HITs were announced to last 20–30 min for most participants and offered a fixed payment of $3.00 with a bonus of up to $1.00. Participation was restricted to US participants with a minimum of 98% acceptance rating and 101 completed HITs. At the time of the study, data quality concerns on MTurk were voiced by multiple researchers. Consistent with the account in Kennedy et al. (2018), location data revealed a relatively high number of attempts from Venezuela (in spite of the location filter). These attempts were automatically blocked and subsequent attempts that used the same MTurk IDs (spoofing a US location) were not excluded. Of 1,109 survey attempts, only 918 sessions reached the introductory demographics section after passing the attention checks and 729 participants completed the survey. A careful analysis of double participation, IP address clusters, and non-US participants led to a final sample size of *n*_3_ = 700. Participants were on average 36.4 years old (*SD* = 10.5 years), and 49.1% categorized themselves as female (50.6% as male, 2 participants chose neither category). The median number of previously completed HITs was 5528.5 (*M* = 40, 554, *SD* = 172, 488.5 HITs). Participants reported medians of 14 months and 19 weekly hours working on MTurk.

#### 3.2.2. Survey Questions

The transformation used in Study 1 left the structure of the task intact and only shifted the numbers (and added the attribute “golden”). It is possible to generate a potentially unlimited number of item “clones” (Glas and van der Linden, 2003; Arendasy and Sommer, 2013; Lathrop and Cheng, 2017) with different solutions but similar difficulty by changing an item's incidental but not its radical elements (Irvine, 2002). The Supplementary Material section 3 provides such item models (Arendasy and Sommer, 2012) for the three original CRT items. Knowing the original solution should convey an advantage in solving transformed items, as long as this knowledge is based on problem insight and not mindless memorization. In Study 2, the original CRT was compared with a transformed variant (CRTt) in a within-subject design. The three transformed variants (the first is taken from Study 1) that comprise the CRTt are presented in Table 1.

All CRT items appeared before the first CRTt item. Further, two additional scale measures were selected as correlates for validation based on previous findings for the CRT, namely subjective numeracy (Fagerlin et al., 2007) and financial literacy (Hastings et al., 2013). All items are listed in the Supplementary Material section 1.3.

### 3.3. Results

#### 3.3.1. Average Score and Inter-correlation

The mean CRT score (*M* = 1.93, *SD* = 1.19) was higher than the mean CRTt score [*M* = 1.60, *SD* = 1.13, *t*_(699)_ = 13.05, *p* < 0.001, *d*_*rm*_ = 28^10^, two-sided paired samples *t*-test]. Note that possible practice effects within the task would advantage the CRTt. Both scores are substantially higher than the observed mean score for the Qualtrics sample (*M* = 0.45) and still higher than average scores reported for the CRT in Frederick (2005). Further, CRT and CRTt scores were highly correlated (*r* = 0.84, *N* = 700, *p* < 0.001), mainly due to large groups of participants with minimum and maximum scores for both tests (see Supplementary Material section 2.2.2). The high correlation was a positive indicator for both the reliability of the original CRT and the internal validity of the CRTt. It is also consistent with the level of retest-reliability found in Stagnaro et al. (2018, *r* = 0.81).

#### 3.3.2. MTurk Tenure

**Figure 5:**
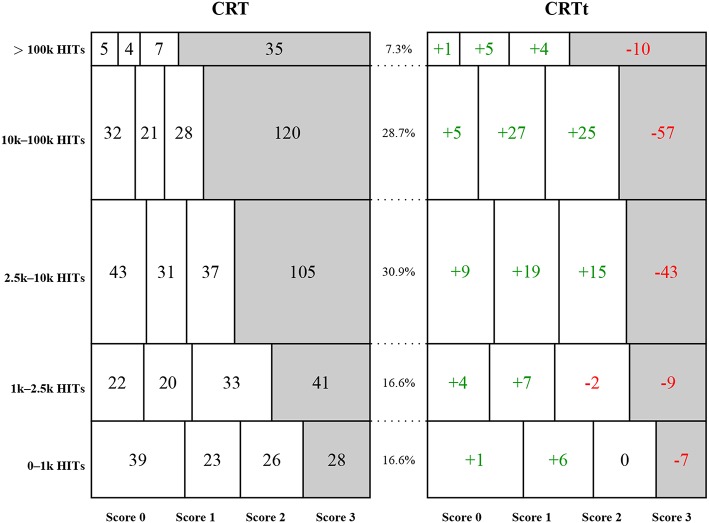
Absolute frequencies of CRT scores and deviations for CRTt scores split by categories of self-reported number of completed HITs in Study 2: Each row reports on the group of participants whose number of reported HITs falls into the specified interval. The left mosaic plot shows absolute numbers of the four possible scores, the numbers on the right side show differences for the CRTt frequencies, with positive numbers indicating a larger frequency for the CRTt. Each rectangle is proportional in size to the observed frequency of the combination of score and participant group. Relative frequencies of the five categories are reported in the middle column.

Comparing groups with different amounts of previous MTurk experience revealed a systematic difference between the score distributions for the two tests. Figure 5 illustrates the distribution of test scores split by panel tenure. Differences between test scores were more pronounced for experienced participants: While the CRT scores exhibited a distinct ceiling effect for participants with 2,500 HITs and higher, this effect was much less pronounced for the CRTt, the four score categories were more evenly distributed for groups with up to 100,000 HITs. Consistent with this observation, the correlation between the score on the original CRT and tenure (the logarithm of the number of HITs) was higher (*r*_*o*_ = 0.25, *p* < 0.001, *N* = 700) than the correlation between the score on the CRTt and tenure (*r*_*t*_ = 0.17, *p* < 0.001, *N* = 700). The difference between these dependent correlations was significant (*Z*_*H*_ = 3.49, *p* < 0.001, Steiger's *Z*, two-sided test for equality; Hoerger, 2013).

**Figure 6:**
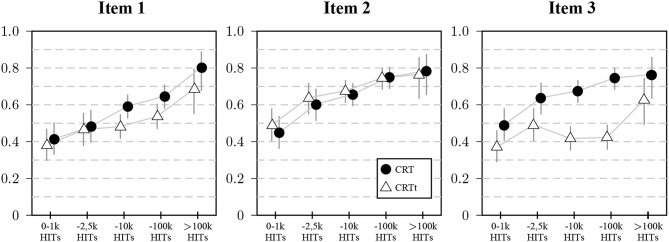
Proportion of participants giving correct answers to the three items in Study 2 split by panel tenure: Markers represent proportions of correct answers split by self-reported number of HITs, vertical lines represent 95% CIs for the proportions.

The individual item analyses—shown in Figure 6—revealed that for all three items, scores increased with panel tenure to a significant degree, mainly driven by the first and third item. On the first item, 11 participants had a better result for the CRTt, 69 participants for the CRT. For the second item, 22 had a better score on the CRTt, 11 a worse score. For the third item, only 6 had a better score on the CRTt, 189 had a worse score.

#### 3.3.3. Scale Version and Response Types

**Figure 7:**
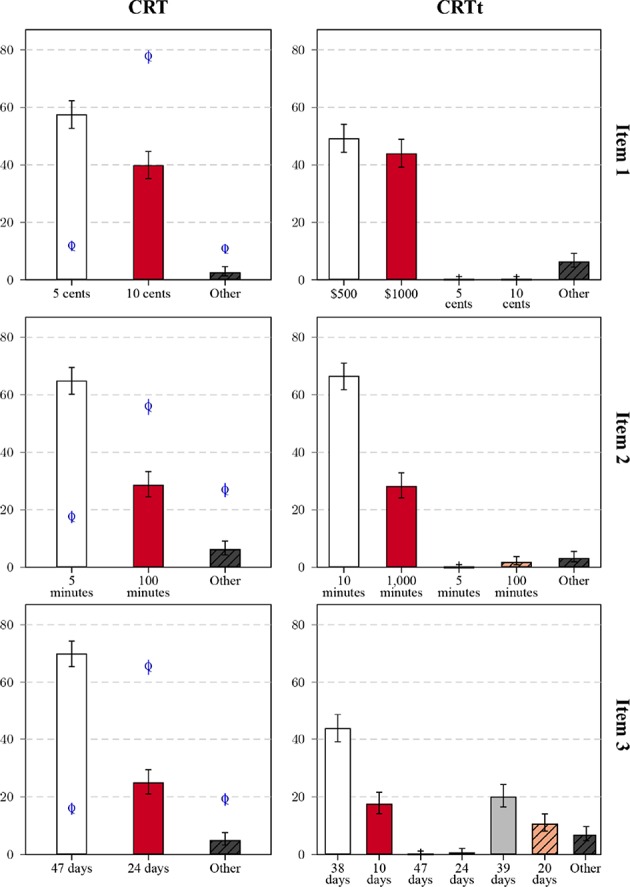
Relative frequencies of response categories for the three items (rows) in the original version (CRT, left columns) and transformed variant (CRTt, right column) in Study 2: All non-listed responses are categorized as Other, error bars mark the 95% CI of the proportion. Bars correspond to the results in the MTurk sample, for the CRT items, results for the Qualtrics sample are marked by the letter Q with CI bars.

Figure 7 compares the distribution of frequent responses to the three question pairs, and adds the results obtained for the Qualtrics sample as reference points. For item 1, the bat-and-ball problems, both variants were answered correctly at a much higher proportion than the original problem on Qualtrics, which was true for all three items. Participants on Qualtrics also differed in their more frequent production of unique responses. Between the variants, the observed worse performance for the transformed version (about 8%) was due to a similar increase in both intuitive responses and infrequent other responses. Responses linked to the original question were rarely observed. There were similar response distributions for item 2, again with little confusion between the items. Differences between the variants were more pronounced for item 3. The lower performance for the transformed variant was due to some interference between variants: One group of participants gave the correct solution, another group the false, intuitive answer to the original question. Both groups together comprised nearly a third of the sample. Response times (see the Supplementary Material section 2.2.1 for a graphical summary) showed a similar pattern as in Study1: Correct solutions were associated with *shorter* average times for the original items on MTurk and the expected longer response times for CRTt items (and original items in the Qualtrics data).

#### 3.3.4. Correlations With Other Measures and Gender Effects

The reduction in ceiling effect demonstrated for the CRTt compared to the CRT would allow, in principle, for a better differentiation between experienced participants and a better predictive performance— if the finer differentiation was indeed related to the measured construct. To test this, I consider one categorical variable (gender) and two continuous cognitive measures (subjective numeracy and financial literacy) that have been shown to be related with the original CRT (Frederick, 2005; Campitelli and Labollita, 2010; Cokely et al., 2012; Bialek and Pennycook, 2017).

##### 3.3.4.1. Gender

Gender information was collected for both the second MTurk sample and the Qualtrics sample. A repeated-measures ANOVA for the MTurk data with scale version as within-factor and gender and HIT number category as between-factors, showed a significant (ordinal) interaction of version and gender [*F*_(1,688)_ = 4.03, *p* = 0.04, partial η^2^ = 0.006] and significant main effects for version [*F*_(1,688)_ = 117.51, *p* < 0.001, partial η^2^ = 0.15] and gender [*F*_(1,688)_ = 6.89, *p* = 0.009, partial η^2^ = 0.01]. Table 2 presents means for female and male participants across the variants and samples and illustrates both effects: Male participants have higher scores than female participants for both tests, while this difference is larger for the CRTt than for the CRT^11^. In addition, there was a main effect for HIT number category [*F*_(4,688)_ = 9.62, *p* < 0.001, partial η^2^ = 0.05]—the practice effect—and an interaction between version and HIT number [*F*_(4,688)_ = 5.90, *p* < 0.001, partial η^2^ = 0.03], as illustrated in Figure 5. Table 2 also reports the corresponding results for the Qualtrics sample (with participants likely to have had less exposure to the CRT, as reflected in the lower mean scores). The CRT score difference was larger in the Qualtrics than in the MTurk sample, and similar to the CRTt difference, with all scores lower in the Qualtrics sample. Note that gender was chosen because of demonstrated robust differences for correct and false solutions in the past (Frederick, 2005; Campitelli and Labollita, 2010; Campitelli and Gerrans, 2014; Toplak et al., 2014; Cueva et al., 2016), not because of substantive theory linking gender and expected solution rates.

**Table 2:** Mean scale scores for the CRT (Qualtrics and MTurk) and the CRTt (MTurk) split by gender and test for differences (two-sided independent samples *t*-test; Mturk: *n*_*female*_ = 344, *n*_*male*_ = 354, Qualtrics: *n*_*female*_ = 692, *n*_*male*_ = 546), 95% CI for the difference in group means and Cohen's *d*.

**Scale**	**Sample**	***M*_*female*_**	***M*_*male*_**	**Δ_*M*_**	***t***	***p***	**95%CI Δ_*M*_**	***d***
CRT	MTurk	1.78	2.07	0.29	3.26	0.001	[0.12, 0.47]	−0.25
		(1.21)	(1.15)					
CRTt	MTurk	1.40	1.77	0.37	4.34	<0.001	[0.20, 0.53]	−0.33
		(1.11)	(1.12)					
CRT	Qualtrics	0.28	0.66	0.38	8.23	<0.001	[0.29, 0.47]	−0.46
		(0.69)	(0.93)					

*Standard deviations are presented in brackets below the means*.

##### 3.3.4.2. Subjective numeracy and financial literacy

Participants obtained a mean score of 13.7 (*Md* = 14, *SD* = 3.09) on the subjective numeracy scale and a mean score of 3.7 (*Md* = 4, *SD* = 1.16) on the financial literacy measure. For both subjective numeracy and financial literacy, correlations with the CRTt were higher than those with the CRT (see Table 3). Again, these results can be interpreted as ambivalent news for the CRT: On the one hand, correlations with related variables prevailed in spite of familiarity and repeated exposure. On the other hand, average scores seem to have increased beyond an optimal point, such that a ceiling effect hurts differentiation (the earlier floor effect is certainly no longer a concern). Thus, at least for the observed sample, the transformation of items increased correlations.

**Table 3:** Pearson correlations and Steiger's Z for original CRT (*o*) and new CRTt (*t*) with subjective numeracy (Fagerlin et al., 2007) and financial literacy (Hastings et al., 2013) in Study 2.

	***r*_*o*_**	***r*_*t*_**	***Z*_*H*_**
Subjective numeracy	0.24	0.30	−2.68
*p*	<0.001	<0.001	0.007
Financial literacy	0.34	0.38	−1.95
*p*	<0.001	<0.001	0.051

*The p-value for Z_H_ is the result of the two-sided test for equality of dependent correlations (Hoerger, 2013)*.

In conclusion, the proposed transformed variant was shown to be promising for the use on MTurk. While the test might require participants to spend more time, on average, correlations with external measures were higher than for the original. Further, only for the novel variant did response times differences correspond to the assumed cognitive process. One might object that the observed differences were connected to the ability to deal with numerical information, as the CRT has been criticized for its dependence on numeracy (e.g., Thomson and Oppenheimer, 2016), so further research might be warranted. The general construction principles applied (listed in the Supplementary Material section 3) allow for the generation of many more variants, which could potentially extend the viability of the test for a long time.

#### 3.3.5. Submission Comments

When submitting the HIT in Study 1, three participants who had responded to the trivial variant alerted us to the discrepancy with the standard version (e.g., “I think it's supposed to say the bat costs $1 MORE than the ball”). Part of the sample of participants were asked (before the CRT questions) to name tasks that they had encountered often or tasks that they saw as being used too often to be valid any more. Of 360 participants, 240 gave a response to this question. Among these open answers, 16 (7%) explicitly named CRT questions as tasks the respondents had encountered frequently, this was a more frequent response than the trolley problem (the most frequent response with 17% concerned variants of the dictator game). Some participants' answer showed direct evidence of memorizing responses, sometimes without insight. These anecdotal incidents were more systematically investigated in Study 3.

### 3.4. Discussion

To address Research Question 5, a number of main findings in Study 2 need to be jointly considered. A high correlation between original CRT and transformed CRTt can be considered as good news for the validity (also for the reliability) of the original CRT. If a substantial proportion of participant had learned correct responses simply by memorizing correct answers, this would have resulted in larger differences between the two measures. A closer analysis of responses to original and transformed variants nonetheless demonstrated that there were more participants giving correct answers to the original and false answers to the transformed items than participants with the opposite pattern. The analysis of process variables showed larger differences between the two scales than the analysis of response categories.

Regarding Research Question 6, Study 2 yielded relevant results both regarding panel tenure and validation variables. Both test variants showed a strong influence of panel tenure on solution rates, but this relationship was more pronounced for the original CRT. This resulted in a substantial ceiling effect for experienced participants for the CRT that was considerably reduced for the CRTt. At the same time, both scales still exhibit a floor effect for inexperienced participants. The difference between scales arguably resulted in differential correlations with other measures, with the CRTt showing higher correlations than the CRT for financial literacy^12^ and subjective numeracy.

## 4. Study 3: Novel Items and Sources of Memory

Study 3 explored MTurk participants' sources for memorized answers and the degree of compromised test items. In addition, three novel items were tested to determine whether the acquired resistance of MTurk participants to lure items generalized to unfamiliar problem types.

### 4.1. Research Questions

Finally, Study 3 investigated sources of item familiarity and measured performance on novel lure items.

Study 3 introduced two new elements: It featured novel items that were clearly unrelated to the original CRT items but of a similar problem type, and it included questions aimed at finding out more about sources and types of answer memorization by participants. The analysis of responses to the novel items allowed to address Research Question 7.

Research Question 7. *Is it possible to construct novel lure items that work on MTurk?*

Research Question 8 again extended the perspective to predictive validity and the comparison between old and novel items regaring the relationship with other variables:

Research Question 8. *Are answers to novel items influenced by panel tenure, and are they similarly predictive as the original items?*

The introduction of questions about memorization allowed to address Research Question 9:

Research Question 9. *How did participants learn and memorize responses to the bat-and-ball item?*

Specifically, I was interested in finding out whether participants remembered responses or procedures. Study 3 was complemented by open-format questions about participants' attitudes toward the CRT. Answers are briefly summarized below, and reported in more detail in the Supplementary Material section 2.4.

### 4.2. Methods

#### 4.2.1. Sample

The study was conducted on MTurk as part of a larger HIT in early 2019. The HIT was announced to last 12–15 min for most participants and offered a fixed payment of $1.50 with a bonus of up to $0.20. Participation was restricted to US participants, with a minimum of 96% accepted HITs, but no requirement of minimum number of HITs. Participants had to pass a screening for VPN-, VPS- or proxy use via iphub.info (Burleigh et al., 2018) and two out of three checks for attention, language comprehension and nationality. A total of 1,341 participants started the test, 1,066 passed the initial screening, and 1030 finished the test. Of these, nine were part of the pilot study, and I excluded ten participants due to reasonable doubts about their location or double IP addresses^13^, resulting in a final sample size of *n*_4_ = 1, 011. Participants were on average 36.6 years old (*SD* = 11.9 years), and 46.8% categorized themselves as female (53.1% as male, one participant chose neither category). The median number of previously completed HITs was 2,950 (*M* = 21, 668, *SD* = 103, 230.5).

#### 4.2.2. Survey Questions

##### 4.2.2.1. Item variants

Most participants answered five questions that were either original CRT-items or variants. Here, I analyze the two original items and two novel items listed in Table 1 that were presented after two variants of CRT-items. The full list of items is presented and analyzed in the Supplementary Material section 2.3. The two novel variants were designed to be lure items.

##### 4.2.2.2. Solution sources and strategies

After the bat-and ball question, participants were asked whether they had encountered the task before. Depending on the answer to this question, the survey included one of two sets of follow-up questions. Participants who had encountered the item before were asked how often and where they had encountered the item before, whether they had memorized solutions or strategies, and whether they had ever received feedback on their responses or incentives for correct answers (see Supplementary Material section 1.4 for all questions). Participants who had not encountered the item before were asked whether they solved the task on their own or searched for solutions. Both groups were asked—in an open response format—about their opinion about the item and its use on MTurk.

##### 4.2.2.3. EV-scale

Participants made three choices between gambles, for which participants scoring high on the CRT chose EV-maximizing options in contrast to low-scoring participants in Frederick (2005). The number of EV-maximizing choices was counted as a simple score between 0 and 3.

##### 4.2.2.4. Attention checks

Due to the requirement to pass at least two out of three attention checks before the survey, participants in Study 3 had made either one or zero errors. As CRT items and attention checks share the element of intuitive, yet false responses, attention check performance has been related to CRT results (Hauser and Schwarz, 2015). Of the participants that entered the study, 126 (12.5%) committed one error across the three items.

### 4.3. Results

#### 4.3.1. Original Items and MTurk Tenure

**Figure 8:**
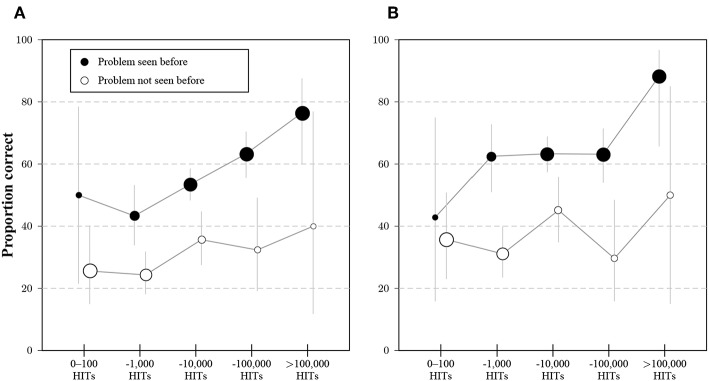
Average proportion of correct answers to item 1 **(A)** and item 2 **(B)** for participants in Study 3 that indicated exposure (black dots) or no exposure (white dots) to the bat-and-ball problem, separated by the category of number of previous HITs on MTurk. Dot sizes correspond to the proportions of participants with and without experience for a given category of HITs. Whiskers indicate 95% CI for the proportions.

The relationship between panel tenure and solution rates for the original two CRT items are presented in Figure 8. A comparison of Figure 3A with Figure 8A shows that the practice effect found in Study 1 for the bat-and-ball problem was replicated in Study 3, and a similar effect was found for item 2.

#### 4.3.2. Novel Items

**Figure 9:**
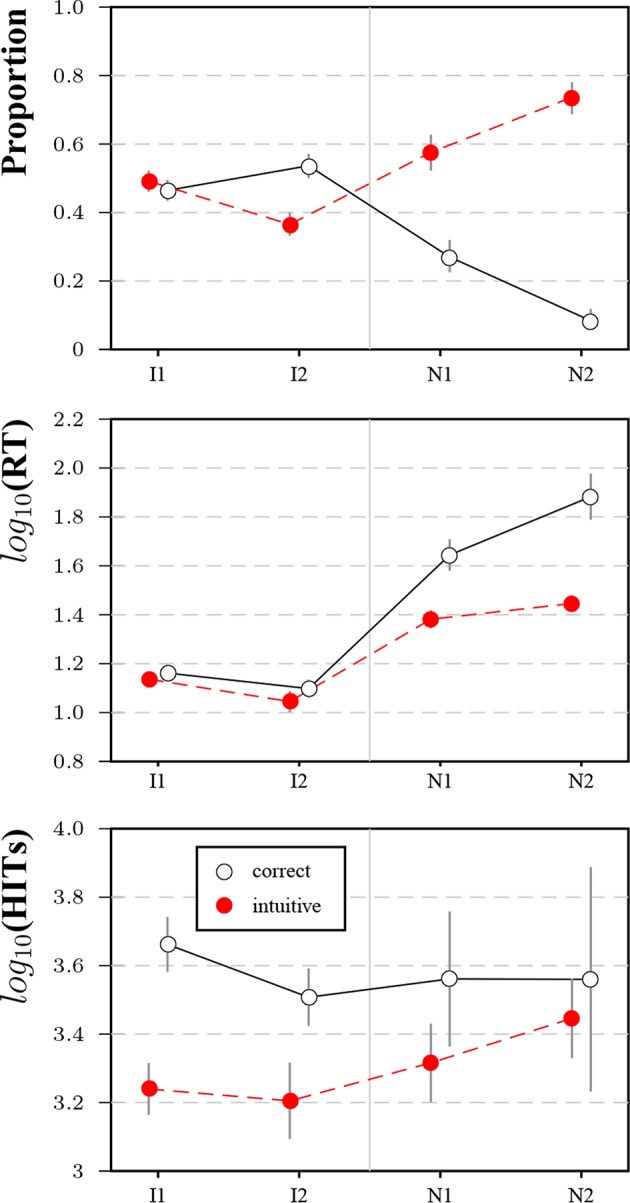
Results for item 1 (I1), item 2 (I2) and the two novel items (N1 and N2) in Study 3. Subfigures show the proportion of intuitive and correct responses (top row), average logarithmized response times for response types (second row), and average logarithmized number of HITs (bottom row). Bars represent 95%-CIs for proportions and means, respectively.

Figure 9 shows the results for the two novel items (and the original items for benchmarking). Response frequencies demonstrate that N1 and N2 both elicited a much higher rate of intuitive than correct responses. Both novel items took participants much longer to answer irrespective of answer type, with correct answers taking the longest. The gaps in panel tenure for the original items were less pronounced for N1 and N2. These results suggest that the MTurk population has not been immunized with respect to lure items, as there was no transfer to novel puzzle items, even though the novel items were presented after several blocks of other lure items.

**Figure 10:**
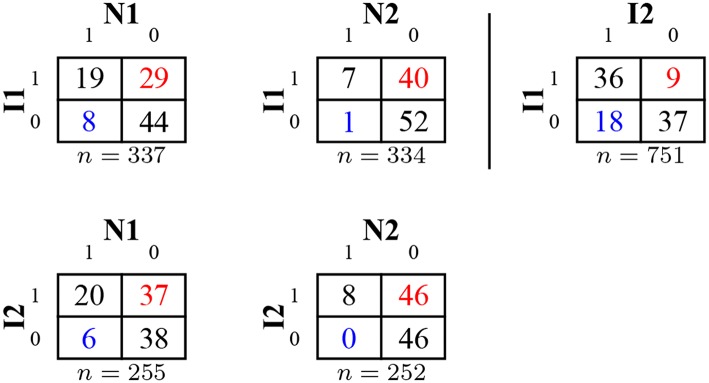
Cross-tabulation of correct and false responses for original and novel items (showing rounded percentages) in Study 3. Improvements from row item to column item are captured in the lower left corner (in blue), worse performance in the upper right corner (in red).

Figure 10 shows cross-tabulations for solutions to original and novel items. A worse performance for variants and novel items was much more frequent than a better performance. For all analyzed pairs, the majority of participants scored the same on both items (see also the Supplementary Material section 2.3.5).

#### 4.3.3. Validation Measures

Four measures were considered as validation measures for the CRT items and variants. I compare respondents with correct and false solutions in terms of gender and attention check errors, and in terms of the EV-scale and CRT-solutions.

Table 4 presents proportions and differences in proportions for respondents with correct and false solutions for each item in Study 3. With respect to gender, the difference for the original items was replicated, at a similar level as observed in Study 1 for I1 (see Supplementary Material section 2.1.4) and in a meta-analysis (Brañas-Garza et al., 2015). The largest differences were seen for N2. A difference in attention check errors was pronounced for the first, but not the second original item.

**Table 4:** Relative number of male participants and attention check errors: Proportions, differences in proportion and CIs for differences in proportions split by correct and false solutions for the items in Study 3.

	**Gender**				**AC error**			
	***p*_*inc*_**	***p*_*cor*_**	**Δ**	**CI Δ**	***p*_*inc*_**	***p*_*cor*_**	**Δ**	**CI Δ**
I1	47.1%	60.5%	13.4%	[7.2%, 19.4%]	15.6%	8.4%	−7.3%	[−11.2%, −3.2%]
I2	41.3%	60.9%	19.6%	[12.5%, 26.5%]	14.0%	9.7%	−4.4%	[−9.1%, 0.2%]
N1	48.8%	56.0%	7.3%	[−4.7%, 18.8%]	11.0%	8.8%	−2.2%	[−8.4%, 6.1%]
N2	52.0%	78.6%	26.6%	[−5.8%, 44.4%]	14.4%	14.3%	−0.1%	[−9.7%, 17.5%]

Table 5 presents means and mean differences in EV-scale and CRT-score for respondents with correct and false solutions for each item in Study 3. With respect to the EV-scale, both original items showed a difference in the expected direction. All items showed differences in the same direction as the original items. With respect to the CRT-score, the large difference for the original items was unsurprising, as a minimum difference of 1 was guaranteed. The observed differences for the novel items were less obvious and can be regarded as an additional confirmation that CRT items have not lost their validity.

**Table 5:** EV-scale scores and performance on original items: Means, mean differences and CIs for the mean difference split by correct and false solutions for the items in Study 3.

	**EV-scale**				**CRT (1+2)**			
	***M*_*inc*_**	***M*_*cor*_**	**Δ**	**CI Δ**	***M*_*inc*_**	***M*_*cor*_**	**Δ**	**CI Δ**
I1	1.49	1.97	0.48	[0.36, 0.59]	*0.33*	*1.79*	*1.47*	*[1.40, 1.53]*
I2	1.51	1.88	0.37	[0.23, 0.50]	*0.20*	*1.67*	*1.46*	*[1.40, 1.53]*
N1	1.62	1.79	0.17	[−0.06, 0.41]	0.88	1.51	0.62	[0.40, 0.84]
N2	1.71	2.00	0.29	[−0.06, 0.64]	0.92	1.86	0.95	[0.72, 1.18]

*Note that correct or incorrect answers to I1 and I2 limit the possible range of values for CRT (1+2), affected cells show values in italics*.

These results are consistent with the finding that repeated exposure to the CRT does not indiscriminately inflate scores and add measurement error, as the two novel items allowed for a relevant comparison: Both in terms of attention check errors and the EV-scale, the original item allowed for a better differentiation than the novel items.

#### 4.3.4. Sources of Familiarity

##### 4.3.4.1. Prevalence and type of previous exposure

A total of 32 participants indicated that they misclicked or that the only time they had seen the question before was in the HIT itself (or mistook item variants for the same item). After correcting for these, 658 participants (65.6%) were categorized as having seen the item before, 345 participants (34.4%) as not having seen the HIT before. A large majority of participants who had encountered the bat-and-ball problem before encountered it on MTurk (93.7%). The second most frequent category (lecture/class/presentation) was chosen by only 6.9% of participants. Printed sources (2.0%) and internet forums (2.0%) were named even less frequently. Few were able to name the exact source. There were isolated references to videos on sharing platforms or social media posts. These answers therefore ran counter to the proposition that mostparticipants underwent preparation for the CRT through MTurk-related websites and public communication of the test's solutions.

##### 4.3.4.2. Feedback and bonus money

Of those with previous exposure to the item, only 35 (5.3%) affirmed to have received feedback on any of their previous attempts (20 were given only correct/false information, 15 received the correct solution). Still, more participants in this group (85.7%) solved the item correctly than in the rest of the pre-exposure group (52.6%). On the other hand, 51 participants (7.8%) were offered money for giving the correct solution (36 of them did not receive feedback, though). A higher proportion of participants who had received bonus money solved the item correctly (74.5%) than of participants who had not received bonus money (54.0%). Both results confirm that both incentives and feedback can increase practice effects (e.g., Steger et al., 2018), but that they are encountered rarely in CRT studies.

##### 4.3.4.3. Memorization

Most participants with previous exposure to the bat-and-ball item claimed to have memorized either the answer (27.5%) or the calculation procedure (62.7%). The first group solved the item correctly at the highest rate (63.0%) followed by those with memorized procedure (56.6%). Participants without memorization only reached a solution rate of 28.1%. While the solution rates in the first two groups are high, the failure rate is still substantial. This is—again—an indication that not only correct answers and procedures are memorized, but also incorrect ones: The answers in the three groups fall into the intuitive category (10 cents) at relative frequencies of 32.6, 41.3, and 60.9%, respectively. Thus, subtracting 1$ from 1.10$ is regarded as the correct procedure by the second group (the Supplementary Material section 2.3.7 contains further analyses of the relationship between memorization strategies and performance).

The admitted memorization is consistent with observed response time differences [*F*_(2,654)_ = 7.14, *p* < 0.001, partial η^2^ = 0.02]. The fastest average logarithmized response times were observed for those with memorized answers (*M* = 1.05, 95% *CI* = [1.00, 1.10]), followed by those with memorized procedure (*M* = 1.12, 95% *CI* = [1.09, 1.15]) and those without memorization (*M* = 1.23, 95% *CI* = [1.13, 1.32]). Of those encountering the question for the first time, all but one participant claimed to have come up with the answer on their own. Nobody claimed to have searched for the answer online.

#### 4.3.5. Open Answers

A research assistant coded open answers into several non-exclusive categories (see Supplementary Material section 2.4 for the full list, examples, and the coding scheme). The answers shed some additional light on reasons for resistance to learning effects: about one in three participants without problem exposure and one participant in five with exposure interpreted the question as very simple. The proportion of correct answers in this group was below average. One theory—that those with prior exposure explicitly endorsed—interpreted the question as an attention check (6.5% of answers in this group) or a test (5.4%). Again, most of these participants answered incorrectly. Some participants suspected a trick (13.4% for first time exposure, 5.8% for repeated exposure), but also answered mostly incorrectly. Those who expressed liking the problem (7.0%/12.5%) had higher solution rates (50%/73.3%) than those expressing dislike (5.1%/6.4%; with solution rates of 18.8% and 52.3%, respectively). Few people (7.3%) in the repeated-exposure group declared the item “overused” (with a 70.0% solution rate) or found no challenge in it (4.1% with a solution rate of 96.4%). One in ten participants spontaneously named other CRT items (70% of these solved the item).

### 4.4. Discussion

Results for response categories and process variables confirm that it is still possible to construct novel lure items on MTurk, which answers Research Question 7 in the affirmative. Both the differences in proportions between correct and intuitive answers and the differences in response times are more pronounced for novel items than for original items.

Regarding Research Question 8, correct responses to novel items are given by more experienced participants, but the effect is weaker than for the original items. A plausible explanation would be some generalization in learning the responses to the original items or, at least, generalized skepticism toward seemingly easy questions. Results for validation measures and relationships with other variables are somewhat mixed, but taken as a whole would rather speak in favor of the original items: Differences between respondents with correct and incorrect answers in gender proportions, attention check errors, and the EV-scales are more pronounced for the original than for the novel items. Solutions to novel items predict solutions to the original items. A plausible explanation for this difference might be the comparatively higher difficulty of the novel items. Further, it is not entirely clear out of which larger set the original items might have been selected.

Finally, Research Question 9 is directly addressed by participants' responses to the memorization question. Most participants (about two in three) admitted to having seen the problem before. In this group, about nine in ten participants indicated that they had memorized either the answer or the calculation procedure for the bat-and-ball problem (most had memorized the procedure). At the same time, memorized answers and procedures were not necessarily the correct responses. One in three participants who had memorized a response had memorized the intuitive response and 40% of those having memorized a procedure arrived at this intuitive answer. These results flesh out the interpretation of response time differences observed throughout the studies in this manuscript.

Note that the Supplementary Material presents further qualitative results and discusses responses to a number of item variants not featured in this manuscript.

## 5. General Discussion and Conclusion

As in earlier research, the degree of reported familiarity with the bat-and-ball problem reported by MTurk participants was high. Overall, the presented results can be taken both as reassuring news for the continued use of the CRT on the MTurk platform and as a note of concern regarding particular applications. There is clear evidence that some participants have either memorized solutions or remember strategies they applied to the task when encountering it before. It is potentially reassuring that false solutions do not seem to be memorized and repeated at a lower rate than correct solutions. In fact, participants with higher cognitive reflection seemed to notice the inappropriateness of memorized answers more readily, which fits with the idea of a “metacognitive disadvantage” (Bialek and Pennycook, 2017) for those with low CRT scores. If first answers are merely carried forward by some participants and learning over time is biased toward those with higher cognitive reflection, then the validity of measured scale values will be protected against repeated exposure. There might be limits to this general reassuring finding, though. Here, I discuss the two major concerns that motivated the studies, the dangers of mindless memorization and task confusion, before concluding by suggesting counter-measures to these problems.

### 5.1. The Danger of Mindless Memorization

Reproducing memorized answers or performing practiced calculations can constitute a “backdoor response strategy” (Morley et al., 2004), in that it is a “simple procedure that does not require a high level of ability” (p. 25). Crucially, this strategy would not even change test scores if all participants simply reproduced their original answers. The studies offer evidence for this type of repetition, but also demonstrate practice effects. At the same time, memorization observed across studies did not seem to be mindless for the most part. Learning did not seem to occur for randomly chosen participants but for those with higher degrees of cognitive reflection.

While this selectivity helps to maintain the validity of the scale, continued exposure to the items seems to afford learning to an increasing proportion of the population. This can result in ceiling effects (Study 2) and ultimately reduce the validity of the scale. As another consequence, for experienced participants standard norms for populations might no longer be applicable, and it might be advisable to include prior experience in models (see also Thomson and Oppenheimer, 2016). By overcoming the need for reflection, experienced participants break the link between process variables, such as response time, and outcome variables. After recognizing a previously solved item, participants will solve it faster and are unlikely to require disinhibition of the previously intuitive response. At best, a correct solution from memory can be interpreted as a signature of earlier reflection, at worst, as the outcome of vicarious learning. Further, platform-based learning beyond CRT items might constitute a confound for validation studies, if participants that learned solutions to the CRT also acquired solutions to numeracy scales or other validation measures. This may inflate true correlations and would likely be more pronounced in samples of both experienced and inexperienced participants. These are by no means safe predictions, as the platform dynamics of MTurk ultimately determine the rate of turnover, panel tenure and recruitment. But it would likewise be ill-advised to simply assume the continued validity of scales across years of repeated use without regular checks.

### 5.2. The Danger of Task Confusion

A second vulnerability of environments with experienced participants is illustrated by the high percentage of mismatched answers to the complementary variant. Tasks and item formats encountered frequently tend to be identified more readily by MTurk participants, and merely similar items might be mistaken for the familiar ones. This is a more general problem on the platform that is exacerbated by the lack of information about and the limited degree of control over a participant's previous sessions and experiments. Schneider (2015) warned that “[i]n the rush to get to the next HIT, Turkers may provide a prefab answer without internalizing the subtleties that the researcher meant to convey.” This is not a trivial problem as many experiments choose manipulations that are subtle variations of more frequently used manipulations; such as adding words to change the context or partially varying payoff structures. Thus, participants might encounter more than one experimental condition of the same experimental design across studies, which has been reported to decrease effect sizes (Chandler et al., 2015). At least the CRTt correlated to a higher degree with validation measures than the CRT in Study 2.

A related problem is posed by encountered differences in research ethics and common practices between academic requesters (e.g., Hertwig and Ortmann, 2001). For example, deception in experiments—regarded as a last resort in most ethical guidelines (Hertwig and Ortmann, 2008)—is frequently used on MTurk even where deception-free alternatives are readily available. As a consequence, trust in requesters and researchers has been eroded (Milland, 2015), causing serious problems for both users and non-users of deception. The veracity of instructions is fundamentally doubted by at least some participants (Ortmann and Hertwig, 2002). Stewart et al. (2017) likened this situation to a tragedy of the commons, where studies of one lab can “contaminate the pool for other laboratories running other studies” (p. 744). Long-term participants will perpetuate the problem of task confusion and interference between practices.

### 5.3. A Special Status for the CRT?

There is ample evidence found cross several disciplines that repeated exposure to the same test material changes response distributions. Neuroscientists (e.g., Theisen et al., 1998; Basso et al., 2002; Collie et al., 2003; Bird et al., 2004) are concerned about practice effects (Bartels et al., 2010) as they might inflate diagnostic test results. Their absence might even be diagnostically relevant (Mitrushina and Satz, 1991; McCaffrey and Westervelt, 1995; Hickman et al., 2000; Calamia et al., 2012). Repeat participants in personnel selection procedures often improve on test scores, as observed with French pilots (Matton et al., 2011) or applicants in law enforcement (Hausknecht et al., 2002) and medicine (Wolkowitz, 2011; O'Neill et al., 2015). Test practice and coaching can improve results on standardized aptitude tests (Kulik et al., 1984a,b; Arendasy et al., 2016), even without actual ability improvements (Matton et al., 2011). Likewise, the repeated use of test items in medical classroom exams over years was accompanied by a decrease in difficulty and discriminability (Joncas et al., 2018; Panczyk et al., 2018).

Looking at some of the established moderators of practice effects (e.g., Steger et al., 2018), there are some reasons why the CRT should be especially vulnerable: MTurk is an unproctored setting (Tippins et al., 2006; Carstairs and Myors, 2009), and correct responses are easy to memorize and search for, involve complex processing and a moment of realization. These properties have been linked to higher degrees of practice effects (Bornstein et al., 1987; Rapport et al., 1997; Collie et al., 2003; Reeve and Lam, 2007; Arthur et al., 2009; Calamia et al., 2012; Lezak et al., 2012).

As discussed above, realizing the falsity of previous, intuitive response might be more likely for those with higher levels of cognitive reflection. Kulik et al. (1984b) found that practice effects were more pronounced for those with higher level of abilities. Rapport et al. (1997) found higher IQ gains in repeated measurement for those with higher scores at the first testing, which they likened to a Matthew effect (the “rich get richer,” but see Basso et al., 1999; Bartels et al., 2010). Stagnaro et al. (2018), along with Bialek and Pennycook (2017) interpreted their finding of stable CRT validity in this way. In this sense, repeated exposure might even reduce measurement error by giving multiple opportunities to activate the possessed potential. This can even be linked to the use of trial rounds by economists—and to a lesser degree by psychologists (Hertwig and Ortmann, 2001)— to eliminate simple forms of misunderstanding and place participants on an equal footing.

The results presented here lend some support to the theory that CRT score improvements are predominantly restricted to participants with a higher degree of cognitive reflection, insulating its validity somewhat (but not entirely) against practice effects. This result does not easily extend to other heavily used measures on MTurk for which the two identified problems might loom as larger threats.

### 5.4. Solutions: Item Monitoring, Parallel Forms and Comprehension Checks

If MTurk were a platform maintained by researchers, keeping track of participants' testing history across all research projects might be a valuable strategy for preventing unintended double exposure or for gauging previous experience. As it stands, this type of information would likely create privacy risks for participants and require individual effort from requesters. While the number of previous HITs is not available as a variable (it can only be inferred from chosen qualifications), the self-reported number of HITs proved to be a useful proxy in this study and is simple to elicit. MTurk cannot be considered a secure test environment, psychological tests employed on the platform will be exposed to the public. The repeated use of test types vulnerable to exposure requires the choice between two different strategies to maintain test validity: monitoring the existing item pool for potentially compromised tasks or generating a larger set of test variants.

#### 5.4.1. Item Monitoring

Monitoring can be difficult (McLeod and Lewis, 1999; Zhang, 2014) and has no teeth without the ability to replace compromised items, as the “fight for pool security is ultimately a losing battle” (Davey and Nering, 2002, p. 187). It can be aided by items testing for pre-exposure (self-reports or item variants, see Study 1) or the analysis of response times (Choe et al., 2018). Item monitoring requires a continued efforts to detect problems when they occur or develop to a critical point.

#### 5.4.2. Parallel Forms

On the other hand, parallel forms of cognitive tests have reduced practice effects in the past (Kulik et al., 1984b; Benedict and Zgaljardic, 1998; Beglinger et al., 2005; Calamia et al., 2012) and can enhance test security (Burke, 2009; Guo et al., 2009; Panczyk et al., 2018). Parallel test cannot address test sophistication or learning effects (Wood, 2009; Bartels et al., 2010), but they might help to separate these from item-specific factors (Rapport et al., 1997; Hausknecht et al., 2002). Of course, these advantages can be negated if alternative forms are of different difficulty or lower validity (Davey and Nering, 2002; Calamia et al., 2012; Lezak et al., 2012). While the benefits might be increased with item generation procedures that allow at the limit for unique tests for each tested individual (Irvine, 2002), simple alternate forms (see e.g., Thomson and Oppenheimer, 2016) should be considered and evaluated on a continuous basis, when faced with returning participants.

It still seems puzzling to me why the same items are used in most studies investigating cognitive reflection. Item norms are impacted by platform dynamics over time, and Thomson and Oppenheimer (2016) called the CRT an “expendable resource” (p. 109). Simple parallel forms are still vulnerable to memorization. For example, Milland (2016) offers solutions to the CRT 2 (Thomson and Oppenheimer, 2016). The transformed items (CRTt) are a step toward a solution. Validating item sets with a common item structure and a rich collection of viable number sets would go a long way to avoid the vulnerability that a simple memorization of numbers can be mistaken for cognitive reflection, and it would avoid both construct proliferation and reliance on *ad-hoc* measures.

#### 5.4.3. Comprehension Checks

To detect instances of task confusion, researchers can employ comprehension checks that emphasize differences in the target task compared to tasks that are assumed to be encountered frequently on the platform. For example, if a task with a single participant bears resemblance to tasks encountered with player interaction, it is a good idea to stress this difference in the instructions and test for comprehension before (or after) the task. As the examples in this study demonstrate, it cannot be assumed that seemingly familiar instructions and questions are read word-by-word by experienced participants without incentives.

#### 5.4.4. Tradeoffs

The results in Study 3 put focus on some ethical tradeoffs involved in research on MTurk The principle of beneficence would advise researchers to give feedback to participants after the test to help them realize potential carelessness in their thinking. At the same time, ethical guidelines advise psychologists not to allow test stimuli to “become part of the public domain” (Tranel, 1994, p. 34) to avoid the invalidation of cognitive measures. The APA guidelines require that “[p]sychologists make reasonable efforts to maintain the integrity and security of test materials and other assessment techniques” (American Psychological Association, 2002, p. 1072). In some cases, public disclosure of test materials was found to maintain validity of the tests (Goldberg et al., 2006; Condon and Revelle, 2014). Direct harm to participants is a less likely scenario, it might occur if practice effects hide cognitive decline in medical exams and prevent proper treatment. The limited results on feedback in this study would advise caution in giving feedback on CRT answers to participants who might be tested again.

### 5.5. Conclusion

To conclude, using several variants of items featured in the cognitive reflection test, it was demonstrated that many responses on MTurk, but not on a similar platform, are influenced by test experience and exhibit practice effects. Repeated exposure has been discussed as benign in the literature, and the continuing validity of the CRT was confirmed in the present studies. The results still point at two vulnerabilities of frequently used tasks: (1) the possibility of memorization (based on personal insight or public information) that may fundamentally change the response process, and (2) the possibility of interference and noise created by mistaking a presented task for a merely similar task that was previously experienced on the platform. Both problems may impact a research project in foreseeable and—given the vast number of studies run on the platform—also unforeseeable ways. These vulnerabilities are exacerbated by the reliance on a single set of three items. Looking back a century into the beginning of intelligence testing, Davey and Nering (2002) stated that “[i]t was not unusual in the early days of psychological measurement for test developers to produce only a single form and to administer that form whenever it was needed.” (p. 167). While the Stanford Binet-Simon Intelligence scale received alternative forms in 1937, the CRT is still predominantly employed in a single form. As seen in previous research and partially replicated in this manuscript, this does not necessarily invalidate obtained results or negate the CRT's usefulness as a cognitive measure for the time being, but the observed trends give nonetheless reasons for concern. Alternative task formats that may help to address future problems with the CRT may include generative item structures that can be filled with multiple sets of numbers to prevent memorization, and Study 2 and Study 3 resulted in some promising steps in this direction. Upon reflection on cognitive reflection, validating these item structures might be a sensible step to ensure the continued productivity of cognitive reflection research, and a good approach for measures less protected against repeated exposure.

## Data Availability Statement

The datasets for Study 2 and Study 3 are available at Harvard Dataverse (Woike, 2019). The dataset for Study 1 will be available from the author for academic researchers upon reasonable request.

## Ethics Statement

The studies involving human participants were reviewed and approved by Ethics Committee of the Max Planck Institute of Human Development, Berlin. Written informed consent for participation was not required for this study in accordance with the national legislation and the institutional requirements.

## Author Contributions

JW designed and executed the research, analyzed and interpreted the data, and wrote the article.

### Conflict of Interest

The author declares that the research was conducted in the absence of any commercial or financial relationships that could be construed as a potential conflict of interest.

## Footnotes

^1^The SAT has been and still is the most widely used standardized test to determine admissions to college in the US; it was developed by the Educational Testing Service.

^2^Stieger and Reips (2016) observed several thousand search results for the exact phrase “a bat and a ball cost” (my search in June 2019 returned 12,700, a search for “a ball and a bat cost” returned 1,370 pages).

^3^It is evident that some MTurk participants acquire solutions via channels like these, as exemplified by discussions between forum users on sites like MTurkforum or TurkerHub, who admit to repeating solutions they have not understood or teach others about correct answers to the problem.

^4^Incidentally, this combination allowed workers to participate who had 51–99 HITs and did not meet the 95% standard.

^5^Assignment to conditions was random for all subsets. The Supplementary Material section 2.1.5 contains a discussion of the merits of attention checks for CRT studies, and analyzes the relationship between attention check and CRT performance.

^6^If one assumes that these participants would have given the intuitive and correct response without prior exposure, the proportion of both groups together is close to the 98% correct responses observed for a similar trivial variant in De Neys et al. (2013) or the 99% in Raoelison and De Neys (2019).

^7^A re-analysis of their published data revealed that the number of participants who solved the original but not the transformed problem was the same as the number of participants who solved the transformed but not the original problem (16% of the sample each, 37% solve both variants).

^8^Responses of zero for two participants were set to one (counting the present HIT).

^9^A re-analysis of the data in Chandler et al. (2014) revealed that participants who had solved the original problem had significantly more previous HITs than those who did not [*F*_(1,98)_ = 5.65, *p* = 0.02], which was not true for the transformed problem: [*F*_(1,98)_ = 0.08, *p* = 0.78]. Similarly, solving the original version was a significant predictor [$\chi_{\left(1\right)}^{2}=4.03$, *N* = 100, *p* = 0.045, asymptotic 2-sided test] of “Super Turker” status, while performance on the new test was not [$\chi_{\left(1\right)}^{2}=1.24$, *N* = 97, *p* = 0.27].

^10^This effect size measure is based on Dunlap et al. (1996, p. 171), as implemented in Lenhard and Lenhard (2016).

^11^Based on a reviewer's suggestion I also calculated an ANOVA without the tenure variable. In this analysis, both main effects for gender and version are stronger, but the interaction between version and gender is not significant [*F*_(1,696)_ = 2.25, *p* = 0.13, partial η^2^ = 0.003].

^12^A *p*-value of 0.051 might incline some readers to see this statement refuted. I would argue, in the spirit of divine preferences assumed by Rosnow and Rosenthal (1989) that this still constitutes evidence for the difference that does not weaken but strengthen the evidence regarding subjective numeracy.

^13^Participants who failed the initial test did not enter the study. All other exclusions were decided upon before data analysis based on data for the initial check.
