# Supplementary material for: Upon Repeated Reflection: Consequences of Frequent Exposure to the Cognitive Reflection Test for Mechanical Turk Participants
Source: Front Psychol. 2019 Dec 6;10:2646. doi: 10.3389/fpsyg.2019.02646 (PMC6909056; doi:10.3389/fpsyg.2019.02646)
Supplement: Supplementary file 1 [file Data_Sheet_1.pdf]

Supplementary Material for “Upon Repeated Reflection: Consequences of Frequent Exposure to the Cognitive Reflection Test for Mechanical Turk Participants”

1 ITEMS AND SURVEY QUESTIONS

1.1 Study 1: MTurk Data

1.1.1 Item variants

Original variant.

> A bat and a ball cost $1.10 in total. The bat costs $1.00 more than the ball. How much does the ball cost [in cents]?

Trivial variant.

> A bat and a ball cost $1.10 in total. The bat costs more than the ball. It costs $1.00. How much does the ball cost [in cents]?

Complementary variant variant.

> A bat and a ball cost $1.10 in total. The bat costs $1.00 more than the ball. How much does the bat cost [in cents]?

Transformed variant.

> A golden bat and a golden ball cost $5,000 in total. The golden bat costs $4,000 more than the golden ball. How much does the golden ball cost [in dollars]?

1.1.2 Previous exposure question

Have you seen the question about the bat and the ball before on MTurk?

- I have seen the same question before.
- I have seen a similar question before.
- I have not seen this or a similar question before.

1.1.3 Attention checks

Participants had to pass one of two attention checks presented at the beginning of the study, shown in Figure S1 and Figure S2. The second check was only shown in case the first attention check was failed.

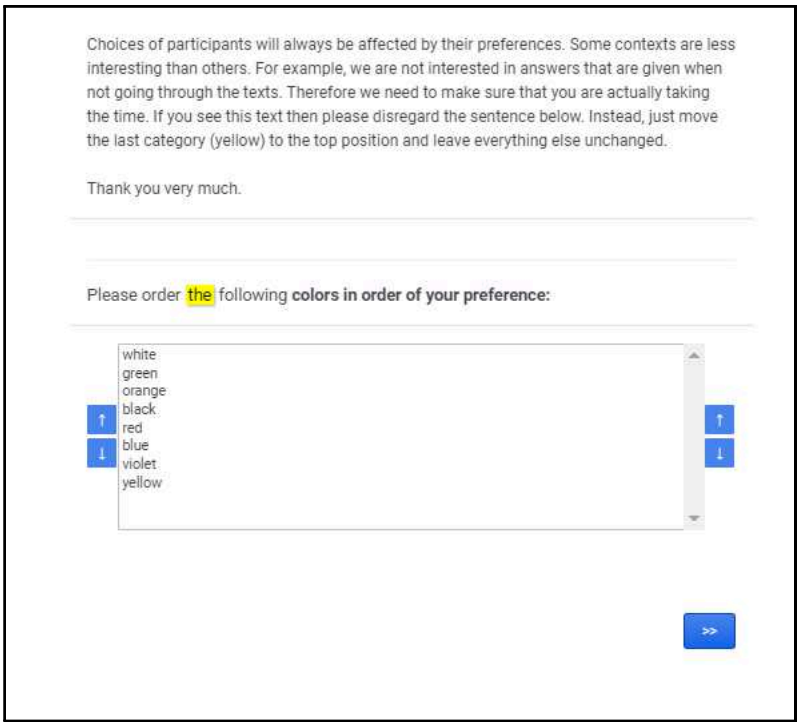

Figure S1. Attention check item 1 in Study 1

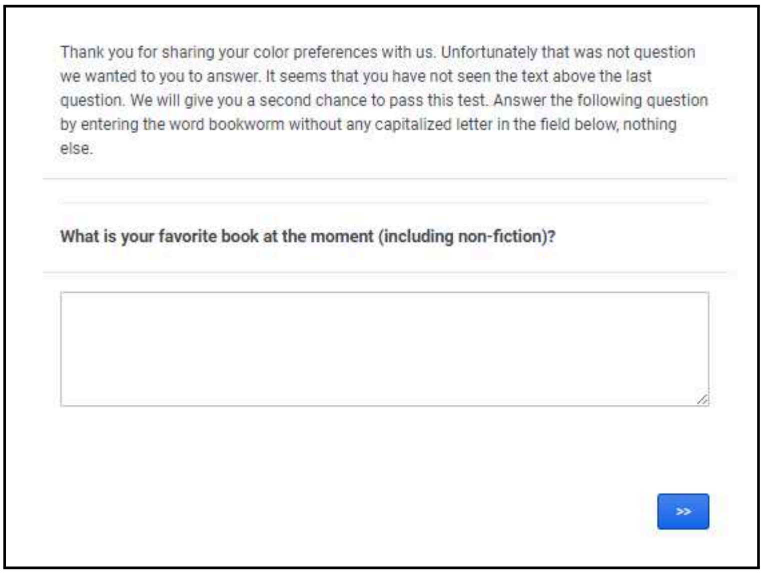

Figure S2. Attention check item 2 in Study 1

1.2 Study 1: Qualtrics Data

[*Questions were presented on separate pages.*]

1.2.1 CRT questions

Item 1. A bat and a ball cost $1.10 in total. The bat costs a dollar more than the ball. How much does the ball cost? [cents]

Item 2. If it takes 5 machines 5 min to make 5 widgets, how long would it take 100 machines to make 100 widgets? [minutes]

Item 3. In a lake, there is a patch of lily pads. Every day, the patch doubles in size. If it takes 48 days for the patch to cover the entire lake, how long would it take for the patch to cover half of the lake? [days]

1.3 Study 2

1.3.1 CRT questions

CRT 1. A bat and a ball cost $1.10 in total. The bat costs $1.00 more than the ball. How much does the ball cost [in cents]?

CRT 2. If it takes 5 machines 5 minutes to make 5 widgets, how long would it take 100 machines to make 100 widgets [in minutes]?

CRT 3. In a lake, there is a patch of lily pads. Every day, the patch doubles in size. If it takes 48 days for the patch to cover the entire lake, how long would it take for the patch to cover half of the lake [in days]?

Scale statistics. The average score was $M = 1.93$ ($SD = 1.19$). The internal consistency was measured as Cronbach's $\alpha = .78$ ($N = 700$).

1.3.2 Previous exposure question

Have you seen the first question about the bat and the ball before on MTurk?

- I have seen the same question before.
- I have seen a similar question before.
- I have not seen this or a similar question before.

1.3.3 CRTt questions

CRTt 1. A golden bat and a golden ball cost $5,000 in total. The bat costs $4,000 more than the ball. How much does the golden ball cost [in $]?

CRTt 3. In a lake, there is a patch of lily pads. Every day, the patch doubles in size. If it takes 40 days for the patch to cover the entire lake, how long would it take for the patch to cover a quarter of the lake [in days]?[1]

CRTt 2. If it takes 10 machines 10 minutes to make 10 widgets, how long would it take 1,000 machines to make 1,000 widgets [in minutes]?

Scale statistics. The average score was $M = 1.60$ ($SD = 1.13$). The internal consistency was measured as Cronbach's $\alpha = .66$ ($N = 700$).

1.3.4 Financial Literacy Test

From Hastings et al. (2013):

FL 1. Suppose you had $100 in a savings account and the interest rate was 2% per year. After 5 years, how much do you think you would have in the account if you left the money to grow?

- More than $102

[1] Item 3 was presented before item 2 for the CRTt.

- Exactly $102
- Less than $102
- Don't know

FL 2. Imagine that the interest rate on your savings account was 1% per year and inflation was 2% per year. After 1 year, would you be able to buy more than today, exactly the same as today, or less than today with the money in this account?

- More than today
- Exactly the same as today
- Less than today
- Don't know

FL 3. Do you think that the following statement is true or false: Buying a single company stock usually provides a safer return than a stock mutual fund?

- True
- False
- Don't know

FL 4. Do you think that the following statement is true or false: Buying a single company stock usually provides a safer return than a stock mutual fund?

- True
- False
- Don't know

FL 5. If interest rates rise, what will typically happen to bond prices?

- They will rise.
- They will fall.
- They will stay the same.
- There is no relationship.
- Don't know

Scale statistics. Table S1 shows solution rates in the sample ($N = 700$) to each of the five items of the scale. The average score was $M = 3.7$ ($SD = 1.18$). The internal consistency was measured as Cronbach's $\alpha = .56$).

Table S1. Proportions of correct solutions to the five financial literacy items

Item	Correct [%]
FL1	89.9
FL2	78.9
FL3	77.7
FL4	87.9
FL5	35.7

1.3.5 Subjective Numeracy

From Fagerlin et al. (2007):

For each of the following questions, please check the box that best reflects **how good you are at doing the following things**:

SN 1. How good are you at working with fractions? [*Scale: 1–Not at all good to 6–Extremely good*]

SN 2. How good are you at figuring out how much a shirt will cost if it is 25% off? [*Scale: 1–Not at all good to 6–Extremely good*]

SN 3. For the following question, please check the box that best reflects your answer:

How often do you find numerical information to be useful? [*Scale: 1–Never to 6–Very often*]

Scale statistics. Table S2 shows solution rates in the sample ($N = 700$) to each of the three items of the scale. The average score was $M = 13.7$ ($SD = 3.09$). The internal consistency was measured as Cronbach's $\alpha = .80$).

Table S2. Proportions of correct solutions to the three subjective numeracy items

Item	Mean	SD
SN1	3.81	1.42
SN2	4.86	1.20
SN3	5.00	1.01

1.4 Study 3

1.4.1 Complete list of items

Most participants answered five questions that were either original CRT-items or variants. Participants answered one question out of each of five blocks: (1) one of five variants of the original first item (I1), (2) one of six variants of the original second item (I2), (3) I2, (4) I1, (5) one of three novel items.

Study 3 also extended on the previous studies by including a larger range of transformed item variants with systematically varied numbers and story elements to test the degree of interference caused by superficial cues (Lee et al., 2015; Morley et al., 2004; Ross, 1989). All items are listed in Table S3. Three of the variants for each original items were equivalent variants. One pair of variants (AO ,SO) used the same numbers as the respective original item, but a different story, transforming the bat-and ball example into a story about the age of grandfather and grandchild (AO), and the widget example into a social media story (SO). A second pair (BT, WT) used the same story as the original item, but transformed the numbers. Note that the WT example did not follow the template in the SM, as the number of machines, minutes and widgets were not the same[2]. A third pair (AT, ST) changed both the story and transformed numbers (using a different transformation than the second pair). All changes made for these groups of items were superficial or surface changes (Holyoak and Koh, 1987) that preserved the solutions strategy while changing incidental elements of items (Irvine, 2002). In the case of unchanged numbers, this left the solutions themselves unaffected. Two of the three novel variants were designed to be lure items, the third offered at least misleading intuitions[3]. The remaining variants were non-equivalent and non-isomorphic variants

[2] This deviation was introduced after observing the similarity in responses to CRT2 and CRTt2.

[3] The simplest correct solution procedure uses the fact that each game eliminates exactly one unique team, so that after 31 games 31 teams are eliminated to determine the last team as winner.

(Reed, 1987) that were constructed to differentiate participants with insight into the problems from those blindly applying solution procedures. These variants (B4/A4 for I1, WQ/SQ and WI/SI for I2) were presented in both story versions to test to which degree similarity to the original might interfere with solving the variants.

The first 288 participants answered either I2 or one of its variants as block 2, but not both, and skipped block 3, answering only four questions (at this point, an additional item was added, as the observed completion times for the HIT were faster than expected).

Table S3. Items presented in Study 3: abbreviation, block, item text, solution(s) coded as correct and intuitive

ID	Bl.	Question	Corr.	Int.
B4	1	A bat and four balls cost $1.10 in total. The bat costs 1 dollar more than a single ball. How much does a ball cost? [in cents]	2	2.5
BT	1	A golden bat and a golden ball cost $5,000 in total. The golden bat costs $4,000 more than the golden ball. How much does the golden ball cost? [in $]	500	1,000
A4	1	A grandfather tells his four grand-children: ”‘Together we are 110 years old, but I am 100 years older than each of you.”’ How old is a grand-child? [in years]	2	2.5
AT	1	A grandfather tells his grand-child: ”‘Together we are 82 years old, but I am seventy years older than you are.”’ How old is the grand-child? [in years]	6	12
AO	1	A grandfather tells his grand-child: ”‘Together we are 110 years old, but I am 100 years older than you.” How old is the grand-child? [in years]	5	10
WT	2	If it takes 20 machines 80 minutes to make 200 widgets, how long would it take 40 machines to make 400 widgets? [in minutes]	80	160
WQ	2	If it takes 20 machines 20 minutes to make 20 widgets, how long would it take 10 machines to make 40 widgets? [in minutes]	80	40
WI	2	If it takes 5 machines 5 minutes to make 5 widgets, how many widgets would be produced by 10 machines in 10 minutes?	20	10
SO	2	If it takes 5 social media bots 5 seconds to send 5 messages, how long would it take 100 social media bots to send 100 messages? [in seconds]	5	100
ST	2	If it takes 10 social media bots 10 seconds to send 20,000 messages, how long would it take 20 social media bots to send 40,000 messages? [in seconds]	10	20
SQ	2	If it takes 10 social media bots 10 seconds to send 20,000 messages, how long would it take 5 social media bots to send 40,000 messages? [in seconds]	40	20
SI	2	If it takes 5 social media bots 5 seconds to send 5 messages, how many messages would be sent by 10 social media bots in 10 seconds?	20	10
I2	3	If it takes 5 machines 5 minutes to make 5 widgets, how long would it take 100 machines to make 100 widgets? [in minutes]	5	100
I1	4	A bat and a ball cost $1.10 in total. The bat costs $1.00 more than the ball. How much does the ball cost? [in cents]	5	10
N1	5	Peter has four friends. Together they are able to carry 40 boxes. If Peter had 20 friends instead, how many boxes would they be able to carry?	160/168	200
N2	5	If you divided a long baguette by four cuts into even pieces, each piece would be 18 cm long. How long would a piece be if you did it with eight cuts? [in cm]	10	9
N3	5	In a sports tournament, matches in each round were played by two teams against each other and only the winner was able to proceed to the next round. It took a total of 31 matches to determine the final winner.How many teams participated in this tournament?	32	16/62

1.4.2 Block 1: Bat-and-ball variants

CRT-A Ballfour

B4.

A bat and four balls cost $1.10 in total. The bat costs 1 dollar more than a single ball. How much does a ball cost? [in cents]

BT.

A golden bat and a golden ball cost $5,000 in total. The golden bat costs $4,000 more than the golden ball.
How much does the golden ball cost? [in $]

1.4.2.1 A4

A grandfather tells his four grand-children: ”Together we are 110 years old, but I am 100 years older than each of you.”
How old is a grand-child? [in years]

AO.

A grandfather tells his grand-child: ”Together we are 110 years old, but I am 100 years older than you.”
How old is a[4] grand-child? [in years]

AT.

A grandfather tells his grand-child: ”Together we are 82 years old, but I am seventy years older than you are.”
How old is the grand-child? [in years]

1.4.3 Block 2: Widget variants

WT.

If it takes 20 machines 80 minutes to make 200 widgets, how long would it take 40 machines to make 400 widgets? [in minutes]

WQ.

If it takes 20 machines 20 minutes to make 20 widgets, how long would it take 10 machines to make 40 widgets? [in minutes]

WI.

[4] This was a typo in the study. As this item was never presented together with one of the variants with four elements, there was no ambiguity. The item has been corrected in the manuscript (“a” replaced by “the”).

If it takes 5 machines 5 minutes to make 5 widgets, how many widgets would be produced by 10 machines in 10 minutes?

SO.

If it takes 5 social media bots 5 seconds to send 5 messages, how long would it take 100 social media bots to send 100 messages? [in seconds]

ST.

If it takes 10 social media bots 10 seconds to send 20,000 messages, how long would it take 20 social media bots to send 40,000 messages? [in seconds]

SI.

If it takes 10 social media bots 10 seconds to send 20,000 messages, how long would it take 5 social media bots to send 40,000 messages? [in seconds]

SQ.

If it takes 5 social media bots 5 seconds to send 5 messages, how many messages would be sent by 10 social media bots in 10 seconds?

1.4.4 Block 3–4: Original CRT items

I2 (Block 3).

If it takes 5 machines 5 minutes to make 5 widgets, how long would it take 100 machines to make 100 widgets? [in minutes]

I1 (Block 4).

A bat and a ball cost $1.10 in total. The bat costs $1.00 more than the ball.
How much does the ball cost? [in cents]

1.4.5 Postquestionnaire

Have you encountered the previous question about a bat and a ball before (anywhere)? (Note that your specific responses to this and the following questions will not affect your payment nor restrict your participation in future studies.)

- yes
- no

[The survey branches into two parts after this question, dependent on the answer.]

Questions if encountered.

Where have you encountered the bat-and-ball question before? (Please click all that apply.)

- I have not encountered this question before.

- In a book/newspaper/journal (please name the source, if you remember the title(s))
- On an internet forum (please name the forum, if you remember it)
- In a lecture/class/presentation
- While doing a HIT on MTurk
- Somewhere else (please describe where, if you remember it)

[New page]

How many times have you had to answer the bat-and-ball question so far (please guess, if necessary)? Please write ”1” if this is the first time.

How did you arrive at the answer to the bat-and-ball question?

- I knew the answer from memory
- I knew how to calculate the answer from memory
- I did not know the answer before the task

[New page]

Have you ever been offered bonus money on MTurk for the correct answer to the bat-and-ball question?

- yes
- no

Have you ever received feedback on MTurk on whether your answer to the bat-and-ball question was correct or not?

- yes, I was given the correct answer
- yes, but only ”correct”/”false”
- no

What is your opinion about the bat-and-ball question? Are there other questions or tasks that you feel similar about?

Questions if not encountered.

Have you searched for the answer to the bat-and-ball question or received the correct answer from someone else?

- I found the answer on my own.
- I searched for the answer online.
- I searched for the answer elsewhere.
- Someone told me the answer/I read it somewhere by accident.

If you found the answer somewhere, where did you find it? (leave this field blank, if you did not find it anywhere)

What is your opinion about the bat-and-ball question? Are there other questions or tasks that you feel similar about?

1.4.6 EV-scale

[Asterisks mark answers that were scored with 1, unmarked answers were scored as 0.]

Item 1.

What would you prefer?

- $3400 this month, or
- $3800 next month (*)

Item 2.

What would you prefer?

- $500 for sure, or
- a 15% chance of $1,000,000 (*)

1.4.6.1 Item 3

Item 3.

What would you prefer?

- $100 for sure, or
- a 75% chance of $200 (*)

Scale statistics. The average score on the three-item scale was $M = 1.71$ ($SD = .965$, $N = 1003$). Cronbach's α was estimated as $\alpha = .37$. Note that this internal consistency measure is likely to underestimate the reliability of the scale, as the items were not chosen to be parallel. The three items were selected from eighteen items administered by Frederick (2005, see Table 3a); all three items showed substantial differences between high and low scorers in the original CRT.

1.4.7 Block 5: Exploratory novel items

[Every participants answered these questions.]

N1.

Peter has four friends. Together they are able to carry 40 boxes.
If Peter had 20 friends instead, how many boxes would they be able to carry?

N2.

If you divided a long baguette by four cuts into even pieces, each piece would be 18 cm long.
How long would a piece be if you did it with eight cuts?

N3. *Note: As this item is not truly a lure item (as seen below, the majority of responses is neither corect nor "intuitive"), it was not reported in the main manuscript.*

In a sports tournament, matches in each round were played by two teams against each other and only the winner was able to proceed to the next round. It took a total of 31 matches to determine the final winner.

How many teams participated in this tournament?

1.4.8 Introductory attention and comprehension checks

Participants had to pass two of the three consecutive items shown below in Figure S3, Figure S4, and Figure S5. Note that only the third item is an IMC item, the other two items discriminate between US participants and those spoofing their location.

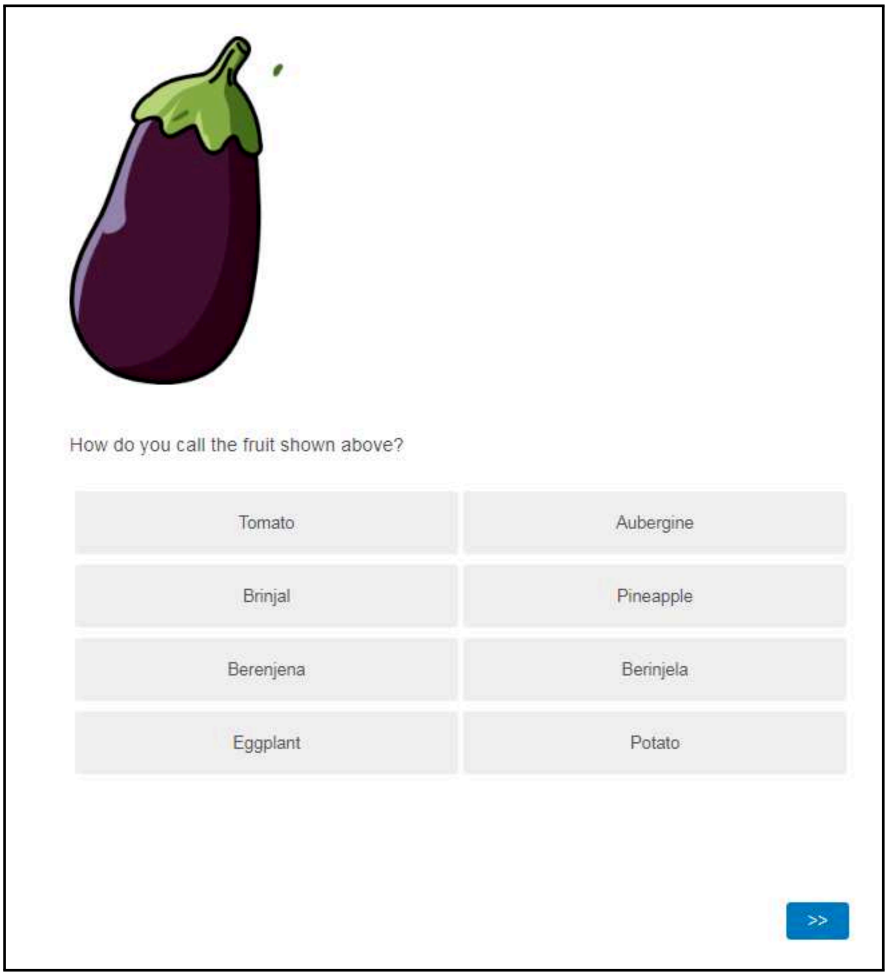

Figure S3. Attention check item 1 in Study 3

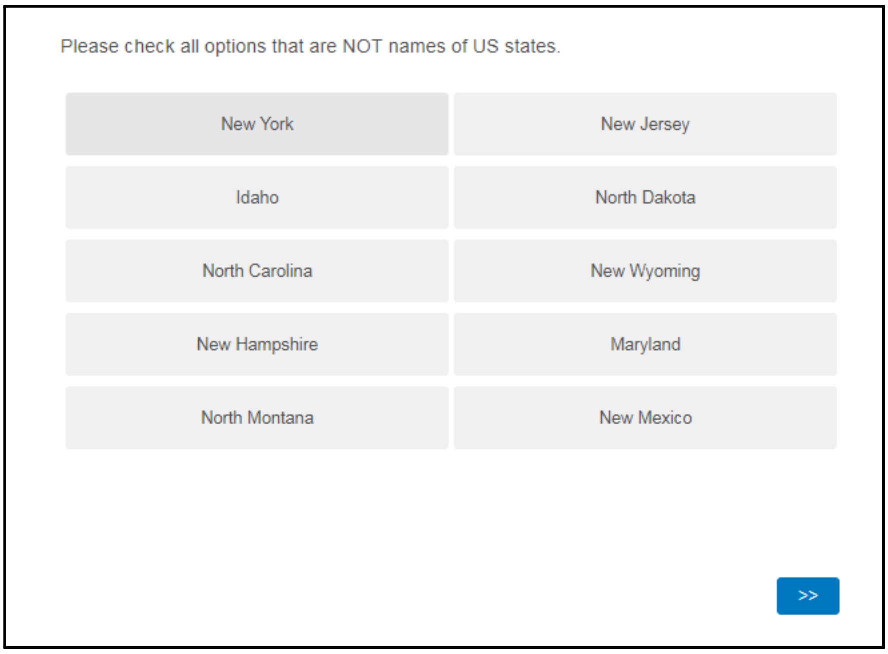

Figure S4. Attention check item 2 in Study 3

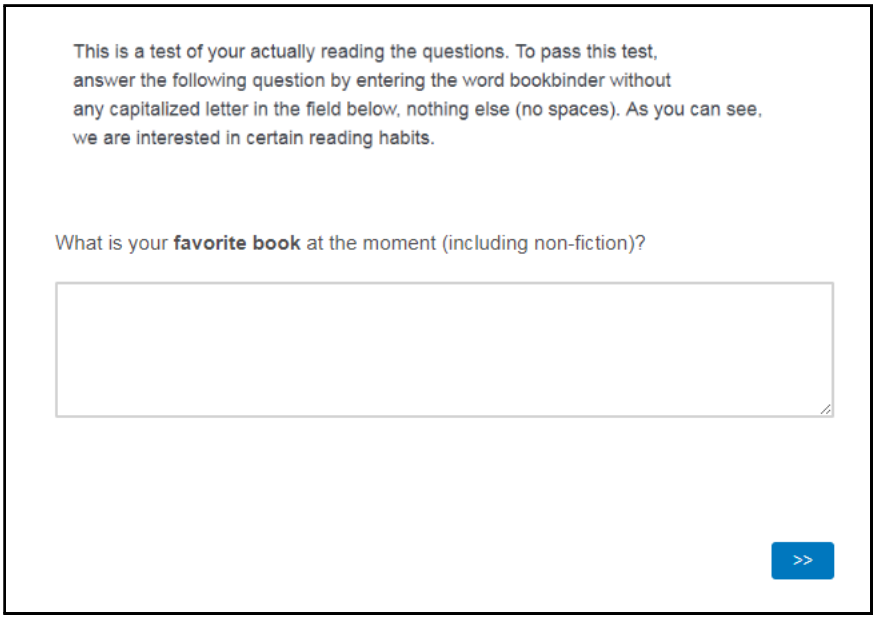

Figure S5. Attention check item 3 in Study 3

2 SUPPLEMENTARY ANALYSES

2.1 Study 1

2.1.1 Log-Transformation of previous HITs and RTs

Figure S6 shows an example of the distribution of the number of HITs before and after $log_1 0$-trasnformation. The transformed, but not th euntransformed distribution is approximately symmetric.

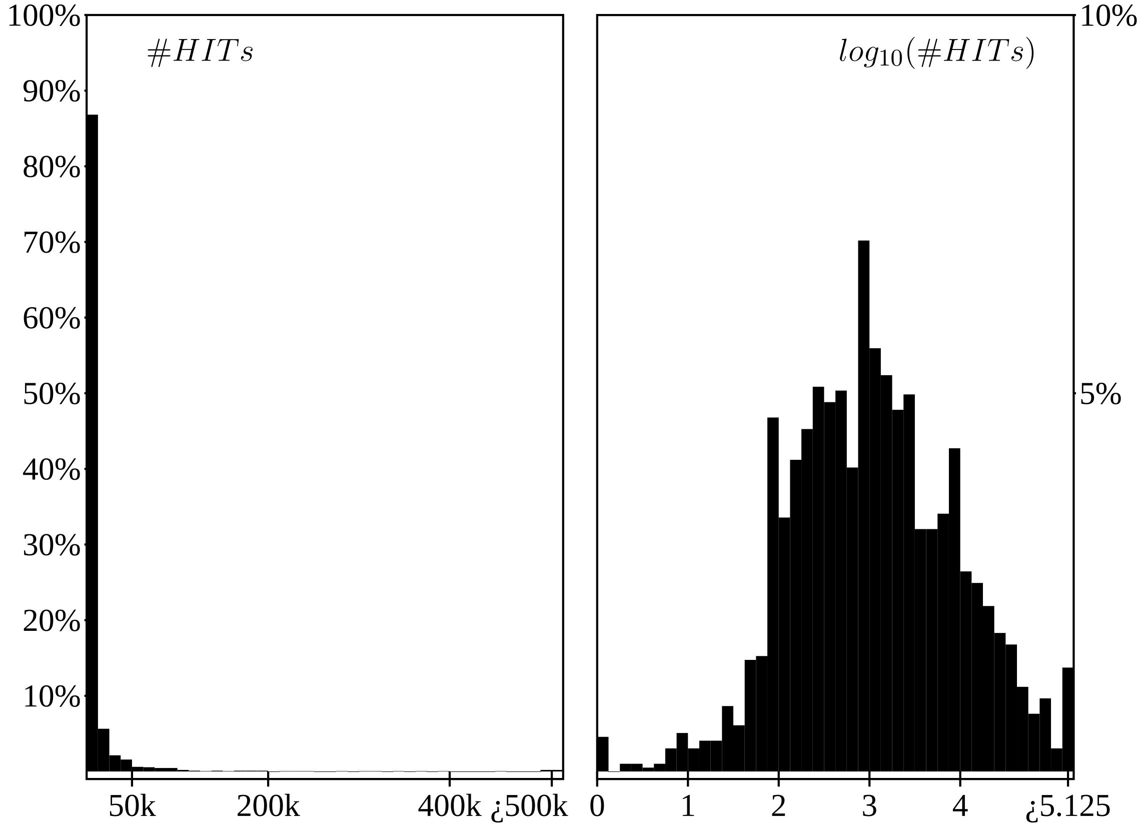

Figure S6. Distribution of reported number of previous HITs across participants: the left panel shows the untransformed numbers, the right panel the transformed numbers . Large values were bundled into a single category for the graphical presentation only.

2.1.2 Extended results: Prior exposure to the CRT

Participants were also asked whether they had seen the same problem before, a similar problem before, or neither the same nor a similar problem before. This question was specific to the problem encountered in each of the four groups. The latter category is plotted dependent on answer categories in Figure S7, right column . Some results cast the veracity of some participants' answers into doubt; pretending unfamiliarity might be seen as a socially desirable response or a response that maximizes the chances of being eligible for study participation (Chandler and Paolacci, 2017). The general pattern is still informative and yields comparable results to the previous analyses.

Most people (69.3%) admitted to have seen the original problem before. More participants with false, intuitive answers described the task as novel than participants with correct answers ($d = 17.1\%$, $95\%\ CI = [8.5\%, 25.2\%]$). Note that only roughly one in five correct participants had encountered the problem for the first time.

Again, most people (64.1%) claimed to have seen the complementary variant before. In contrast to the original variant, unfamiliarity with the problem seemed to convey an advantage for the complementary problem, at least for choosing the right focus object for the response. The variant was seen as novel by 28.7% of those who answered (incorrectly) with a price for the ball, and by 43.2% of those who answered with a price for the bat ($d = 14.6\%$, $95\%\ CI = [5.6\%, 23.4\%]$)[5]. Among participants focusing on the bat, the correct response was given by participants who had seen the problem before at a higher rate (62.9%) than by participants who had not (41.3%, $d = -21.6\%$, $95\%\ CI = [-34.9\%, -7.1\%]$).

Virtually all participants (93.7%) who answered "5" in response to the trivial variant indicated to have seen the problem before as opposed to only 66.3% of the participants answering correctly ($d = -27.3\%$, $95\%\ CI = [-33.5\%, -17.4\%]$). Finally, the transformed problem was regarded as unfamiliar by a larger proportion of participants (46.3%) than the original problem (30.6%, $d = -15.7\%$, $95\%\ CI = [-21.5\%, -9.7\%]$). Again, correct respondents were more familiar with the task (62.3% familiarity) than respondents with the intuitive answer (48.1%, $d = -14.1\%$, $95\%\ CI = [-22.5\%, -5.4\%]$). The results in Meyer et al. (2018) similarly showed that a larger percentage of participants who had encountered the original CRT before (40%, $N = 1,610$) solved the transformed question than of naive participants (33%, $N = 3,060$ Meyer et al., 2018, as presented in Table F).

These results simultaneously validate–in spite of some concerns expressed above—the familiarity question for measuring previous exposure, which has been subject to some debate (see, e.g., Raoelison and De Neys, 2019).

2.1.3 Details about the Qualtrics Study

The sample was recruited in 2018 by Qualtrics.com, restricted to adult U.S. residents. The Qualtrics data were collected in the context of [*removed for peer review*] and drawn from a panel of reportedly more than 5 million Americans, aiming for a representative sample concerning gender, age, and income. Participants were compensated through Qualtric's incentive scheme. The analysis here is restricted to the CRT items, response times for the CRT items, and demographic variables. CRT data were collected for $n_2 = 1,238$ participants. Participants were US Americans and on average 45.9 years old ($SD = 16.6$ years), 55.9% of them categorized themselves as female (44.1% as male). A smaller group of participants ($n_1 = 221$,

[5] Here, it again seems implausible that participants valuing the ball and not the bat are indeed naive to the paradigm as one in four of them claim (answers valuing the bat are virtually non-existent in the original variant).

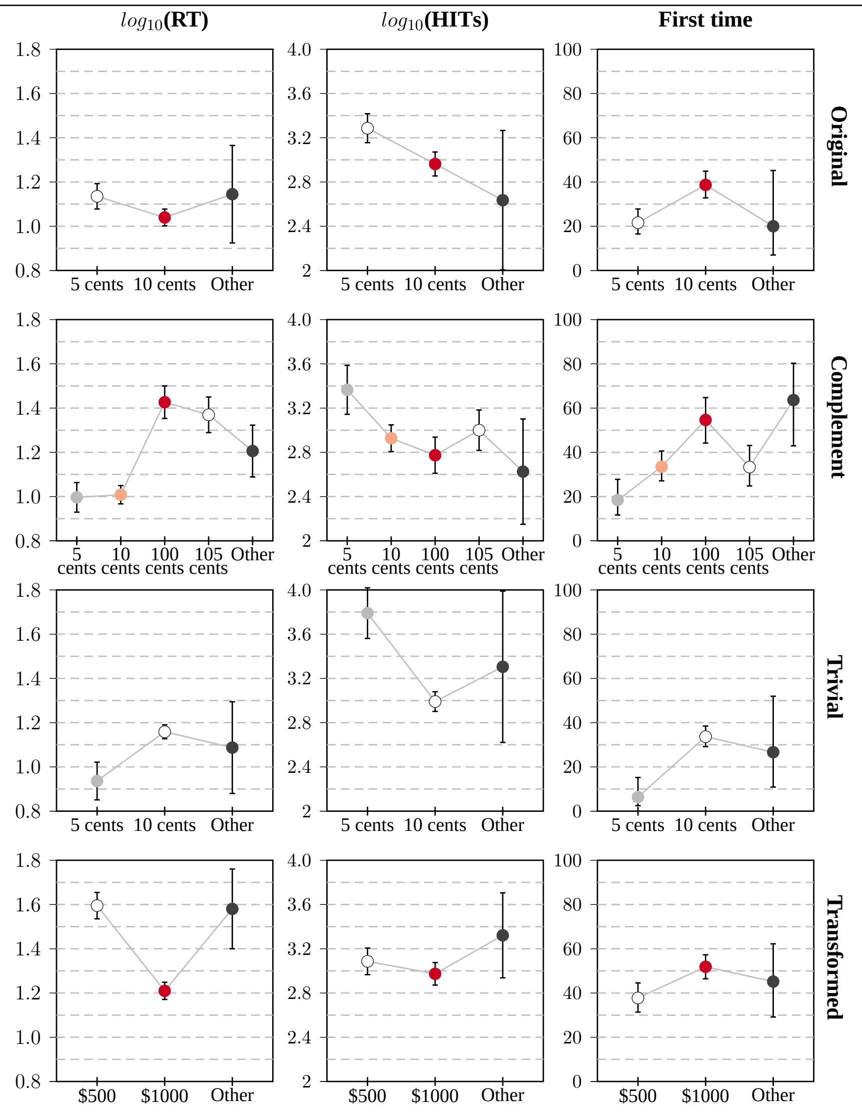

Figure S7. Relationship between response categories and response time, number of HITs, and first-time encounter in Study 1: Plots show average values of the log-transformed response time (left column), the log-transformed number of previous HITs (middle column), and the proportion of first-time solutions (right column); each row contains three plots for one of the four task variants (from top to bottom: original, complementary, trivial, transformed); whiskers correspond to the 95% CI of the mean or proportion.

17.9%) successfully completed one attention check (AC), a second group ($n_2 = 1,017$) two checks. Both groups are included in the analysis. Information about panel tenure was not available.

Qualtrics participants responded to the original variant of the bat-and-ball problem only, followed by the other two original CRT items, presented on separate pages, with response times collected for each item, separately.

There was a significant difference between the larger group of participants (82.1%) filtered with two ACs and those that only passed one ($F(1, 1236) = 6.46$, $p = 0.01$, $partial\ \eta^2 = .005$). The first group reached an average score of .47 compared to a score of .32 for the second group. Regarding the bat-and-ball problem, there was a significant difference between the larger group of participants (82.1%) filtered with two ACs and those that only passed one ($F(1, 1236) = 6.46$, $p = 0.01$, $partial\ \eta^2 = .005$). The first group reached an average score of .47 compared to a score of .32 for the second group. Regarding

the bat-and-ball problem, 9.1% of participants with a single AC and 12.3% of participants with two ACs solved the item correctly.

2.1.4 Bat-and ball answers, gender and household income

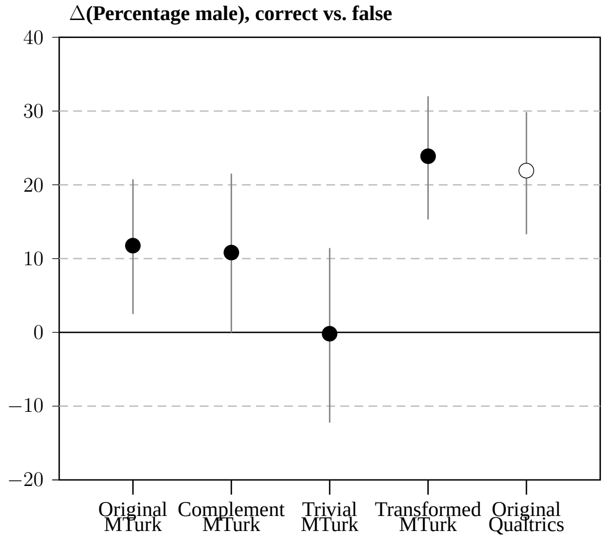

Figure S8. 95% CIs for the difference in proportion of male participants between respondents who solve each of the four task variants and those who do not; positive values indicate a higher proportion of male participants among those who solve the task. Difference CIs that do not overlap zero indicate significant differences (two-sided). Results for the original variant are shown both for the MTurk sample and the Qualtrics sample.

Neither Study 1 nor the Qualtrics data featured measures that could be considered directly relevant for estimating the potential attenuation of validity. At the same time, gender has been observed to be strongly correlated with solving the original task (with more male participants solving the task) This allows for a comparison of the four item variants in Study 1 with the original item in the Qualtrics dataset in terms of differential solution rates for male and female participants.

The relationship between success in solving each of the four tasks and gender is shown in FigureS8. A gender effect (higher success rates for male participants) was observed for the original task, but only a reduced effect for the complementary task and no effect for the trivial task. Crucially, the difference in success rates for the transformed variant ($d = -22.5\%$, 95% $CI = [-30.2\%, -14.4\%]$) was about twice the size of the difference for the original ($d = -11.6\%$, 95% $CI = [-20.6\%, -2.5\%]$). For the Qualtrics sample, the effect for the original item ($d = -21.9\%$, 95% $CI = [-29.9\%, -13.3\%]$) is similar to the effect for the transformed item on MTurk, and distinctly larger than the effect for the original item on MTurk.

It might be argued that the relationship between gender and CRT performance can hardly be the most essential property, but this would still constitute a discrepancy from earlier findings establishing a strong and robust gender component (see, e.g., Cueva et al., 2016; Frederick, 2005). Researchers consistently reported gender differences in the same direction: Scores for male participants were higher than those of female participants (Campitelli and Labollita, 2010; Campitelli and Gerrans, 2014; Cueva et al., 2016; Toplak et al., 2014). Summed over three items, Frederick (2005) reported scores of 1.47 and 1.03 for male and female participants, respectively (with two thirds of the high-scoring participants male and two thirds

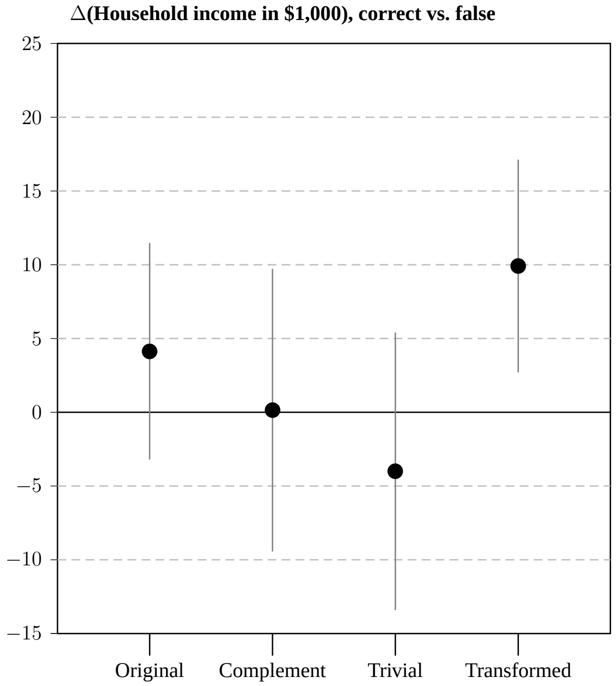

Figure S9. 95% CIs for the difference in average household income (in $1000) between respondents who solve each of the four task variants and those who do not; positive values indicate a higher average income among those who solve the task.

of the low-scoring participants female). For the bat-and-ball problem, Brañas-Garza et al. (2015) found about a 12 percent gender difference in producing the correct answer in their meta-analysis (38% for male vs. 26% for female participants, estimated from Figure 3[6]; with over 38,000 observations from 118 studies between 2005 and 2014), and larger differences for the other two questions. Based on this comparison, the observed difference for the original item does not seem to be diminished.

An analysis conducted before collecting the data for testing the CRTt found another difference between the different item version regarding the reported household income (see Figure S9). Household income served as an imperfect measure of economic success in life and was measured on a 10-point scale[7] Based on interval midpoints, participants reported a mean household income of $52.2k ($SD =$ $40.1k). A (non-significant) difference of nearly $5,000 was observed between participants solving the original task, no difference for the complementary task, and a (non-significant) difference in the opposite direction for the trivial task. Similar to the reported gender differences, the difference for the transformed task ($d = 10.13K$, $95\%\ CI = [2.96K, 17.30K]$) was three times as high as that for the original task ($d = 2.81K$, $95\%\ CI = [-4.46K, 10.09K]$), for which the confidence interval overlaps zero.[8]. Analyzing household income data for Study 2, I found that neither test score was predictive of household income.

[6] 11.3%, based on the regression coefficient in Table 1

[7] Intervals were: (1) [$0k, $5k[, (2) [$5k,$10k[, (3) [$10k,$20k[, (4) [$20k,$30k[, (5) [$30k,$40k[, (6) [$40k,$50k[, (7) [$50k,$75k[, (8) [$75k,$100k[, (9) [$100k,$150k[, (10) $>$$150k, responses were coded as interval midpoints, and as 175k for the highest bracket (3.2% of the sample).

[8] Note that I did not observe an effect of gender on household income. The main effect of solving the transformed variant was significant, when gender was added as a factor ($F(1, 533) = 7.30$, $p = 0.007$, $partial\ \eta^2 = .013$), which was not true for the main effect of solving the standard variant ($F(1, 440) = 0.73, p = .39, partial\ \eta^2 = .002$)

2.1.5 CRT performance and attention checks

Should attention checks be used for CRT studies?. As a reviewer pointed out during the review process, all studies in the manuscript employ filters based on attention check. Partcipants who fail a certain number of consecutive attention checks at the beginning are not allowed to enter the studies (they are asked to return the HIT). One can make arguments for and against the notion that this constitutes a problem for the interpretation of the observed results.

On the negative side, it can be argued that CRT items and many attention checks, such as typical instructional manipulation checks (Oppenheimer et al., 2009), share some structural features: The seemingly obvious answer is invalidated by careful analysis. Hauser and Schwarz (2015) provides evidence that IMCs go beyond measuring attention, but can act as interventions that can change subsequent response patterns. Thus, studies using IMCs might both select out a relevant sub-population and—in addition—change the behavior of the remaining participants.

There is certainly merit to this argument, but there are strong reasons for using attention checks on MTurk, especially in the form employed in these studies. One central issue in interpreting the failure of correctly responding to attention checks is the difference between ability , attention, and motivation. Inattentive participants cannot be easily distinguished from those who lack motivation or try to satisfice (Krosnick, 1991), nor can they be distinguished from those who do not have the necessary ability level to pass the test. Those participants include participants without the required level of language competence. All three groups might try to enter HITs in varying proportions, and their inclusion has a number of negative consequences for data quality, sometimes in surprising directions. Specifically, for studies using ability measures rather than scaled questionnaire items, it is unlikely that these participants will solve these items (depending on the type of item, this might be less true for one or two of the groups). It is therefore likely that participants will fail to solve multiple items. Instead of increasing the noise in measurement, this can easily result in an increase of internal consistency measures, correlations between ability scales (e.g., CRT with numeracy), etc. Further, it is not exactly clear what is learned by an analysis of these problematic group, unless a range of other measures is used to identify its composition.

While this, in principle, might constitute an interesting project for future research, there are platform dynamics to consider that make this group a moving target. A recent development illustrates the perils in this approach. While Peer et al. (2014) advocated to replace attention checks by reputation filters, a series of events during the summer of 2018 changed the attitudes of many requesters on MTurk. After an initial concern about "bots" filling in surveys, later accounts identified the source of these questionable answers as Turkers (many located in Venezuela Kennedy et al., 2018) spoofing their location and masquerading as US residents. Many of these participants gave seemingly random answers due to language problems. As seen in Study 3, which was conducted a few months after this crisis, multiple checks were added to the survey in addition to VPN-checks that turned out to be essential in preventing the participation of several hundred Turkers who were identified as problematic based on location or language skill. Even more disconcerting, the pattern of attempts to enter the HIT revealed the clustering of respondents: Multiple similar responses were given by Turkers with different MTurk IDs that were seemingly distributed across the US. Based on the analysis of data from previous studies, it cannot be excluded that up to 40 participants in clusters are non-independent, either one person with access to multiple MTurk IDs (these seem to be traded at least in some forums) or multiple persons acting as a communicating group. Accepting these "participants" into HITs and paying them for their performance might inadvertently support a business model that could threaten the viability of the platform. Thus, based on the arguments above and recent

developments, it is somewhat unclear what could be learned from an analysis of CRT answers from participants who fail attention checks.

Attention checks in Study 1. Nonetheless, the data from Study 1 allows to make some steps in this direction. Note that Study 1—conducted before the "crisis" described above—used two consecutive attention check (described above). Participants who failed the first check were informed about this fact during the second check and only participants who failed both checks were advised to return the HIT (they were also given a code containing the word "fail" with the explicit instruction not to submit the code. Only participants who submitted this code had to be rejected. Based on submission comments, this group included several participants with severe deficits in English. Further, as described in the manuscript, a part of the sample was not filtered with attention checks. This created a setup that allows to test the relationship between panel tenure,.attention check performance, and CRT results. There was one group without attention checkc, one group who failed the first attention check (and passed a second check), and a third group that passed the first attention check. All groups were randomly assigned to conditions.

Panel tenure and attention checks. Table S4 cross-tabulates panel tenure and AC group membership. Failure rate is calculated as the proportion of participants who failed the first AC among those who completed one of the two intial ACs (participants who failed both ACs did not reach the CRT question).

Table S4. Panel tenure and AC performance

	No AC		AC passed		AC failed		Failure rate
Tenure	n	perc	n	perc	n	perc	perc
-99 Hits	133	17.1	51	4.8	9	7.2	15.0
100-999 Hits	289	37.1	382	36	66	52.8	14.7
1k-9,999 Hits	239	30.6	432	40.7	38	30.4	8.1
10k-99,999 Hits	106	13.6	174	16.4	11	8.8	5.9
>=100k Hits	13	1.7	22	2.1	1	0.8	4.3
Total	780	100	1,061	100	125	100	10.5

Failure rates consistently decrease with panel tenure from 15% for the most ineperienced group to 4.3% for the most experienced group. This resulted in different compositions of the two groups with AC questions: Most of the participants (about 60%) who failed the first AC had fewer than 1,000 HITs, whereas most participants (also around 60%) in the group who passed the first test had more than 1,000 HITs.

In the group without ACs, most percentages were in between the two other groups, with the exception of the most inexperienced group. This group was relatively over-represented in the group without ACs. This could in theory be due to a higher rate of failures in both ACs for inexperienced participants, but it could also be due to variations in the participant population during different days of the data collection (the assignment to experimental conditions was randomized on each day, but the group without ACs participated at a different time than the other groups.

Attention check performance and responses to item variants. Table S5 lists the number of respondents of a given type in each of the three AC groups, for all four item variants in Study 1.

Failure rates are higher for participants that give intuitive responses than for participants with correct responses. Participants without ACs showed a proportion of correct responses between the proportions of the two other groups for the standard, complementary, and transformed item variants. For the trivial

Table S5. Item response categories and AC performance for all participants in Study 1: Each row lists the number of respondents in each group (with percentages relative to AC groups). The notation "(O)" refers to responses that were correct or intuitive for the original question.

		No AC		AC passed		AC failed		Failure rate
Item	Response	n	perc	n	perc	n	perc	perc
Standard	correct	68	38.4	120	48.4	11	34.4	8.4
	intuitive	104	58.8	118	47.6	21	65.6	15.1
	other	5	2.8	10	4	0	0	0
	total	177	100	248	100	32	100	11.4
Trivial	correct	151	84.4	219	82.3	21	65.6	8.8
	correct (O)	22	12.3	39	14.7	11	34.4	22.0
	other	6	3.4	8	3	0	0	0
	total	179	100	266	100	32	100	10.7
Complementary	correct	44	21.9	51	20.2	4	15.4	7.3
	intuitive	38	18.9	44	17.5	4	15.4	8.3
	correct (O)	36	17.9	49	19.4	2	7.7	3.9
	intuitive (O)	71	35.3	100	39.7	14	53.8	12.3
	other	12	6	8	3.2	2	7.7	20.0
	total	201	100	252	100	26	100	9.4
Transformed	correct	75	33.6	117	39.7	12	30.8	9.3
	intuitive	136	61	160	54.2	26	66.7	14.0
	other	12	5.4	18	6.1	1	2.6	5.3
	total	223	100	295	100	39	100	11.7

variant, participants without ACs had a slightly higher rate of correct responses, reflecting the higher number of inepxerienced participants in this group.

Results for experienced participants. Table S6 presents the same information as Table S5, but restricted to participants who reported at least 10,000 lifetime HITs. As thewre were only 12 participants in this category who failed the first AC, failure rates cannot be estimated with satisfactory precision. While there are clear perforemance differences between the groups with and without ACs for the standard item, the pattern of results is less clear for other item variants.

Table S6. Item response categories and AC performance for experienced participants (10,000 self-reported HITs and more) in Study 1: Each row lists the number of respondents in each group (with percentages relative to AC groups). The notation "(O)" refers to responses that were correct or intuitive for the original question.

		No AC		AC passed		AC failed		Failure rate
Item	Response	n	perc	n	perc	n	perc	perc
Standard	correct	14	48.3	32	64	2	66.7	5.9
	intuitive	15	51.7	17	34	1	33.3	5.6
	other	0	0	1	2	0	0	0
	total	29	100	50	100	3	100	5.7
Trivial	correct	23	67.6	31	58.5	3	100	8.8
	correct (O)	11	32.4	18	34	0	0	0
	other	0	0	4	7.5	0	0	0
	total	34	100	53	100	3	100	5.4
Complementary	correct	3	11.5	12	30.8	2	100	14.3
	intuitive	2	7.7	3	7.7	0	0	0
	correct (O)	12	46.2	12	30.8	0	0	0
	intuitive (O)	8	30.8	11	28.2	0	0	0
	other	1	3.8	1	2.6	0	0	0
	total	26	100	39	100	2	100	4.9
Transformed	correct	10	37	20	38.5	1	16.7	4.8
	intuitive	16	59.3	29	55.8	5	83.3	14.7
	other	1	3.7	3	5.8	0	0	0
	total	27	100	52	100	6	100	10.3

In summary, these results confirm the relationship between successful attention checks and cRT performance. Given the concerns discussed at the beginning of this subsection, a potential recommendation for future CRT research should be to use attention checks with care. Some researchers might prefer to use ACs as control variables, while keeping all participants in their samples. At the same time, this can lead into conflicts with gaols of maintaining the quality of the participant pool, discouraging survey satisficing, and efficiency. At the very least, due to current platform dynamics, the simulataneous presentation of filtered and unfiltered results would be indicated.

2.2 Study 2

2.2.1 Response times and response categories

The consideration of response types, allows for an item analysis parallel to the one in Study 1 for the CRT and CRTt (see Figure S10). Again, data for the Qualtrics sample were added as a reference point.

For all items of the CRT, the correct response was given faster than the intuitive response, while the opposite was true for the CRTt items and the CRT items on Qualtrics. Differences for the second and third CRTt item were smaller than those observed for the respective CRT items on Qualtrics, though. Differences in response times on MTurk between the variants were larger for correct responses than for intuitive responses. Responses on Qualtrics were much slower on average than on MTurk, for all categories.

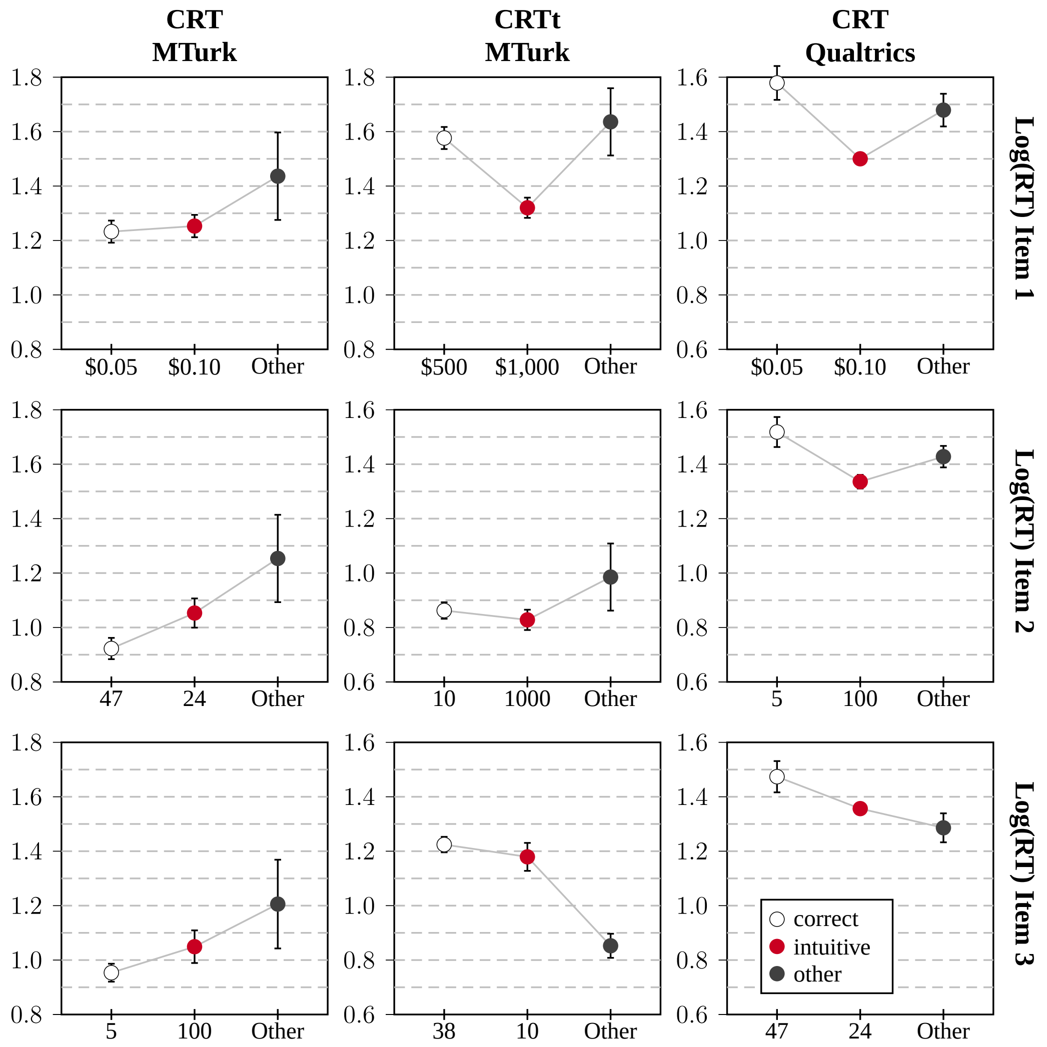

Figure S10. Relationship between response categories and response time in Study 2: Plots shows average values of the log-transformed response time for the three items (rows) in their original version (CRT, left column) and transformed variant (CRTt, right column); whiskers correspond to the 95% CI of the mean or proportion.

The observed differences were consistent with a familiarity account: Participants who remembered the correct (or incorrect) answer were faster than those who did not. More participants on MTurk were familiar with the original items. After transformation, solutions required taking into account the changed numbers. The calculations necessary for the correct answer took up more time than identifying the intuitive solution. In contrast, Qualtrics participants seemed to go through the full process of making sense of the problem structure before starting their calculations.

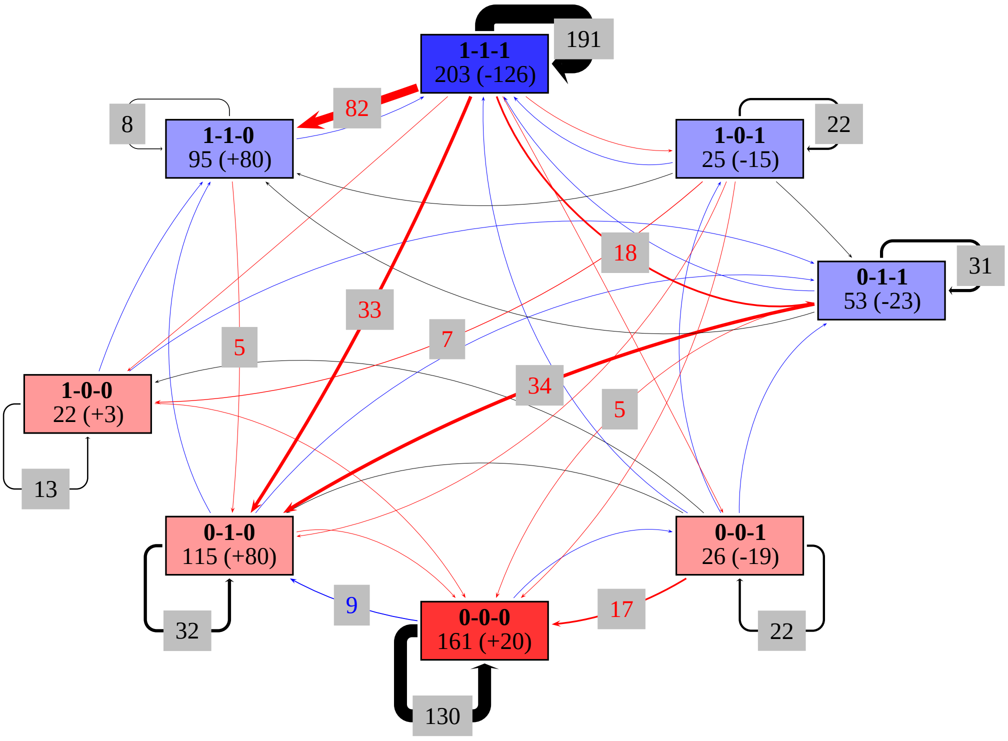

Figure S11. Change in answer patterns in Study 2 from CRT to CRTt: each box corresponds to one of the eight possible answer pattern defined by a three-digit code based on correct (1) and false (0) responses to the three items. Arrows connect the patterns for CRT and CRTt (end point), arrow width corresponds to the number of participants sharing the connection (captions are suppressed below 5). Boxes contain the sample sizes for CRTt score patterns and changes from the sample sizes for the CRT score.

2.2.2 Individual-level relationship between test scores

When treating responses as either correct or false, there are eight possible answer patterns for each test. Figure S11 summarizes the individual relationship between the two individual response patterns, by connecting CRT and CRTt responses. The high correlation between the two tests was mainly produced by two relatively large groups with extreme values, those answering all six items correctly and those answering none of the items correctly (these groups together constituted nearly half of the sample). Second, there are few changes towards better scores in the CRTt.

2.3 Study 3

2.3.1 Results for equivalent items

Figure S12 shows the results for equivalent variants in Study 3. Variants of item 1 are presented in one diagram with the results for the original item 1, and likewise for variants of item 2. For item 1, correct and intuitive responses were given with similar frequency. For the transformed variants, intuitive responses were slightly more frequent, while correct responses were less frequent . This is most pronounced for the variant with transformations of both story and numbers. The response times for correct solutions similarly increased with distance from the original variant, with the transformation of numbers having a larger effect on response times than the change of storyline. Intuitive responses were only slightly slower for transformed items. The gap in panel tenure for participants with correct and intuitive responses was most

pronounced for the original item and virtually non-existent for the doubly transformed item. The pattern for first-time exposure was consistent with the pattern for tenure.

The analysis for item 2 demonstrates that the chosen transformation made the item less difficult. This is most likely due to the deviation from the transformation rules established for Study 2. The constant ratio between the two number for production and time units alone did not suffice to elicit the intuitive response of transforming the production units. In other words, the attempted strategy for increasing the gap between correct and intuitive responses for item 2 backfired. On the other hand, consistent with expectations the social media versions of the item were slightly more difficult than the items using the original storyline. While transformed items were less difficult, they took participants a longer time to solve than the original item, both for correct and intuitive responses. Intuitive responses were not given faster than correct responses. As for item 1, the observed gap in panel tenure for participants with correct and intuitive responses was smaller after transformation, although this effect was restricted to the numerical transformation.

Comparing the results in Study 2 and Study 3, transformations successfully shifted the results for item 1 in the opposite direction of the practice effect associated with panel tenure. In both studies, transformations were less successful for item 2, the difficulty was even lowered after using transformations that deviated from the rules specified in the SM.

Independent of the chosen item set, participants were only asked about prior exposure to the original item in contrast to Study 1. Figure S12 includes the results for prior exposure, which are in line with the results for panel tenure.

2.3.2 Results for non-equivalent variants in Study 3

Figure S13 shows the results for non-equivalent variants (and the original two items for benchmarking). For item 1, the increase in the number of balls remained seemingly unnoticed by the majority of participants who answered with correct and intuitive responses for the original problem. Conforming to the theory that superficial similarities can hinder performance, the change in storyline made this confusion less likely. The reduction in original responses was more pronounced for participants with correct answers ("5" was answered less frequently, "2" was answered more frequently for A4 than for B4). In terms of response times, original responses were given faster than aligned responses with little differences between correct and intuitive responses. Changing the storyline led to a pronounced increase in response times. The gap for correct and intuitive respondents in panel tenure for the original item was replicated for misaligned original responses in both variants. Participants who gave aligned responses had a lower HIT average than participants who gave the respective mismatched responses. Again, the proportions of first-time respondents (regarding the bat-and-ball problem) were consistent with the pattern found for panel tenure (taking into account that first-time exposure becomes more unlikely with increased panel tenure).

For item 2, participants who responded with an unchanged number of production units were coded as giving the correct solution to the original problem. Up to 14.6% of participants (WI) chose this answer, again at lower frequencies after transformation of the storyline. The inverse items (WI, SI) were more likely to evoke this response than WQ and SQ. As the principle for the intuitive response did not change for these four variants, the frequency of intuitive responses was at similar levels for the variants as for the original (with an increase for the inverse variants). As a consequence, the frequency of correct responses was lower for the variants than for the original. As for the first item, the change in storyline led to a relatively higher rate of correct responses (but the difference was smaller fore the second item). The groups of

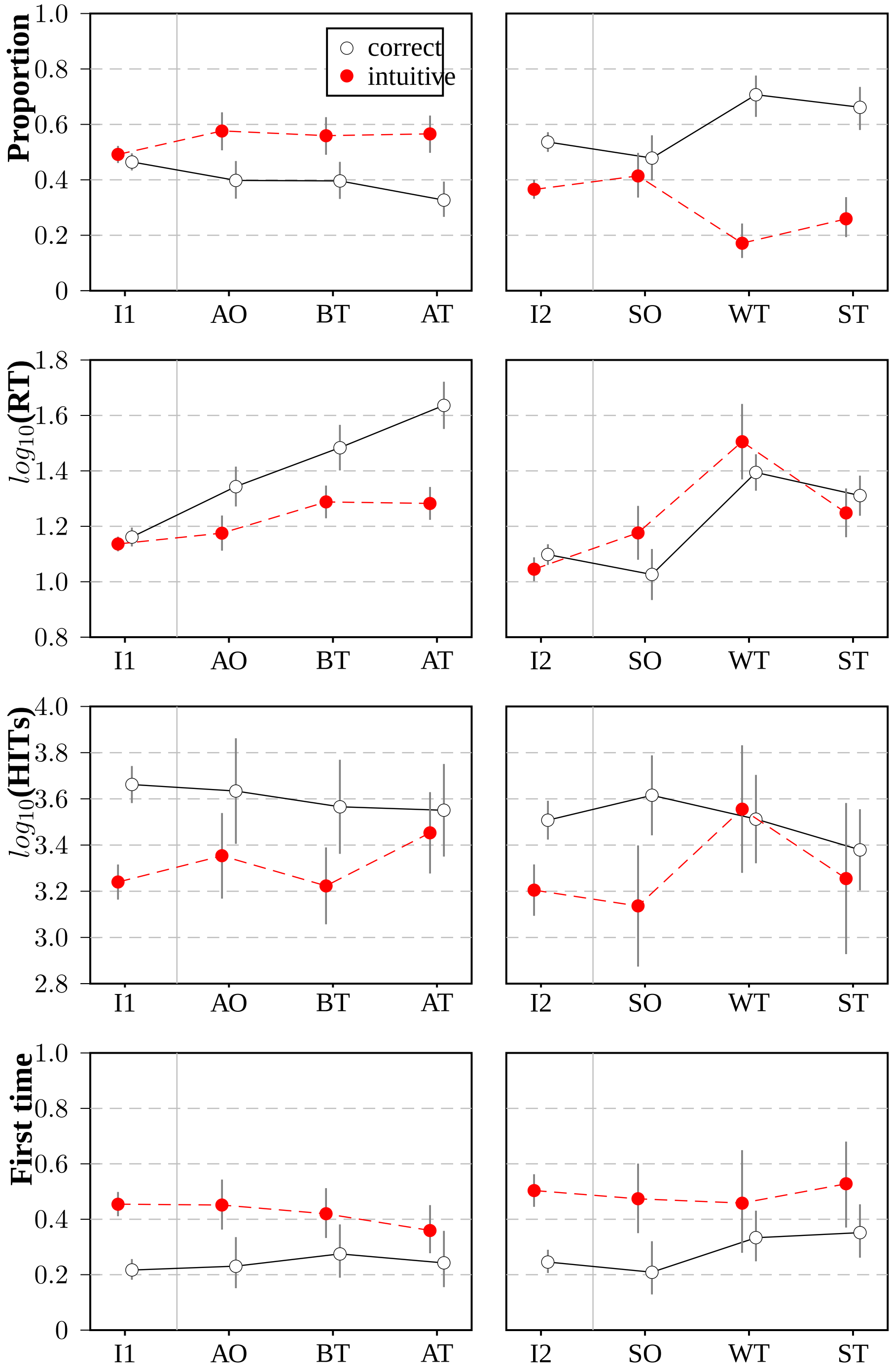

Figure S12. Results for item 1 and its three equivalent variants (left side) and for item 2 and its three equivalent variants (right side) in Study 3. Subfigures show the proportion of intuitive and correct responses (top row), average logarithmized response times for response types (second row), average logarithmized number of HITs (third row), and average proportion of first-time encounters with the ball-and-bat problem for each response type (bottom row). Bars represent 95%-CIs for proportions and means, respectively.

participants with mismatched responses to the original problem gave these response much faster than others, indicating the use of memorized solution strategies. They were also characterized by a longer panel

tenure and smaller proportions of first-time exposure. Correct answers took more time than intuitive answers for the variants, especially for the two inverse variants. Panel tenure was similar, first-time exposure slightly lower for correct respondents.

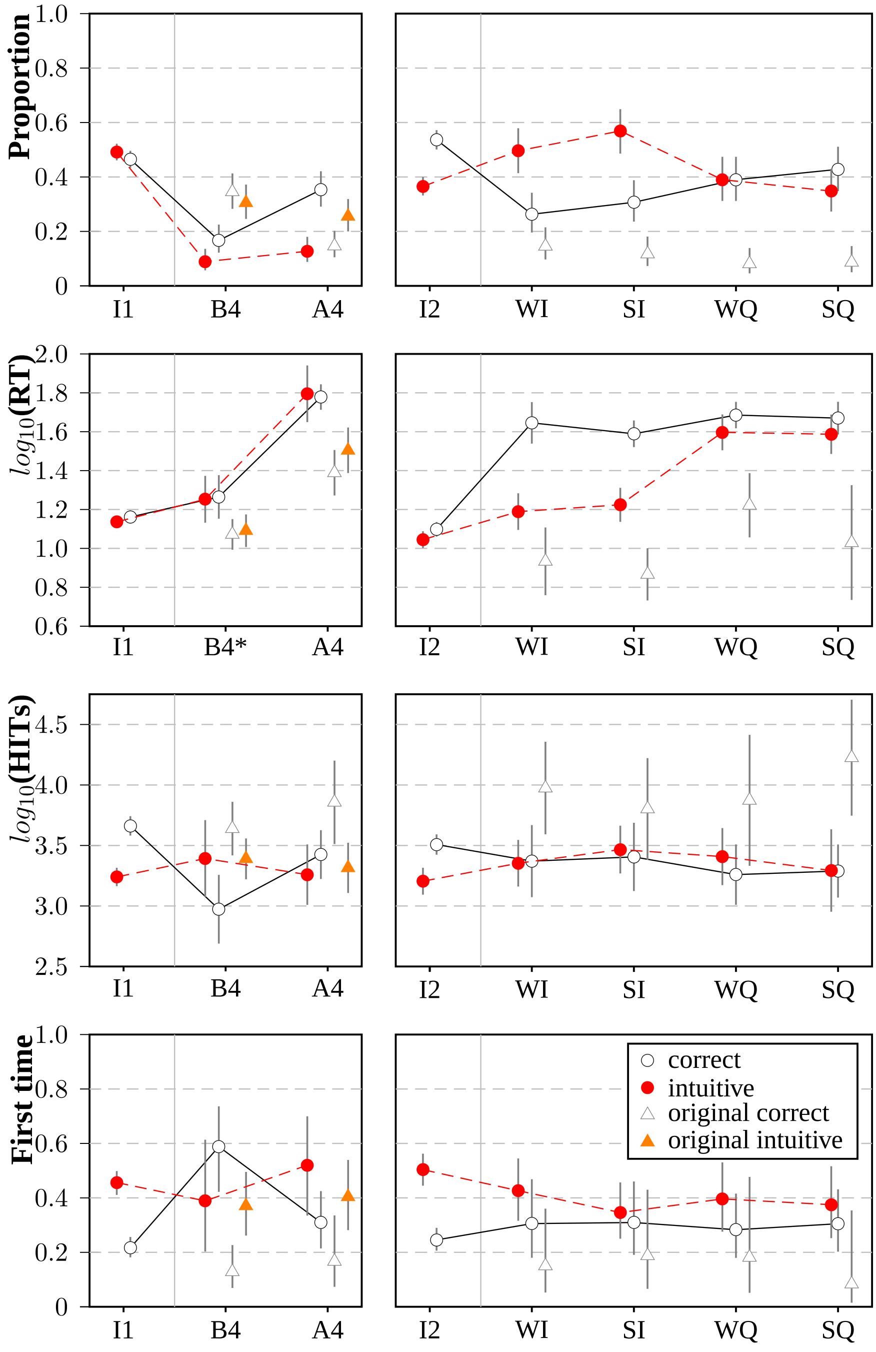

Figure S13. Results for item 1 and its two non-equivalent variants (left side) and for item 2 and its four non-equivalent variants (right side) in Study 3. Responses were categorized as correct or intuitive for the presented item or—for variants—also as correct (in terms of number or applied principle) or intuitive for the original item. Subfigures show the proportion of response types (top row), average logarithmized response times for response types (second row), average logarithmized number of HITs (third row), and average proportion of first-time encounters with the ball-and-bat problem for each response type (bottom row). Bars represent 95%-CIs for proportions and means, respectively.

2.3.3 Extended Results for novel items: prior exposure

Figure S14 includes the results for prior exposure, which, again, are in line with the results for panel tenure.

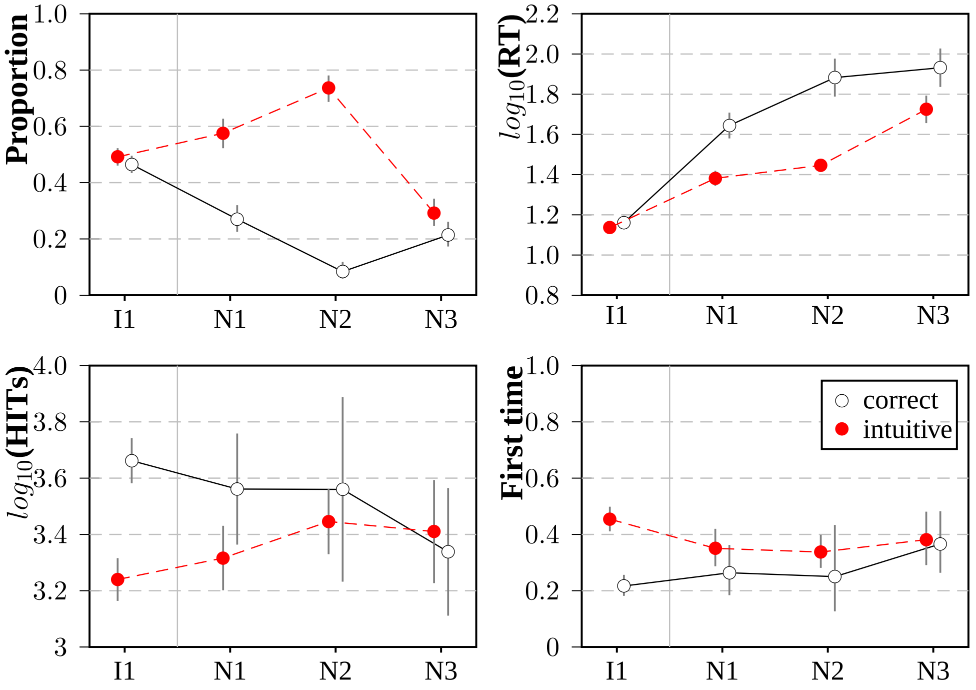

Figure S14. Results for item 1 and the three novel items in Study 3. Subfigures show the proportion of intuitive and correct responses (top left), average logarithmized response times for response types (top right), average logarithmized number of HITs (bottom left), and average proportion of first-time encounters with the ball-and-bat problem for each response type (bottom right). Bars represent 95%-CIs for proportions and means, respectively.

Item N3 was not analyzed in the main manuscript, as it does not constitute a lure item. The item was potentially too difficult, as most responses fell into the non-coded category. Similar to the other novel items, N3 took participants much longer to answer irrespective of answer type, with correct answers taking the longest. In contrast to the other items, there were no gaps in panel tenure for N3.

2.3.4 Extended validity tables for Study 3

Extended Table S7 presents proportions and differences in proportions for respondents with correct and false solutions for each item in Study 3. Gender difference were relatively larger for AT, but not for BT (with a smaller sample size than in Study 1, as evidenced by the size of the CI). Average differences regarding the AC error were higher for the transformed variants for both items (again, with the exception of BT).

Extended Table S8 presents means and mean differences in EV-scale and CRT-score for respondents with correct and false solutions for each item in Study 3. The difference regarding the EV-scale tended to be larger for all transformed equivalent items (with comparable levels for AO and ST). B4 shows a different pattern from the other items, as means are higher for participants with incorrect solutions.

Table S7. Relative number of male participants and attention check errors: Proportions, differences in proportion and CIs for differences in proportions split by correct and false solutions for each of the item variants in Study 3

	Gender				AC Error			
	p_{inc}	p_{cor}	Δ	$CI\ \Delta$	p_{inc}	p_{cor}	Δ	$CI\ \Delta$
CRT1	47.1%	60.5%	13.4%	[7.2%; 19.4%]	15.6%	8.4%	-7.3%	[-11.2%; -3.2%]
AO	50.0%	62.8%	12.8%	[-1.4%; 26.1%]	15.3%	6.4%	-8.8%	[-17.2%; 0.6%]
BT	59.8%	72.5%	12.7%	[-0.8%; 25.0%]	13.9%	12.5%	-1.4%	[-10.6%; 8.9%]
AT	49.3%	70.1%	20.9%	[6.5%; 33.5%]	15.2%	6.0%	-9.2%	[-17.1%; 0.6%]
B4	42.6%	50.0%	7.4%	[-10.2%; 24.9%]	14.2%	5.9%	-8.3%	[-15.7%; 5.6%]
A4	46.2%	45.8%	-0.4%	[-14.3%; 13.7%]	12.9%	9.7%	-3.2%	[-11.5%; 7%]
CRT2	41.3%	60.9%	19.6%	[12.5%; 26.5%]	14.0%	9.7%	-4.4%	[-9.1%; 0.2%]
SO	49.3%	73.1%	23.8%	[7.6%; 38.2%]	19.2%	7.5%	-11.7%	[-23%; -0.2%]
WT	58.5%	71.7%	13.2%	[-3.5%; 30.3%]	19.5%	11.1%	-8.4%	[-23.7%; 3.7%]
ST	40.4%	52.2%	11.7%	[-5.7%; 27.9%]	21.3%	13.0%	-8.2%	[-22.9%; 4.3%]
WQ	48.2%	62.3%	14.1%	[-3.1%; 29.8%]	9.6%	11.3%	1.7%	[-8.5%; 13.9%]
SQ	45.6%	57.6%	12.1%	[-4.7%; 27.8%]	7.6%	10.2%	2.6%	[-7.1%; 13.6%]
WI	41.6%	66.7%	25.1%	[6.1%; 41.1%]	21.8%	2.8%	-19.0%	[-28.3%; -5.7%]
SI	38.1%	69.0%	30.9%	[12.8%; 45.8%]	12.4%	4.8%	-7.6%	[-16.3%; 4.6%]
N1	48.8%	56.0%	7.3%	[-4.7%; 18.8%]	11.0%	8.8%	-2.2%	[-8.4%; 6.1%]
N2	52.0%	78.6%	26.6%	[-5.8%; 44.4%]	14.4%	14.3%	-0.1%	[-9.7%; 17.5%]
N3	49.0%	77.5%	28.4%	[15.9%; 38.6%]	14.2%	4.2%	-10.0%	[-15.5%; -1.6%]

These results partially replicated the findings in Study 2 that transformations can be beneficial for the validity of the CRT as predictor, although the relatively small sample sizes for variants did not allow for a definitive determination. Note that respondents with correct and incorrect answers to N3 differed markedly in EV-scale values, CRT values, gender, and AC errors.

Table S8. EV-scale scores and performance on original items: Means, mean differences and CIs for the mean difference split by correct and false solutions for each of the item variants in Study 3.

	EV-scale				CRT (1+2)			
	M_{inc}	M_{cor}	Δ	$CI\ \Delta$	M_{inc}	M_{cor}	Δ	$CI\ \Delta$
CRT1	1.49	1.97	0.48	[0.36; 0.59]	*0.33*	*1.79*	*1.47*	*[1.40; 1.53]*
AO	1.55	1.99	0.44	[0.17; 0.70]	0.62	1.82	1.20	[1.02; 1.39]
BT	1.45	2.03	0.58	[0.32; 0.83]	0.38	1.80	1.42	[1.26; 1.58]
AT	1.63	2.14	0.51	[0.24; 0.78]	0.65	1.80	1.15	[0.96; 1.34]
B4	1.75	1.62	-0.13	[-0.53; 0.27]	0.90	0.81	-0.09	[-0.48; 0.3]
A4	1.63	1.70	0.07	[-0.22; 0.37]	0.82	1.34	0.52	[0.25; 0.78]
CRT2	1.51	1.88	0.37	[0.23; 0.50]	*0.20*	*1.67*	*1.46*	*[1.40; 1.53]*
SO	1.44	1.96	0.51	[0.19; 0.83]	0.30	1.70	1.40	[1.21; 1.59]
WT	1.39	2.04	0.65	[0.33; 0.97]	0.29	1.49	1.20	[0.96; 1.45]
ST	1.53	1.88	0.35	[0.02; 0.67]	0.40	1.15	0.75	[0.45; 1.05]
WQ	1.64	1.81	0.17	[-0.18; 0.52]	0.80	1.21	0.40	[0.05; 0.75]
SQ	1.41	2.00	0.59	[0.25; 0.94]	0.89	1.17	0.28	[-0.04; 0.60]
WI	1.52	1.97	0.45	[0.06; 0.84]	0.69	1.63	0.94	[0.63; 1.24]
SI	1.63	1.98	0.35	[0.04; 0.66]	0.83	1.42	0.60	[0.26; 0.94]
N1	1.62	1.79	0.17	[-0.06; 0.41]	0.88	1.51	0.62	[0.40; 0.84]
N2	1.71	2.00	0.29	[-0.06; 0.64]	0.92	1.86	0.95	[0.72; 1.18]
N3	1.62	2.21	0.59	[0.36; 0.83]	0.77	1.45	0.68	[0.44; 0.92]

2.3.5 Extended Confusion matrices

Figure S15 shows the full range of confusion matrices in Study 3, including the ones presented in the manuscript.

I1 \ AO	1	0
1	40	12
0	0	48

$n = 196$

I1 \ BT	1	0
1	39	5
0	1	55

$n = 201$

I1 \ AT	1	0
1	31	16
0	2	51

$n = 202$

I1 \ I2	1	0
1	36	9
0	18	37

$n = 751$

I2 \ SO	1	0
1	45	4
0	0	51

$n = 104$

I2 \ WT	1	0
1	60	6
0	11	24

$n = 104$

I2 \ ST	1	0
1	47	5
0	19	29

$n = 103$

I1 \ B4	1	0
1	6	34
0	11	49

$n = 203$

I1 \ A4	1	0
1	27	23
0	8	41

$n = 201$

I2 \ WI	1	0
1	23	23
0	4	51

$n = 102$

I2 \ SI	1	0
1	25	28
0	7	39

$n = 102$

I2 \ WQ	1	0
1	26	27
0	13	34

$n = 100$

I2 \ SQ	1	0
1	27	28
0	19	25

$n = 102$

I1 \ N1	1	0
1	19	29
0	8	44

$n = 337$

I1 \ N2	1	0
1	7	40
0	1	52

$n = 334$

I1 \ N3	1	0
1	14	30
0	7	48

$n = 332$

I2 \ N1	1	0
1	20	37
0	6	38

$n = 255$

I2 \ N2	1	0
1	8	46
0	0	46

$n = 252$

I2 \ N3	1	0
1	16	34
0	5	45

$n = 244$

Figure S15. Cross-tabulation (including non-equivalent items) of correct and false responses for original items and variants (showing rounded percentages) in Study 3. Improvements from original to variant are captured in the lower left corner (in blue), worse performance in the upper right corner (in red).

2.3.6 Alternate Confusion matrices

Figure S16 shows alternative co-occurrence matrices for Study 3. In each matrix, scores for item variants are cross-tabulated with the score on the original item that was not the source for the variant. As the solution rates for bat-ball items is generally lower as for the wicket-production items, many variants of I2 show improvements compared to I1, while variants of I1 are solved at a lower rate than I2. Nonetheless, for each item pair between 50% and 65% have the same score on both items.

	AO 1	AO 0
I2 1	33	27
I2 0	7	33

$n = 144$

	BT 1	BT 0
I2 1	33	18
I2 0	7	42

$n = 150$

	AT 1	AT 0
I2 1	26	31
I2 0	5	37

$n = 155$

	SO 1	SO 0
I1 1	34	12
I1 0	14	40

$n = 139$

	WT 1	WT 0
I1 1	47	4
I1 0	24	25

$n = 140$

	ST 1	ST 0
I1 1	34	7
I1 0	32	27

$n = 138$

	B4 1	B4 0
I2 1	8	41
I2 0	9	41

$n = 152$

	A4 1	A4 0
I2 1	20	30
I2 0	13	37

$n = 152$

	WI 1	WI 0
I1 1	20	28
I1 0	7	45

$n = 137$

	SI 1	SI 0
I1 1	20	27
I1 0	10	42

$n = 139$

	WQ 1	WQ 0
I1 1	22	22
I1 0	17	39

$n = 136$

	SQ 1	SQ 0
I1 1	26	23
I1 0	17	34

$n = 138$

Figure S16. Alternative co-occurence matrices for item solutions in Study 3: Each matrix tabulates the rounded percentage of cases with each possible combination of item scores for one item pair.

2.3.7 Memorization strategies and performance

As noted in the manuscript, participants who indicated that they had memorized the answer to the bat-and-ball problem performed slightly better on the problem than those who indicated that they had memorized the procedure of calculating the answer (63% vs. 56.6%; $d_{a-p} = 6.4\%$; 95% CI=[-2.2%, 14.7%]). Both groups performed better than participants who had memorized neither (27.5%). This does

not answer the question, though, how the performance of these groups would generalize to novel items and variants of the problem.

Obviously, participants with each type of memorization strategy cannot be considered as a homogeneous group. Those participants who gave the correct and those that gave the intuitive answer to the bat-and-ball problem will have memorized different answers and procedures. Therefore, to estimate the generalizability of their performance, the sample was split by memorization response and answer to the original item. The performance of these subgroups for relevant other items in Study 3 is shown in Table S9. Note that due the studfy design, participants only answered a subset of items.

Table S9. Performance for alternative items based on response for the bat-and-ball problem and self-reported memorization strategies: The sample is restricted to participants who indicated that they had seen the bat-and-ball problem before.

		bat-and-ball (I1) correct						bat-and-ball (I1) intuitive					
		answer		procedure		none		answer		procedure		none	
Item	Response	N	perc	N	perc	N	perc	N	perc	N	perc	N	perc
AO	**correct**	20	**80**	39	**79.6**	1	**50**	0	**0**	0	**0**	0	**0**
	intuitive	5	20	8	16.3	1	50	4	100	33	100	7	100
	other	0	0	2	4.1	0	0	0	0	0	0	0	0
BT	**correct**	18	**78.3**	35	**97.2**	3	**75.0**	0	**0**	2	**5.3**	0	**0**
	intuitive	4	17.4	0	0	1	25	12	100	36	94.7	11	91.7
	other	1	4.3	1	2.8	0	0	0	0	0	0	1	8.3
AT	**correct**	10	**55.6**	37	**64.9**	2	**100**	0	**0**	1	**2.9**	0	**0**
	intuitive	8	44.4	11	19.3	0	0	9	81.8	32	94.1	10	100
	other	0	0	9	15.8	0	0	2	18.2	1	2.9	0	0
N1	**correct**	8	**24.2**	38	**45.2**	2	**25**	2	**11.1**	12	**20.3**	2	**12.5**
	intuitive	19	57.6	36	42.9	4	50	15	83.3	33	55.9	11	68.8
	other	6	18.2	10	11.9	2	25	1	5.6	14	23.7	3	18.8
N2	**correct**	4	**10.3**	15	**20**	0	**0**	1	**3.6**	0	**0**	1	**6.7**
	intuitive	31	79.5	48	64	3	100	24	85.7	41	85.4	10	66.7
	other	4	10.3	12	16	0	0	3	10.7	7	14.6	4	26.7
N3	**correct**	9	**21.4**	26	**35.6**	2	**28.6**	2	**15.4**	4	**6.3**	2	**25**
	(intuitive)	4	9.5	20	27.4	1	14.3	6	46.2	24	38.1	2	25
	other	29	69	27	37	4	57.1	5	38.5	35	55.6	4	50

Analyzing these results, I focus on correct solution rates between groups. It is evident at first glance, that participants who did not solve the original item, perform much worse on all item variants presented in the Table (consistent with the results presented above). The subgroups of participants who answered the original item correctly, but had no memorization strategy are very small and difficult to interpet. A relevant comparison can be amde between participants who memorized (the correct) answer and the (most likely) correct procedure.

Both groups perform similar regarding item AO ($d_{a-p} = 0.4\%$; 95% CI=[-20.7%, 17.7%]): When only the item text changes, both answers and procedures remain valid. For both transformed items BT and AT, the group that memorized the procedure performed better than the group that memorized the answer. This difference is more pronounced for the transformed original item BT ($d_{a-p} = -19.0\%$; 95% CI=[-39.3%, -2.4%]) than for the item with novel item text ($d_{a-p} = -9,4\%$; 95% CI=[-33.8%, 14.4%]).

Similar differences exist for the three novel items. Participants who memorized procedures outperformed the group who memorized the answer in responses to the first novel item N1 ($d_{a-p} = -20.1\%$; 95% CI=[-36.6%, -1.4%]) and to a lower degree in responses to N2 (N2 $d_{a-p} = -9.7\%$; 95% CI=[-21.9%,

5.5%]) and to the non-lure item N3 ($d_{a-p} = -14.2\%$; 95% CI=[-29.2%, 3.4%]). Thus across items, the pattern is consistent, showing an advantage for participants who memorized the procedure. In this sense, the comparison of responses to the original item yields an incorrect representation of the relative strengths.

Regarding items not shown in the Table and less relevant for the comparison, the group memorizing answers performed slightly better regarding item A4 and SQ (but slightly worse for B4 and WQ)worse for SI, WI, and SO and similarly for WT and ST.

2.4 Classification of open answers in Study 3

Responses to the open questions were coded using the categorization scheme shown in Table S10. Categories were non-exclusive. Table S11 shows the proportion of answers falling into categories split by self-reported previous exposure to the bat-and-ball item. Finally, Table S12 lists the solution rates for participants with and without previous exposure whose responses were coded into each category (participants can be analyzed in more than one category).

Some direct quotes from answers by repeat participants may help to flesh out some of the categories. Participants who felt the questions were overused called them directly this or a “boiler plate question”, “outdated”, “recycled”, “too prevalent”, a “cliche question”, “largely useless”, a “daily question” or “a waste of time” (see the SM for a list of statements in context). A number of them expressed the explicit wish to see new or updated questions. Many expected to be asked the third item of the CRT: “you haven’t asked me about lilypads yet but I assume that’s coming”. Some subjective theories about the scale coincided with the measured construct, others moved to intelligence and one participant thought the items could “determine one’s belief in God”. Some participants described how they discovered the correct solution after several failed attempts on earlier HITs. Some admitted to looking the solutions up at some point (“Since then, I have known the answer to the question”); others stated that they were never motivated enough to do so. There were single instances of participants declaring that feedback after a HIT led them to the correct answer, one person worked through the problem with her daughter, another single participants admitted to knowing the correct answer without having any idea why it was correct (“i know the answer is 5 but no idea how it is that number”). A few participants openly worried that memorization could decrease the utility of the test and recommended changing the question. These open answers are instructive as they help to identify both sources of annoyance for frequent participants and test-taking strategies that undercut researchers’ intention.[9]

2.4.1 Exemplary Answers: First time

[*All statements are presented as typed.*]

1. It’s a tricky one because your brain just wants to go with the easy answer, you have to backup and make sure your logic is correct.
2. They were all very simple and required almost no thought.
3. The bat and ball seemed simplistic so maybe I’m missing something
4. I don’t really have an opinion about the question. It is a pretty easy word problem.
5. Pretty simple arithmetic, shouldn’t require a search lol

[9] A nice example is a reference to the attention check proposed in Oppenheimer et al. (2009). A participant explained that due to the frequent reuse of the original question, most Turkers might have adapted to it: “As soon as you see the word vacuum you know it’s an attention check. So does that really check your attention?”. Milland (2015) notes that some Turkers use browser add-ons to highlight words used in attention checks.

Table S10. Categorization scheme for open answers in Study 3

Type	Category	Description
1. Incorrect beliefs	1.1 Attention check	Participant believes that task is an attention check.
	1.2 Puzzlement	Participant wonders why a simple task is presented.
	1.3 Simple Problem (error)	Participant describes the task as simple (and misses the trick).
	1.4 Simple Problem (unclear)	Participant describes the task as simple (and might have missed the trick).
2. Correct Understanding	2.1 Explanation of correct answer	Participants explains how the correct solution is calculated.
	2.2 Other CRT items	Participant lists at least one other CRT item.
	2.3 Similar to tasks in the HIT	Participant points out similarities between bat-and-ball item and variants.
	2.4 Suspects Trick	Participants suspects trick, but is not able to identify it
	2.5 Explains Trick	Participant explains why many participants will give a wrong answer.
	2.6 Second thought	Participant declares the item to be tricky and to require thought.
3. Preference	3.1 Like	Participant likes question (and/or similar questions).
	3.2 Dislike	Participant dislikes question (and/or similar questions).
	3.3 Neutral	Participant expresses to feel neutral about the question.
4. Other	4.1 Discovered solution	Participant explains how the solution was discovered (with foreign help).
	4.2. Overused question	Participant expresses that the question is asked too often on Mturk.
	4.3 Unrealistic	Participant complains about chosen numbers or plausibility of item.
	4.4 Would like to know answer	Participant would like to know the solution.
	4.5. No challenge	Participant expresses that the question holds no challenge after discovering the solution.
	4.6 Test	Participant explains what the question measures.

Table S11. Proportion of participants with and without previous exposure whose answers were categorized into each category in Study 3

		Proportion (%)	
Type	Category	Seen before	Not seen before
1. Incorrect beliefs	1.1 Attention check	6.7	1.0
	1.2 Puzzlement	4.6	–
	1.3 Simple Problem (error)	4.7	13.1
	1.4 Simple Problem (unclear)	16.7	20.7
2. Correct Understanding	2.1 Explanation of correct answer	0.6	0.6
	2.2 Other CRT items	10.7	0.3
	2.3 Similar to tasks in the HIT	4.6	9.9
	2.4 Suspects Trick	5.8	13.4
	2.5 Explains Trick	1.4	0.3
	2.6 Second thought	10.8	11.1
3. Preference	3.1 Like	12.6	7.0
	3.2 Dislike	6.5	5.1
	3.3 Neutral	11.0	8.6
4. Other	4.1 Discovered solution	1.2	–
	4.2. Overused question	7.6	–
	4.3 Unrealistic	0.2	0.6
	4.4 Would like to know answer	1.2	–
	4.5. No challenge	4.3	–
	4.6 Test	5.3	1.9

Table S12. Solution rates for participants with and without previous exposure whose answers were categorized into each category in Study 3

		Correct (%)	
Type	Category	Seen before	Not seen before
1. Incorrect beliefs	1.1 Attention check	25	0
	1.2 Puzzlement	30	-
	1.3 Simple Problem (error)	13	2
	1.4 Simple Problem (unclear)	46	31
2. Correct Understanding	2.1 Explanation of correct answer	50	100
	2.2 Other CRT items	70	0
	2.3 Similar to tasks in the HIT	73	52
	2.4 Suspects Trick	47	7
	2.5 Explains Trick	89	100
	2.6 Second thought	90	66
3. Preference	3.1 Like	75	50
	3.2 Dislike	54	19
	3.3 Neutral	53	22
4. Other	4.1 Discovered solution	88	-
	4.2. Overused question	70	-
	4.3 Unrealistic	100	0
	4.4 Would like to know answer	13	-
	4.5. No challenge	96	-
	4.6 Test	40	33

6. It just needs a little thought. It would be easy to assume the items cost $1 and $.10 but then the statement given would not be true
7. Those are probably tricker than I made them to be.
8. it was basically the same as the age question. even the numbers. so i already had the answer.
9. It is just trying to trick me into giving an obvious sounding answer instead of thinking about it.
10. I am not sure what you are asking. Was the bat and ball question a trick?
11. I mean it was basic subtraction. Unless I'm missing something.
12. Im not sure if it was a trick question or not but I answered with my gut instinct
13. I think it's a smart question and requires one to do a little simple math because the numbers initially direct you the wrong way
14. I'm not clear on why it is a big deal. It is simple math. The bat is 1 dollar more which leaves 10 cents unaccounted for. no, I don't feel similar about the other questions or tasks
15. This is to judge how swiftly one can make snap mathematical decisions regarding subtraction/addition.
16. I thought it was odd that someone would actually buy a gold ball and bat.
17. The one questions about the computers created widgets and the age question were both familiar. They all seemed like math problems from my daughters homework.
18. It feels kind of silly, which is making me nervous about my answers. Wondering if it was more than addition/subtraction.
19. These are all "Iq" test questions that rely on how well a person reads the question
20. I think it's a trick question that looks simple but isn't. I dislike trick questions, I have no patience for them.
21. I hate math word problems.

2.4.2 Exemplary Answers: Repeated exposure

[*All statements are presented as typed.*]

1. Although it seems like an easy math problem, it does take a little thought to answer correctly
2. It's often used like the lily pads that double every day scenario. I'm not sure what it tests, but I know it tests something.
3. I think the bat and ball question is pretty simple. If the total for both is $1.10, and the bat is worth $1.00, that leaves a balance on $.10.
4. I feel it is used too often in these surveys. If you want to calculate someone's math ability the questions should be more diverse and change. The question about the pond also comes up a lot.
5. No opinion just a common answer to test if people are close readers and problem solvers
6. It's a pretty simple question if you think about it for a couple of seconds. Not difficult at all.
7. I feel it is kind of a trick question. Most people would probably respond that it cost .10 when it really only cost .05.
8. It always seems so easy that I wonder if there is a trick or if I'm missing it in a stupid manner. It just seems toooo easy.
9. its fine it keeps my brain working. I like the half life of the lake question too.
10. It's odd, but very popular with requesters so I'm used to it.
11. It made me go back in time to elementary school word problems in math class. I felt tense.
12. It's a bit overused, but tricky to solve the first time.
13. I think this is the first time I got the answer right on this question. Last time I saw this, I think I answered that the ball cost 10 cents, but now I realize that was not correct. I like working math problems that challenge my brain a little.
14. It's so common a question that I'm not sure it's effective at measuring whatever it is trying to measure. I feel the same way about the widget question.
15. It is to see if you can add.
16. It seems relatively simple. It seems to rely on a gut reaction to get it wrong. Upon first glance the number $0.10 jumps out at your but that is just the hippocampus associating the numbers $1.10 and $1.00 in the most usual way when considering a difference (subtraction). You need to spend a moment with the question to allow it to route the the prefrontal cortex for actual consideration if you want to get the answer right due to the qualifier. The other questions asked in this section (4 in total) are all similar in nature in that they rely on the deceptive nature of the brain's first impressions.
17. I was confused the first time I got the question so I looked it up after completing the survey. Since then, I have known the answer to the question.
18. It feels like an attention check.
19. I dislike it in general. I'm not sure of it's purpose but it feels a bit useless in surveys.
20. It is fairly simple once you do it a few times.
21. I feel that the whole bat & ball, widget, and the pond covering questions are a waste of time.
22. It's supposed to determine one's belief in God.

23. The ball-and-bat question and the moss covering the pond question have popped up quite a few times recently with mTurk HITs–was always curious why. Personally I researched them after seeing the questions at the end of surveys and was wondering what the cause was.
24. My opinion is that it is a boiler plate question that is used in an attempt to identify bots. There are several other questions that are commonly asked together with this one. An example being a lake with lily pads that double each day.
25. It is a fun trick question cuz i know i got it wrong the first time i encountered it but i still dont know if its right.
26. I have seen it a couple times. I still can't figure it out, but I know the answer is not 10. Its similar, but I have never heard the one about the grandfather being 100 years older the his grand kids. I couldn't figure that on out at all.
27. I don't understand why it is asked so often and I would like feedback on my answer.
28. I have no opinion on the question, I just think it's an attention check question.
29. The question is too easy but fair for studies of this type. I feel the same for a lot of survey questions even unrelated to Mturk.
30. Its very tricky, and my intuition was wrong the first time i encountered it.
31. I think it's a very overused question. Initially, it can be a bit tricky for people who haven't seen it before. Although, if they just take their time and calculate it and check their work, it should be simple.
32. Where can you buy a bat and ball for $1.10? That's preposterously low.
33. Effective the first time, but after that....
34. I feel that it is a boring question and I don't know why it is asked.
35. I think it's overused, but it's a good one. I explained it to the kids in my 4th/5th grade class.
36. This question is a pretty common comprehension or reading check. I always answer that the ball cost 10 cents. I assume this is right but I have never been told if this is the correct answer. I think it is one of the easiest questions and I often wonder what is the purpose of the question. There is another question that often conincides this one. This is the one were a lily pad is growing on a lake and doubles in size everyday and 48 days to take up the entire lake. The question is how many days would it take for the lily pad to take up half the lake. I assume it takes 47 days.
37. It's okay. I feel that way about all questions. That's part of participating.
38. I feel that it is sort of outdated. A new, fresh problem should be created.
39. It's overly used which makes the question somewhat irrelevant
40. It's an interesting math problem and works against what you intuitively want to do. If you want to know if someone is mathematically inclined then it's a good start. Though it's probably best to not use the same exact problem every time as it's easy to look up and memorize the answer.
41. I feel that it is a basic question that is asked often. I think that people just memorize the answer to it.
42. It's an interesting mind puzzle. Your first bat and ball question with FOUR balls threw me for a minute :-)
43. I think the bat-and-ball question is sort of a trick question. If I am answering incorrectly I would find it humorous that I have answered it the same way about 10 times. I do not know of any questions or tasks that I feel similar about.
44. I am an algebra teacher, so the knowledge of how to work these problems is what I practice on an ongoing basis. It is not a very difficult question. Thank you.

45. I wish that requesters would come up with something new. All the supposed brainteasers have been recycled so much, it's comical.
46. It is too prevalent in surveys. The same goes for the widget production questions. All of the brain teaser questions are asked repeatedly to the point that the survey taker remembers them and are not useful anymore.
47. It's a tricky question! I think I had the wrong answer the first time I answered it, but I kept thinking about it and eventually asked me daughter her opinion on it, and we worked out the correct answer together.
48. All the tasks in this survey in particular were similar to the bat-and-ball question, particularly one of the earlier questions about the grandad and grandson. Initially, you would think the answer would be 70 and 12, but it would be incorrect, because the grandad would only be 58 years older than the grandson. The correct answer there would be 76 and 6.
49. It has become a pretty cliche question on mechanical turk surveys.
50. It is a basic question. Anyone over the age of 8 should get it easily. If I got it wrong this is embarrassing.
51. Used so often I suspect it is largely useless on a platform like mturk. The question about the widgets is the same. Also, the one about lily pads covering a pond.
52. I was answering it incorrectly at first until a HIT told me it was wrong, then I had to rethink my logic.
53. I think it's a good way to measure people's reasoning abilities. But, it seems like it's been used so much, I'd be surprised if there weren't many people on MTurk that haven't seen it at least once before.
54. I think it's overused a good question if someone has never heard it but once they have it is no longer a critical thinking question.
55. The bat-and-ball question is overused in surveys. I feel the same about the media bot question you presented me with. I've seen it before, in several variants. I wonder if you'll ask me about the lilypad that doubles in size next.
56. It's a reasonable math question. I'm not sure what it's supposed to be testing, other than ability to calculate monetary amounts.
57. I think it's a pretty standard question for gauging critical thinking and calculation skills. The other questions regarding x machines for y minutes to produce z product is similar as well. I have encountered it in other forms, but it's not as though I memorize the answers. I actually just solve the problem again.
58. All of those types of questions are tedious. Once you look them up you can memorize the answers. However, if you try and calculate them getting them correct depends on how good you are at math. While I used to teach statistics, that was 20 years ago and now I suck at math, so I detest these types of questions. They could, perhaps, be measuring the wrong thing?
59. I think it's thought provoking. Most people would say the ball costs 10 cents, but a second examination of the facts reveals that this cannot be true. Another question I've encountered before and made me feel similar was a quiz about which person is looking at another. I don't remember the specifics, but I've never gotten the answer right, nor have I really ever understood it.
60. It's just a trick question. People who aren't familiar with it will just get it wrong without thinking, I claim that even some theoretical physicists who don't pay attention will get it wrong, and then go back to doing differential equations. Also, some people who are absolutely terrible at mathematical reasoning, such as myself, who might have the math version of dyslexia, will be able to figure it out if they see it enough times and they become very familiar with it.
61. I wish they'd give me the answer to it for a change.
62. It's a little too ”tried and true,” if you get my drift.

63. The bat-and-ball question is redundant. There are other questions testing aptitude that are also redundant.
64. It seems very obvious, not noteworthy, except as a measure of attention and to verify the legitimacy of the human (not robot) participation.
65. it's boring since everyone seems to use it
66. I don't like it. I never knew if I answered it correctly. I always just guessed at the answer.
67. I think it's annoying. Just like the stupid "how many widgets" and " how many days for lillypads" questions.
68. I feel like it's so commonly used it's not the best research tool anymore.
69. I'm tired of seeing it.
70. No opinion, really. Just another MTURK HIT
71. I always feel like I get this wrong but I have never been told the answer. The question drives me nuts. I have never looked up the answer because I grew up without google and don't google everything.
72. Really overused, there are a few other logic questions (like the lilipad question) that are also often overused.
73. I like the critical thinking questions. I work as a bartender and believe it or not I will hear a new one once in a while and find it pretty interesting.
74. Not sure maybe an attention check for data quality?
75. It is a tedious question with an obvious answer. All it is is basic math. There are many questions like it, and I do not enjoy answering any of them.
76. I have done it so many times that I barely even put thought into it when I see it at this point, I just know right away that it's 5 cents. I remember when I first ever encountered it I used to put 10 cents and then I paid closer attention to what it was asking and realized my own error and have always put 5 cents ever since and now it's become second nature. There are other questions like this that come up. One that comes to mind is about a lily pad on a lake and with it doubling in size, etc.
77. I am aware of a number of attention check and computational questions - however, I am not sure whether the bat and ball question qualifies as an attention check item (I have not seen this one in the survey design research literature, despite being a social science researcher myself).
78. I start with the bat costing $1.00, then increase the price by $0.01 until I get the answer. I don't think it is a difficult question.
79. I am just curious as to why it constantly shows up.
80. I think the bat-and-the-ball question is easy to answer so that people don't feel frustrated during a survey. Some attention check questions can be similar across different surveys and they are easy if one is actually paying attention.
81. I like critical thinking questions and riddles very much and I would that more of them would be used with a larger variety.
82. I think it's an ok question to prevent bots and do attention checks. Aside from those 2 reasons, I'm not really sure what the purpose of it is but I would like to if I could.
83. I think you guys should come up with another question.
84. I think it is just a question to distract from the survey
85. I think I see it enough that people should stop asking it.

86. pond question, if lillies grows exponentially how long will it take for lillies to cover pond if it took 47 days to cover half mushrooms choir overlap[10]
87. Researchers need to work harder to find different critical thinking questions so that regular survey takers aren't working from rote. The earlier question about the number of messages that can be sent was a great twist.
88. It's easy enough. I've seen that one and the 100 widgets one, you haven't asked me about lilypads yet but I assume that's coming.
89. 47 days. Mturk has a lot bigger problems than the widget/bat and ball/lily pond answers. Right now there is a forum selling access to a tool that just blindly accepts every HIT over a certain dollar amount.
90. It's a nice little trick question made to make the reader think that it's 10 cents for the ball. They see $1.00, ignore the other context, and because they know it must add up to $1.10, they chose 10 cents. It's more of a trick question regarding people's ability to fully read and comprehend the subject than it is a math problem.
91. It's a simple enough question that might be able to screen out bots, but it's common enough to be easily programmed in.
92. I think that it is a bit overused and too popular to be useful in research anymore. There are no other questions that I feel this way about,
93. It is annoying because it is a daily question.
94. There are many other similar variants on this question, such as "At a ballgame, a hot dog and a soda cost $5.50 in total..." with the same design, just different elements. I am also very familiar with the widgets question, and questions like "A lilypad doubles in size every day..." or the "sun tea concentration" variant of that question.
95. It's easy. The widget one I've been asked several times and I'm not sure if I'm ever right on that one or not. No one has ever said correct or incorrect and I never cared to google it.
96. A question like this can tell you whether the person thinks intuitively or logically.
97. I feel that it and the usual accompaniments (widgets, expanding lilypad, etc.) are overused and unlikely to provide any useful data.
98. I know my ansswer must be wrong cause it seems to simiple
99. I have no idea if I even answer it correctly, I just answer the same way each time and never thought to look up the correct answer. There's another similar one I'm asked a lot about a pond with lily pads or bread with mold where each day the mold/lily pads double in size until they cover the pond/bread.
100. I'm 66. This is 2nd grade arithmatics.
101. They are redundant and boring. They just take up time in the HIT and take away from the true purpose of the HIT.
102. It's overdone. Try to find some new ones
103. It's a standby question that a lot of requestors go to.
104. I am tired of seeing the bat and ball question, it has been used so many times now. I do not feel as if there are other questions or methods that are as overused as that.
105. It all seems pretty easy. If the total is 1.10, and the only variable is 100, then the solution to the equation is .10, right?
106. It is a simple question to ask to test basic math skills, reading comprehension, and just a general attention check.

[10] This refers to the Berlin numeracy test

107. I think the batting the ball question has been used too much. Besides that it's too easy to answer. The other thing that you see all the time is the long paragraph about questions aren't formed in a vacuum blah blah blah blah blah blah blah and then we're trying to see if you're really reading this. As soon as you see the word vacuum you know it's an attention check. So does that really check your attention?
108. This is a common question that I think most people on Mturk know the answer to. Maybe it's time for requesters to get a new question.
109. Most people doing hits on mturk do a lot of them, so all these surveys that use the same questions that test logical or math skills are wasting their time. Even if the answer wasn't obvious to people, they would eventually figure them out and answer them all correctly.
110. Actually, I'm tired of seeing it. That's not the only one like that. There are many I've seen. I don't look up the answers either. So I'm always wrong. I don't believe in Googling the answer and I'm terrible at math. Thanks.
111. i know the answer is 5 but no idea how it is that number
112. It seems silly. I don't quite see the purpose of asking the question. I feel the same about some of the percentage calculations. For some HITs, it may be useful to get an understanding of the worker's math knowledge but it some cases it just seems like a hoop to jump through.
113. I'm not sure what the purpose is to be honest. Perhaps to gauge general intelligence?
114. I like brain teasers, and I liked this one. I do notice that I see several of the same brain teasers repeated on the surveys that I take. I have never been asked questions about the taking of the tasks before, though.
115. I think the question/answer is over-used and could easily be asked in a bigger variety of ways. I'm OK with the questions and answers, and am not worried too much either way about answering them.
116. I like it till someone ”gets” it then it's bunk The age one is similar logic.
117. The bat-and-ball is a ”trick” question that I find interesting. It's similar to the story of a cat that fell down a 50 foot well. Every day the cat climbs three feet, but falls two feet. The cat's net progress is only 1 foot a day. However, on day 47, the cat reaches the top of the well and climbs out - the ”obvious” answer to how many days it takes the cat to climb out is 50, but the fun part is understanding the actual question.
118. First of all, it's easy because it is simple arithmetic. Second of all, I have seen it on Mechanical Turk many times. Third, I remember it from childhood, beginning with when I was in elementary school. Every time I see it, I wish the requester would come up with something new.
119. I think it would probably better serve researchers who use Mturk to ask a different question that is less familiar
120. It makes me think and I always forget the answer so have to come up with a new one everytime.
121. I think it's annoying, I read the correct answer once someplace, but didn't care enough to remember it, I feel the same way about most of those brain teaser type questions, if I know the answer, great, if I do not, that's fine too
122. I feel that a lot of surveys ask random IQ questions as part of their surveys. I do not mind them. I do wish to be informed beforehand as to whether or not my cognitive abilities will be tested.
123. I feel like I've been getting it wrong the whole time!
124. I am so confused with this question. My kids tried to explain it to me, but I didn't understand it.
125. no opinion, i could care less !

3 CONSTRUCTION TEMPLATES FOR TRANSFORMED VARIANTS OF CRT ITEMS

3.1 Item 1

The joint [measurement] of [A] and [B] is [m_A+m_B]. The [measurement] of [A] is [m_A-m_B] higher than the [measurement] of [B]. What is the [measurement] of [B]?

Requirements:

- [measurement] needs to be additive
- [m_A-m_B] is simple, intuitive natural number
- m_B ¡¡ m_A-m_B

Original:

- [measurement]: price
- [A]: bat
- [B]: ball
- [m_A]=$1.05
- [m_B]=$0.05

Other examples:

- An elephant and a dog eat 303 kg of food together. The elephant eats 300kg more than the dog. How much does the dog eat?
- I bought a computer and monitor for 3,800$. The computer cost 3,000$ more than the monitor Ho much did I pay for the monitor?
- A human stands on the shoulders of a giant. Together, both are 36 feet tall, but the giant is 30 feet taller than the human. How tall is the human?

3.2 Item 2

[x] [productions units] [produce] [x] [product units] in [x] [time units]. How long does it take [$f \cdot x$] [production units] to [produce] [$f \cdot x$] [product units]?

Requirements:

- f is natural number

Original:

- [production unit]: worker
- [product unit]: widget,
- [time unit]: minute
- x=10
- f=10

Other examples:

- Twenty magicians conjure 20 pigeons out of their hats in 20 minutes. How long does it take 60 magicians to conjure 60 pigeons?
- At a large golf range, it takes 30 golfers 30 minutes to hit the greens 30 times in total. How lonng does it take 90 golfers to hit the greens 90 times in total (*but see caveat, below*).
- In a store, 10 sales people are able to deal with 10 shoppers arriving at the same time in ten minutes. How long does it take 200 sales people to deal with 200 shoppers arriving at the same time?

Caveat: Some tasks can be done in parallel and production might not scale with units: 1,000 cooks in a kitchen will not achieve 100 times the number of omelets that 10 cooks can achieve.

3.3 Item 3

A [geometric growth process] starts with and proceeds with a factor of [f] per [time unit] . After t_x [time units] the process [is complete/has reached a simple reference point r_x]. How long does it take to reach [$\frac{r_x}{f^s}$]

Requirements:

- r_x is naturally divisible by f
- $t_x >> 0$
- $\frac{t_x}{g^s} \in \mathbb{N}$
- $\frac{t_x}{g \cdot s} \in \mathbb{N}$

Original:

- [growth process]: lily pad expansion
- [time unit]: days
- [t_x]: 48 days
- [r_x]: lake covered
- f=2
- s=1

Other examples:

- An ant colony doubles its territory in each week. At the end of year (after 52 weeks) the colony covers the entire forest. After how many weeks did the colony cover a quarter of the forest?
- A new robot model is able to build a complete clone of itself once in an hour, and it will spend all of its time building clones. It can start building clones once built and can keep building clones without limits. One robot of this type is placed in a large factory hall. After exactly half a day exactly one half of the factory hall is filled with robots. How many additional hours did it take to fill exactly one quarter of the hall?
- A map has been folded many, many times. Each time you open up one fold it doubles in size. You notice that it covers exactly half of your room after you have unfolded it 42 times. a) After how many

times (including the 42 times) does it cover the full room. b) After how many times did it cover exactly a quarter of your room?

Caveats: Scaling might not be assumed linear, the number of production units might not be responsible for minimum time, example: golfers on golf course

3.4 Other trick questions

- It takes 200 snails 200 seconds to each run a distance of 200 cm. How long does it take 800 snails to each run a distance of 400cm?
- A kitchen receives 100 loaves of bread and 1,000 eggs each morning. Some oil and two eggs are needed for an omelette.
  In this kitchen, 4 cooks are able to produce 400 omelets throughout the day. How many omelets would be produced by 8 cooks?
- In a lake, there is a patch of lily pads. Every day, the patch doubles in size. If it takes 48 days for the patch to cover half the lake, how long would it take for the patch to cover the entire lake [in days]?
- If it takes 10 lakes 10 days to make 10 lily pads, how long would it take 48 lakes to make 48 lily pads [in days]?
- A bat and a ball cost $1.10 in total. The set costs $1.00 more than the ball. How much does the ball cost [in cents]?
- In a lake, there is a patch of lily pads. Every day, the patch quadruples in size. If it takes 48 days for the patch to cover the entire lake, how long would it take for the patch to cover half of the lake [in days]?

REFERENCES

Brañas-Garza, P., Kujal, P., and Lenkei, B. (2015). Cognitive Reflection Test: Whom, how, when ESI Working Paper 15-25

Campitelli, G. and Gerrans, P. (2014). Does the cognitive reflection test measure cognitive reflection? A mathematical modeling approach. *Memory & Cognition* 42, 434–447. doi:10.3758/s13421-013-0367-9

Campitelli, G. and Labollita, M. (2010). Correlations of cognitive reflection with judgments and choices. *Judgment and Decision Making* 5, 182–191

Chandler, J. J. and Paolacci, G. (2017). Lie for a dime: When most prescreening responses are honest but most study participants are impostors. *Social Psychological and Personality Science* , 1948550617698203doi:10.1177/1948550617698203

Cueva, C., Iturbe-Ormaetxe, I., Mata-Pérez, E., Ponti, G., Sartarelli, M., Yu, H., et al. (2016). Cognitive (ir)reflection: New experimental evidence. *Journal of Behavioral and Experimental Economics* 64, 81–93. doi:10.1016/j.socec.2015.09.002

Fagerlin, A., Zikmund-Fisher, B., Ubel, P., Jankovic, A., Derry, H., and Smith, D. (2007). Measuring numeracy without a math test: Development of the Subjective Numeracy Scale (SNS). *Medical Decision Making* 27, 672–680. doi:10.1177/0272989X07304449

Frederick, S. (2005). Cognitive reflection and decision making. *Journal of Economic Perspectives* 19, 25–42. doi:10.1257/089533005775196732

Hastings, J., Madrian, B. C., and Skimmyhorn, W. L. (2013). Financial literacy, financial education, and economic outcomes. *Annual Review of Economics* 5, 347–373. doi:10.1146/annurev-economics-082312-125807

Hauser, D. J. and Schwarz, N. (2015). It's a trap! Instructional manipulation checks prompt systematic thinking on "tricky" tasks. *SAGE Open* 5, 2158244015584617. doi:10.1177/2158244015584617

Holyoak, K. J. and Koh, K. (1987). Surface and structural similarity in analogical transfer. *Memory & Cognition* 15, 332–340

Irvine, S. H. (2002). The foundations of item generation for mass testing. In *Item generation for test development*, eds. . Irvine, S. H. and P. C. Kyllonnen (Mahwah, NJ: Lawrence Erlbaum). 3–34

Kennedy, R., Clifford, S., Burleigh, T., Waggoner, P., and Jewel, R. (2018). How Venezuela's economic crisis is undermining social science research—about everything. *Washington Post*

Krosnick, J. A. (1991). Response strategies for coping with the cognitive demands of attitude measures in surveys. *Applied Cognitive Psychology* 5, 213–236. doi:10.1002/acp.2350050305

Lee, H. S., Betts, S., and Anderson, J. R. (2015). Not taking the easy road: When similarity hurts learning. *Memory & Cognition* 43, 939–952. doi:10.3758/s13421-015-0509-3

Meyer, A., Zhou, E., and Frederick, S. (2018). The non-effects of repeated exposure to the Cognitive Reflection Test. *Judgment and Decision Making* 13, 246–259

Milland, K. (2015). Lily pads and bats & balls - what survey answers have you memorized due to exposure? *TurkerNation*

Morley, M. E., Bridgeman, B., and Lawless, R. R. (2004). Transfer between variants of quantitative items. *ETS Research Report Series* 2004, i–27

Oppenheimer, D. M., Meyvis, T., and Davidenko, N. (2009). Instructional manipulation checks: Detecting satisficing to increase statistical power. *Journal of Experimental Social Psychology* 45, 867–872

Peer, E., Vosgerau, J., and Acquisti, A. (2014). Reputation as a sufficient condition for data quality on amazon mechanical turk. *Behavior Research Methods* 46, 1023–1031. doi:10.3758/s13428-015-0578-z

Raoelison, M. and De Neys, W. (2019). Do we de-bias ourselves?: The impact of repeated presentation on the bat-and-ball problem. *Judgment and Decision Making* 14, 170–178

Reed, S. K. (1987). A structure-mapping model for word problems. *Journal of Experimental Psychology: Learning, Memory, and Cognition* 13, 124. doi:10.1037/0278-7393.13.1.124

Ross, B. H. (1989). Distinguishing types of superficial similarities: Different effects on the access and use of earlier problems. *Journal of Experimental Psychology: Learning, Memory, and Cognition* 15, 456–468. doi:10.1037/0278-7393.15.3.456

Toplak, M. E., West, R. F., and Stanovich, K. E. (2014). Assessing miserly information processing: An expansion of the Cognitive Reflection Test. *Thinking & Reasoning* 20, 147–168. doi:10.1080/13546783.2013.844729
